# Supplementary material for: Benchmarking strategies for cross-species integration of single-cell RNA sequencing data
Source: Nat Commun. 2023 Oct 14;14:6495. doi: 10.1038/s41467-023-41855-w (PMC10576752; doi:10.1038/s41467-023-41855-w)
Supplement: Supplementary file 1 — Supplementary Information [file 41467_2023_41855_MOESM1_ESM.pdf]

Supplementary Information

Benchmarking strategies for cross-species integration of single-cell RNA sequencing data

Yuyao Song [1, *], Zhichao Miao [1, 2], Alvis Brazma [1], Irene Papatheodorou [1, *]

1 European Molecular Biology Laboratory-European Bioinformatics Institute (EMBL-EBI), Wellcome Genome Campus, Hinxton, United Kingdom

2 Guangzhou Laboratory, Guangzhou International Bio Island, Guangzhou 510005, China

* Corresponding authors

Emails:

Yuyao Song: ysong@ebi.ac.uk

Irene Papatheodorou: irenep@ebi.ac.uk

Address:

European Molecular Biology Laboratory-European Bioinformatics Institute (EMBL-EBI)

Wellcome Genome Campus

Hinxton, Cambridgeshire

CB10 1SD

United Kingdom

Tel:

+44 (0)1223 494 444

Table of Contents

Supplementary Figures **3**

Supplementary Methods **71**

Principal of batch correction metrics 71
(1) Principal component regression (PCR) 71
(2) Batch average silhouette width (bASW) 71
(3) Graph connectivity (GC) 72
(4) K-nearest neighbour batch effect test (kBET) 72
Principal of biology conservation metrics 73
(1) Cell type ASW (cASW) 73
(2) Normalised Mutual Information (NMI) 73
(3) Adjusted Rand Index (ARI) 73
(4) Isolated label F1 score (iso_F1) 73
(5) Trajectory conservation (Traj) 74

Supplementary Notes **74**

(1) Batch correction metrics and biology conservation metrics are not applicable for SAMap 74
(2) Comprehending observed non-one-to-one cell type correspondence on integrated data 75
(3) Other classifiers currently supported by ALCS 77
(4) Applying a kNN classifier for ALCS 77
(a) Different kNN graph output generated by algorithms 77
(b) Applying a kNN classifier on distance matrices 78
(c) Training and testing phases of the kNN classifier 79
(d) Conclusions on applying a kNN classifier for ALCS 81

Supplementary References **81**

Supplementary Figures

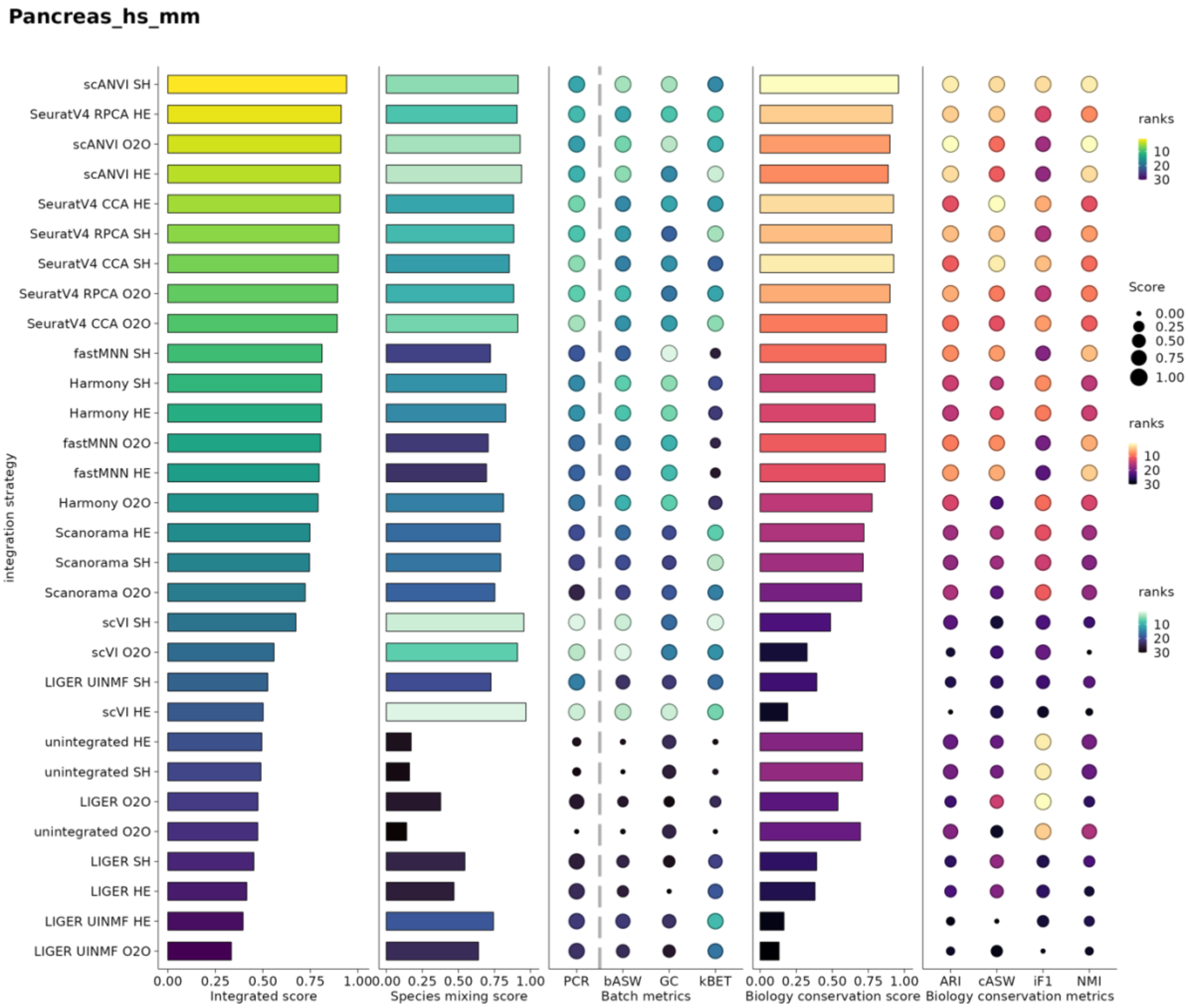

Supplementary Figure 1 Benchmarking scores and metrics of different integration strategies on cross-species analysis in the Pancreas_hs_mm task. Batch removal metrics and biological conservation metrics are min-max scaled per task. The species mixing score is the average of 4 batch removal metrics, while the biological conservation score is the average of 4 biology conservation metrics. The integrated score is a weighted average of species mixing score and biology conservation score with 40/60 weighting. Grey dash lines indicate a distinction between metrics that do not rely on cell type annotation (PCR) with metrics relying on cell type annotation. O2O, only uses one-to-one orthologs; HE, one-to-one orthologs plus one-to-many and many-to-many orthologs matched by higher average expression level; SH, one-to-one orthologs plus one-to-many and many-to-many orthologs matched by stronger homology confidence; PCR: principal component regression; bASW: batch average silhouette width, GC: graph connectivity, kBET: k-nearest neighbour batch effect test,

ARI: adjusted rand index, cASW: cell type average silhouette width, NMI: normalised mutual information of cell label, iF1: isolated label F1 score.

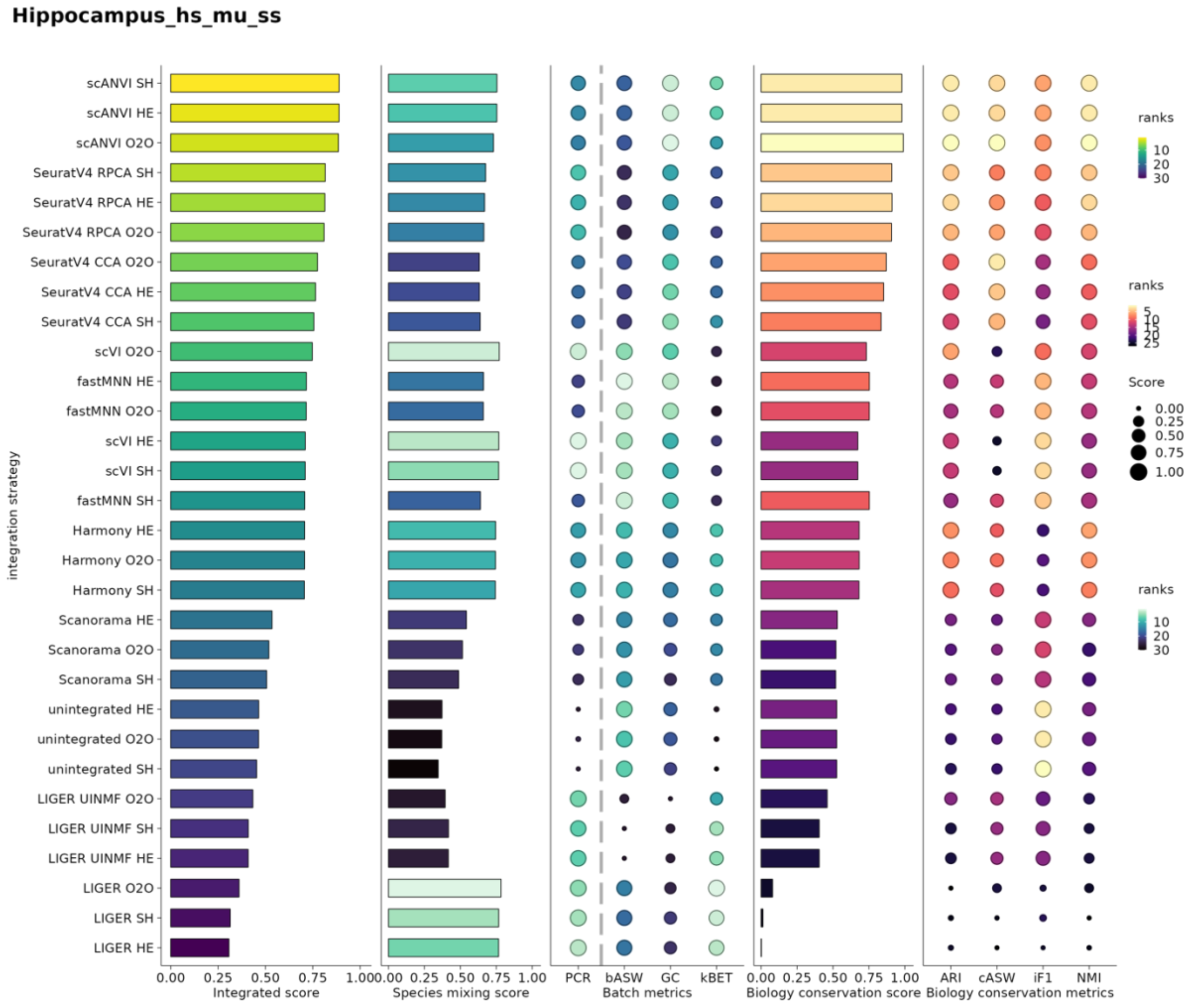

Supplementary Figure 2 Benchmarking scores and metrics of different integration strategies on cross-species analysis in the Hippocampus_hs_mu_ss task. Batch removal metrics and biological conservation metrics are min-max scaled per task. The species mixing score is the average of 4 batch removal metrics, while the biological conservation score is the average of 4 biology conservation metrics. The integrated score is a weighted average of species mixing score and biology conservation score with 40/60 weighting. Grey dash lines indicate a distinction between metrics that do not rely on cell type annotation (PCR) with metrics relying on cell type annotation. O2O, only uses one-to-one orthologs; HE, one-to-one orthologs plus one-to-many and many-to-many orthologs matched by higher average expression level; SH, one-to-one orthologs plus one-to-many and many-to-many

orthologs matched by stronger homology confidence; PCR: principal component regression; bASW: batch average silhouette width, GC: graph connectivity, kBET: k-nearest neighbour batch effect test, ARI: adjusted rand index, cASW: cell type average silhouette width, NMI: normalised mutual information of cell label, iF1: isolated label F1 score.

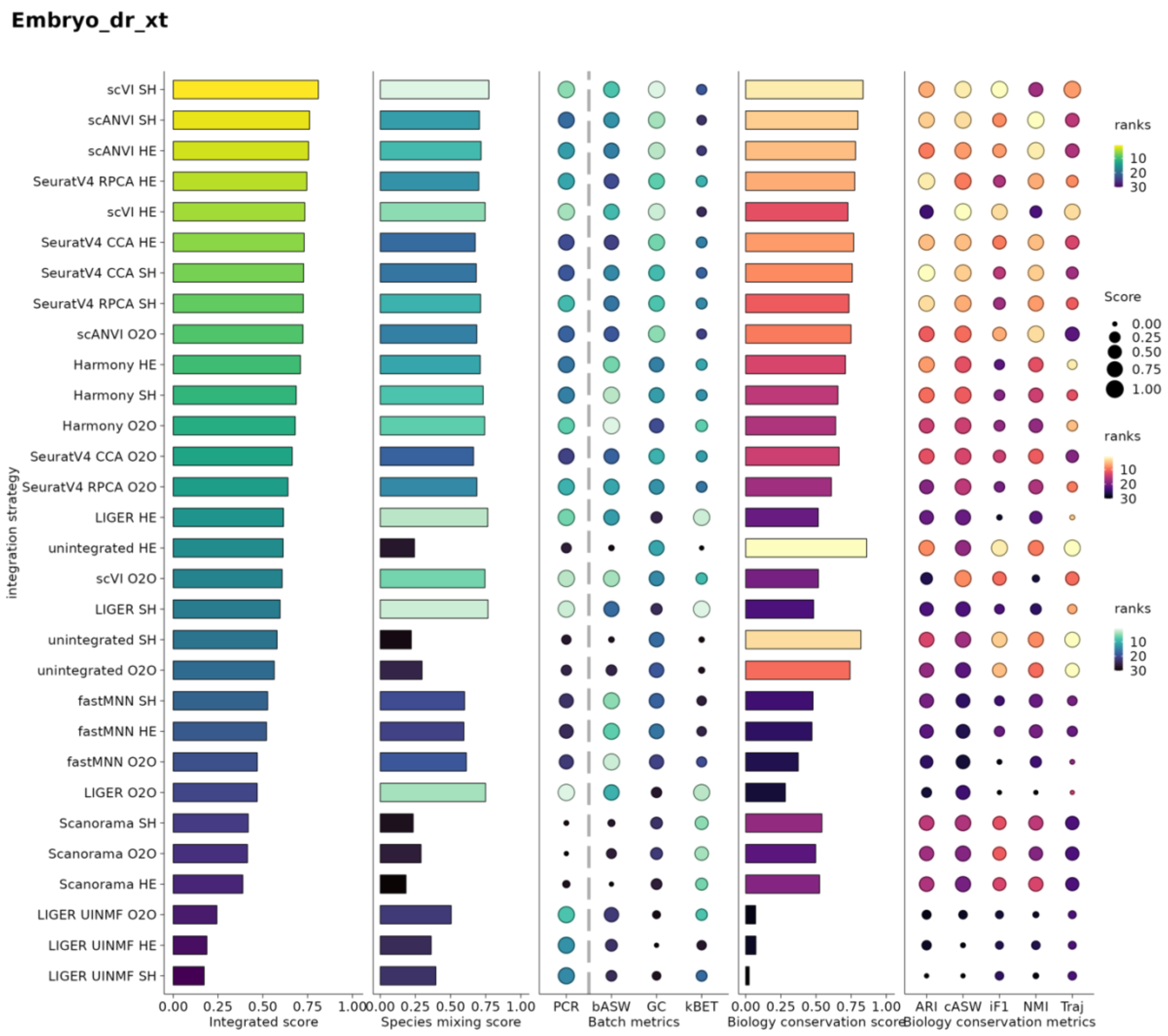

Supplementary Figure 3 Benchmarking scores and metrics of different integration strategies on cross-species analysis in the Embryo_dr_xt task. Batch removal metrics and biological conservation metrics are min-max scaled per task. The species mixing score is the average of 4 batch removal metrics, while the biological conservation score is the average of 5 biology conservation metrics. Traj score is only applicable to this task. The integrated score is a weighted average of species mixing score and biology conservation score with 40/60 weighting. Grey dash lines indicate a

distinction between metrics that do not rely on cell type annotation (PCR) with metrics relying on cell type annotation. O2O, only uses one-to-one orthologs; HE, one-to-one orthologs plus one-to-many and many-to-many orthologs matched by higher average expression level; SH, one-to-one orthologs plus one-to-many and many-to-many orthologs matched by stronger homology confidence; PCR: principal component regression; bASW: batch average silhouette width, GC: graph connectivity, kBET: k-nearest neighbour batch effect test, ARI: adjusted rand index, cASW: cell type average silhouette width, NMI: normalised mutual information of cell label, iF1: isolated label F1 score, Traj: trajectory conservation score.

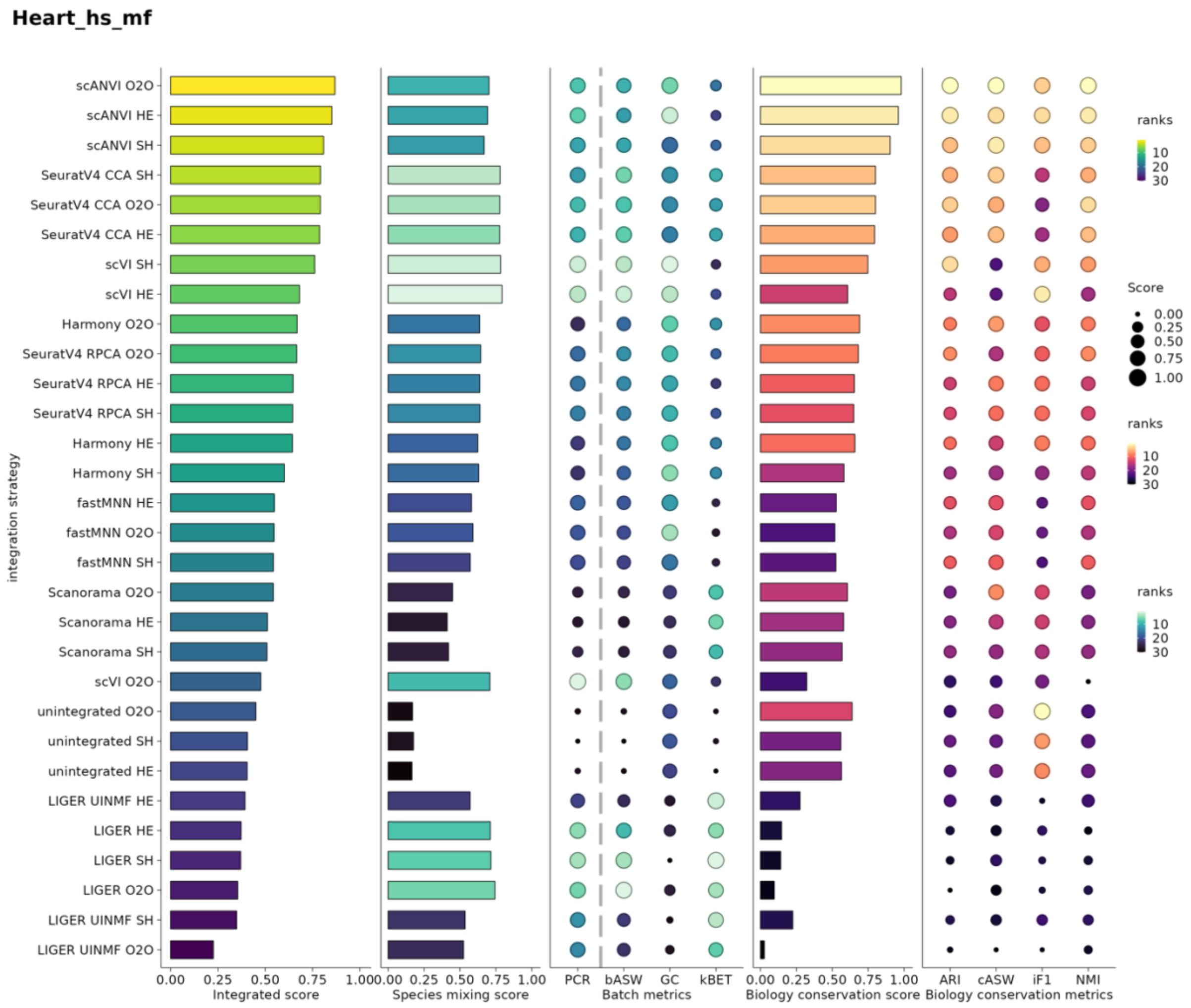

Supplementary Figure 4 Benchmarking scores and metrics of different integration strategies on cross-species analysis in the Heart_hs_mf task. Batch removal metrics and biological conservation metrics are min-max scaled per task. The species mixing score is the average of 4 batch removal

metrics, while the biological conservation score is the average of 5 biology conservation metrics. The integrated score is a weighted average of species mixing score and biology conservation score with 40/60 weighting. Grey dash lines indicate a distinction between metrics that do not rely on cell type annotation (PCR) with metrics relying on cell type annotation. O2O, only uses one-to-one orthologs; HE, one-to-one orthologs plus one-to-many and many-to-many orthologs matched by higher average expression level; SH, one-to-one orthologs plus one-to-many and many-to-many orthologs matched by stronger homology confidence; PCR: principal component regression; bASW: batch average silhouette width, GC: graph connectivity, kBET: k-nearest neighbour batch effect test, ARI: adjusted rand index, cASW: cell type average silhouette width, NMI: normalised mutual information of cell label, iF1: isolated label F1 score.

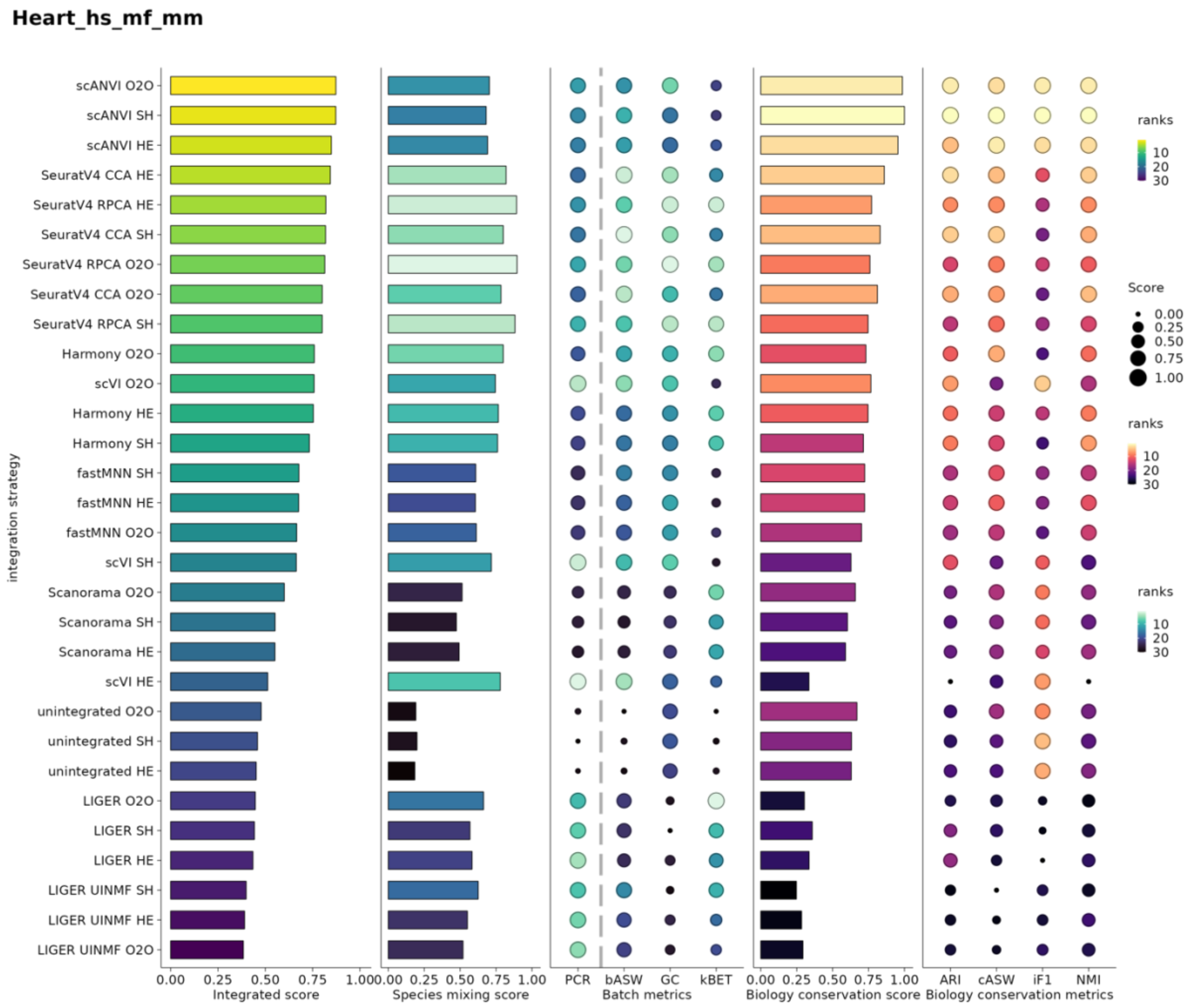

Supplementary Figure 5 Benchmarking scores and metrics of different integration strategies on cross-species analysis in the Heart_hs_mf_mm task. Batch removal metrics and biological conservation metrics are min-max scaled per task. The species mixing score is the average of 4 batch removal metrics, while the biological conservation score is the average of 5 biology conservation metrics. The integrated score is a weighted average of species mixing score and biology conservation score with 40/60 weighting. Grey dash lines indicate a distinction between metrics that do not rely on cell type annotation (PCR) with metrics relying on cell type annotation. O2O, only uses one-to-one orthologs; HE, one-to-one orthologs plus one-to-many and many-to-many orthologs matched by higher average expression level; SH, one-to-one orthologs plus one-to-many and many-to-many orthologs matched by stronger homology confidence; PCR: principal component regression; bASW: batch average silhouette width, GC: graph connectivity, kBET: k-nearest neighbour batch effect test, ARI: adjusted rand index, cASW: cell type average silhouette width, NMI: normalised mutual information of cell label, iF1: isolated label F1 score.

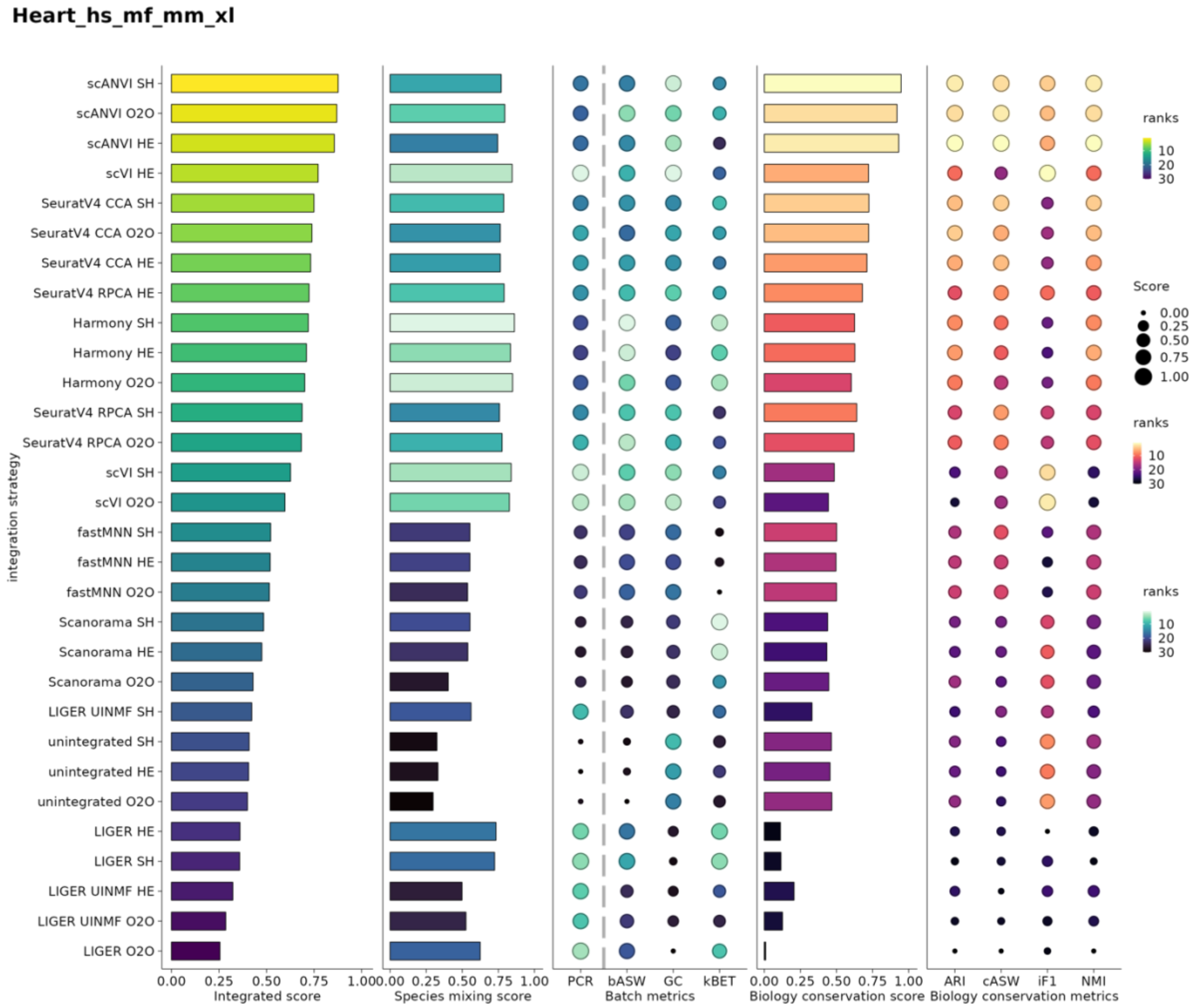

Supplementary Figure 6 Benchmarking scores and metrics of different integration strategies on cross-species analysis in the Heart_hs_mf_mm_xl task. Batch removal metrics and biological conservation metrics are min-max scaled per task. The species mixing score is the average of 4 batch removal metrics, while the biological conservation score is the average of 5 biology conservation metrics. The integrated score is a weighted average of species mixing score and biology conservation score with 40/60 weighting. Grey dash lines indicate a distinction between metrics that do not rely on cell type annotation (PCR) with metrics relying on cell type annotation. O2O, only uses one-to-one orthologs; HE, one-to-one orthologs plus one-to-many and many-to-many orthologs matched by higher average expression level; SH, one-to-one orthologs plus one-to-many and many-to-many orthologs matched by stronger homology confidence; PCR: principal component regression; bASW: batch average silhouette width, GC: graph connectivity, kBET: k-nearest neighbour batch effect test,

ARI: adjusted rand index, cASW: cell type average silhouette width, NMI: normalised mutual information of cell label, iF1: isolated label F1 score.

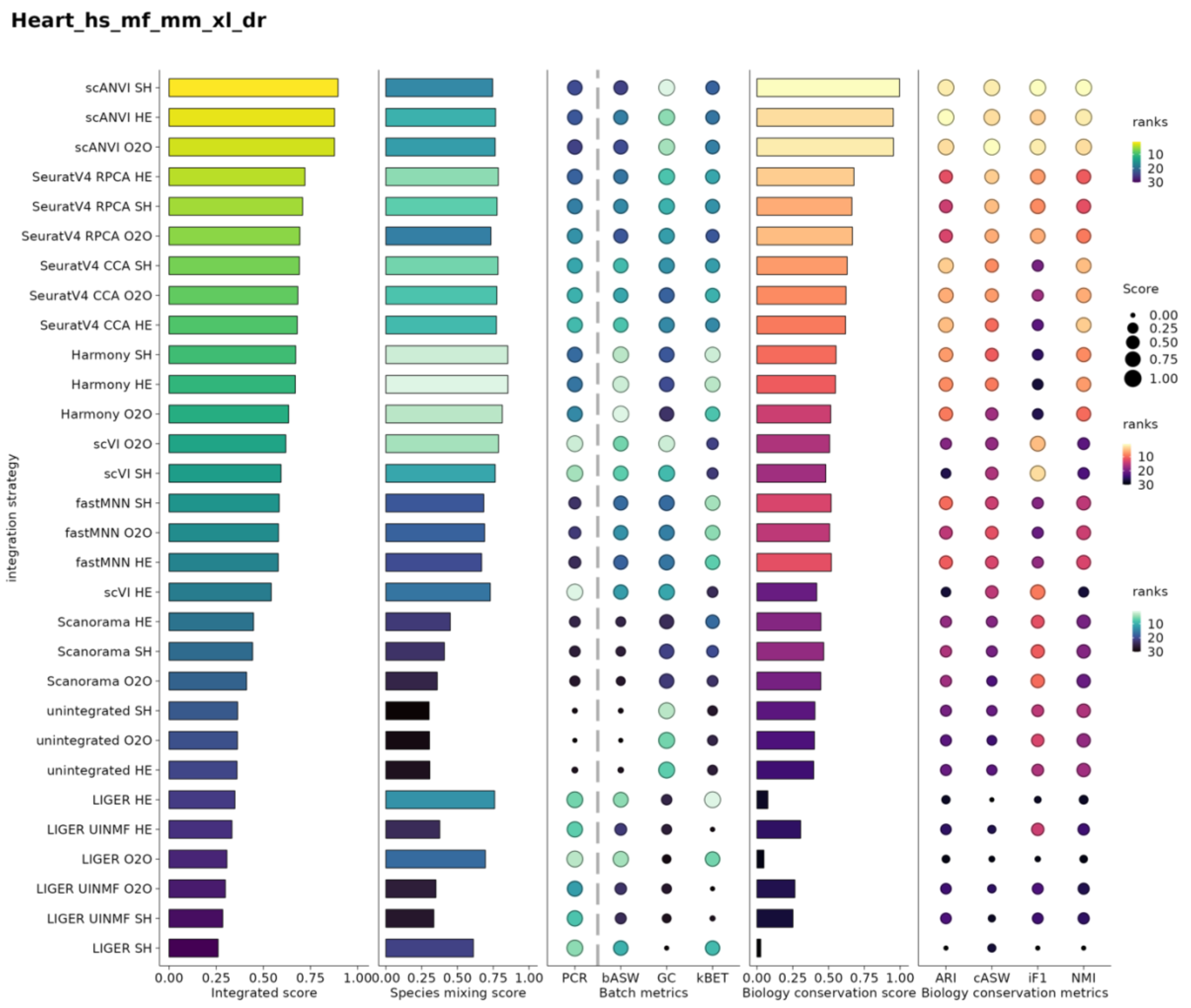

Supplementary Figure 7 Benchmarking scores and metrics of different integration strategies on cross-species analysis in the Heart_hs_mf_mm_xl_dr task. Batch removal metrics and biological conservation metrics are min-max scaled per task. The species mixing score is the average of 4 batch removal metrics, while the biological conservation score is the average of 5 biology conservation metrics. The integrated score is a weighted average of species mixing score and biology conservation score with 40/60 weighting. Grey dash lines indicate a distinction between metrics that do not rely on cell type annotation (PCR) with metrics relying on cell type annotation. O2O, only uses one-to-one orthologs; HE, one-to-one orthologs plus one-to-many and many-to-many orthologs matched by higher average expression level; SH, one-to-one orthologs plus one-to-many and many-to-many

orthologs matched by stronger homology confidence; PCR: principal component regression; bASW: batch average silhouette width, GC: graph connectivity, kBET: k-nearest neighbour batch effect test, ARI: adjusted rand index, cASW: cell type average silhouette width, NMI: normalised mutual information of cell label, iF1: isolated label F1 score.

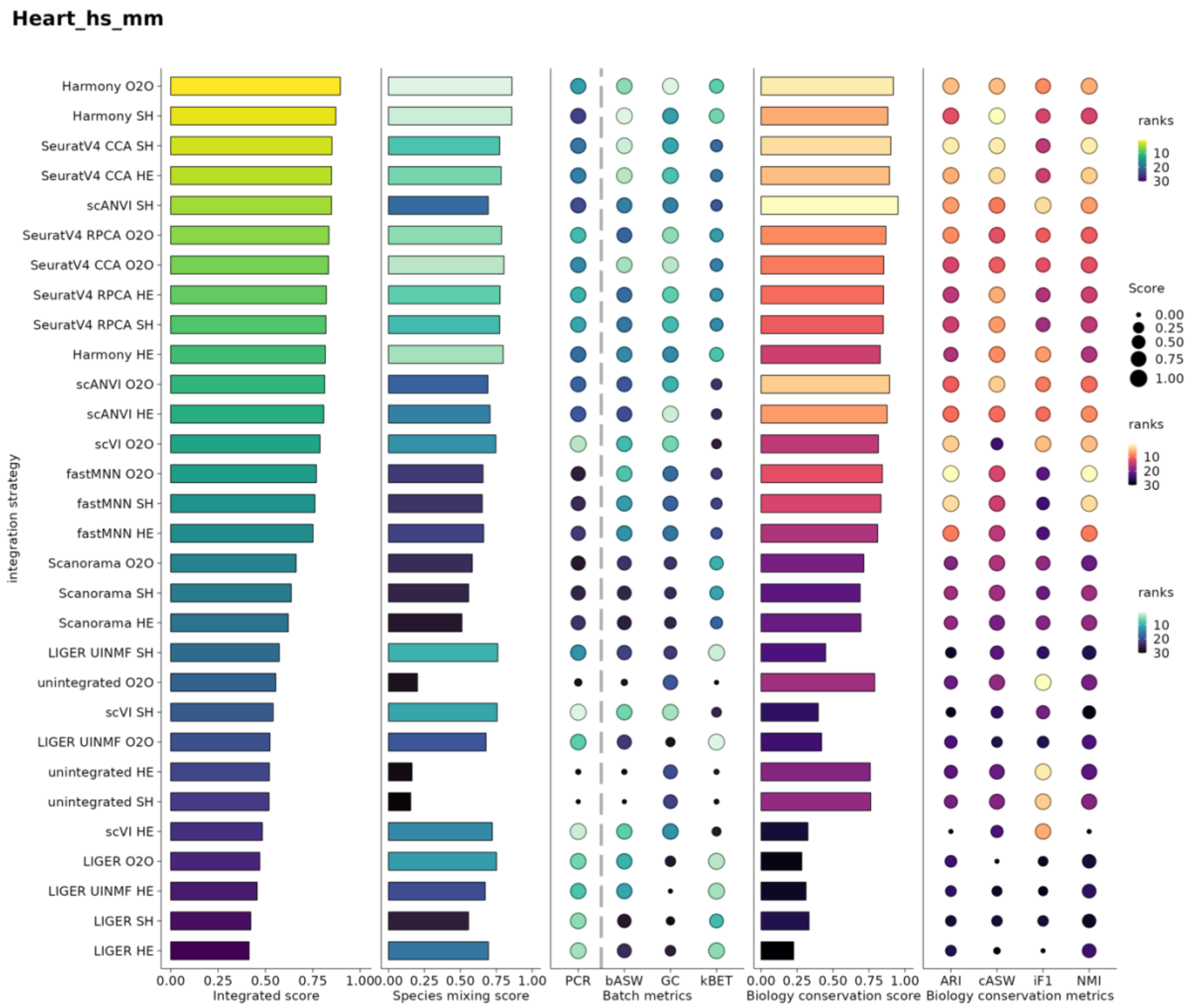

Supplementary Figure 8 Benchmarking scores and metrics of different integration strategies on cross-species analysis in the Heart_hs_mm task. Batch removal metrics and biological conservation metrics are min-max scaled per task. The species mixing score is the average of 4 batch removal metrics, while the biological conservation score is the average of 5 biology conservation metrics. The integrated score is a weighted average of species mixing score and biology conservation score with 40/60 weighting. Grey dash lines indicate a distinction between metrics that do not rely on cell type annotation (PCR) with metrics relying on cell type annotation. O2O, only uses one-to-one orthologs;

HE, one-to-one orthologs plus one-to-many and many-to-many orthologs matched by higher average expression level; SH, one-to-one orthologs plus one-to-many and many-to-many orthologs matched by stronger homology confidence; PCR: principal component regression; bASW: batch average silhouette width, GC: graph connectivity, kBET: k-nearest neighbour batch effect test, ARI: adjusted rand index, cASW: cell type average silhouette width, NMI: normalised mutual information of cell label, iF1: isolated label F1 score.

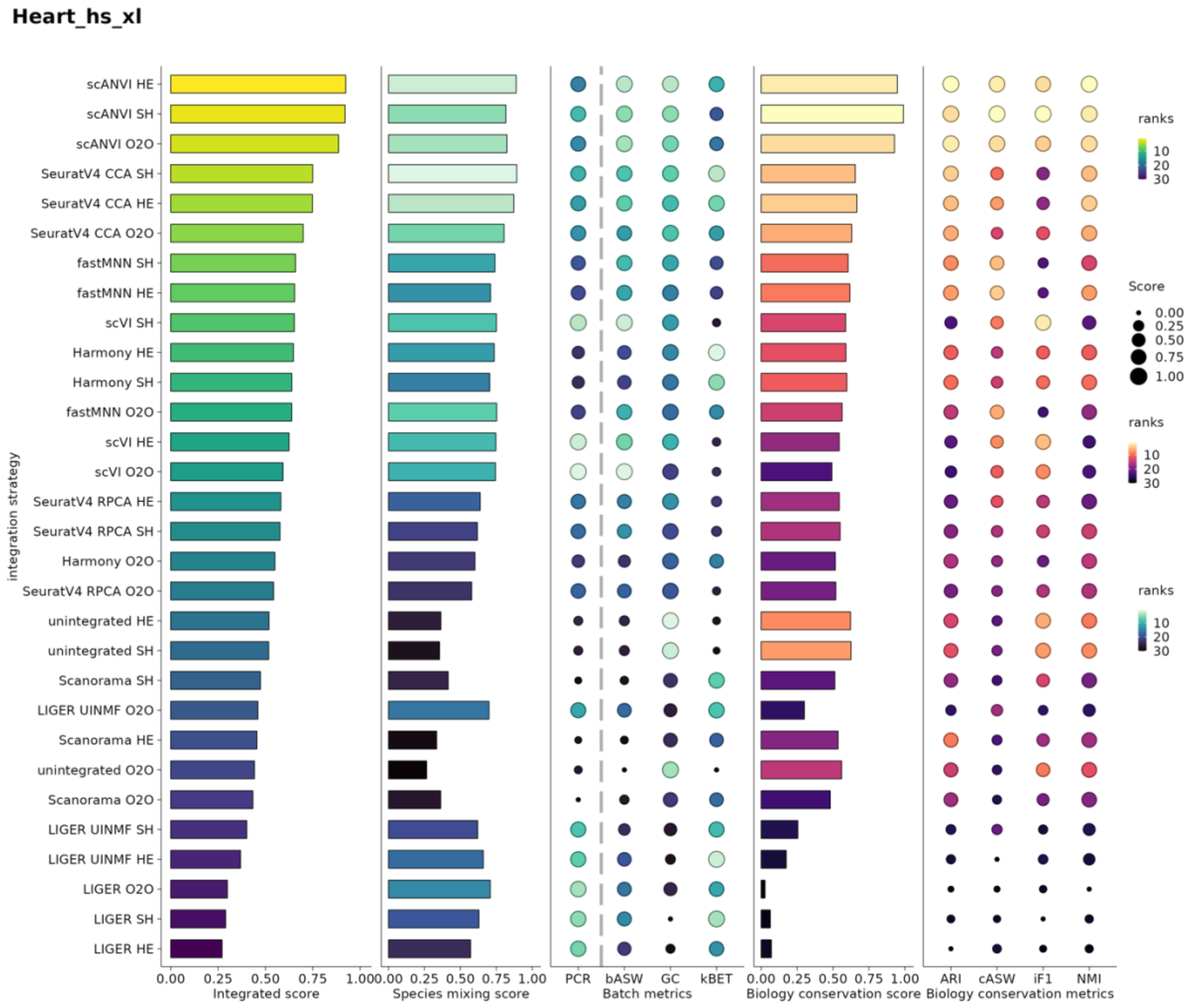

Supplementary Figure 9 Benchmarking scores and metrics of different integration strategies on cross-species analysis in the Heart_hs_xl task. Batch removal metrics and biological conservation metrics are min-max scaled per task. The species mixing score is the average of 4 batch removal metrics, while the biological conservation score is the average of 5 biology conservation metrics. The integrated score is a weighted average of species mixing score and biology conservation score with

40/60 weighting. Grey dash lines indicate a distinction between metrics that do not rely on cell type annotation (PCR) with metrics relying on cell type annotation. O2O, only uses one-to-one orthologs; HE, one-to-one orthologs plus one-to-many and many-to-many orthologs matched by higher average expression level; SH, one-to-one orthologs plus one-to-many and many-to-many orthologs matched by stronger homology confidence; PCR: principal component regression; bASW: batch average silhouette width, GC: graph connectivity, kBET: k-nearest neighbour batch effect test, ARI: adjusted rand index, cASW: cell type average silhouette width, NMI: normalised mutual information of cell label, iF1: isolated label F1 score.

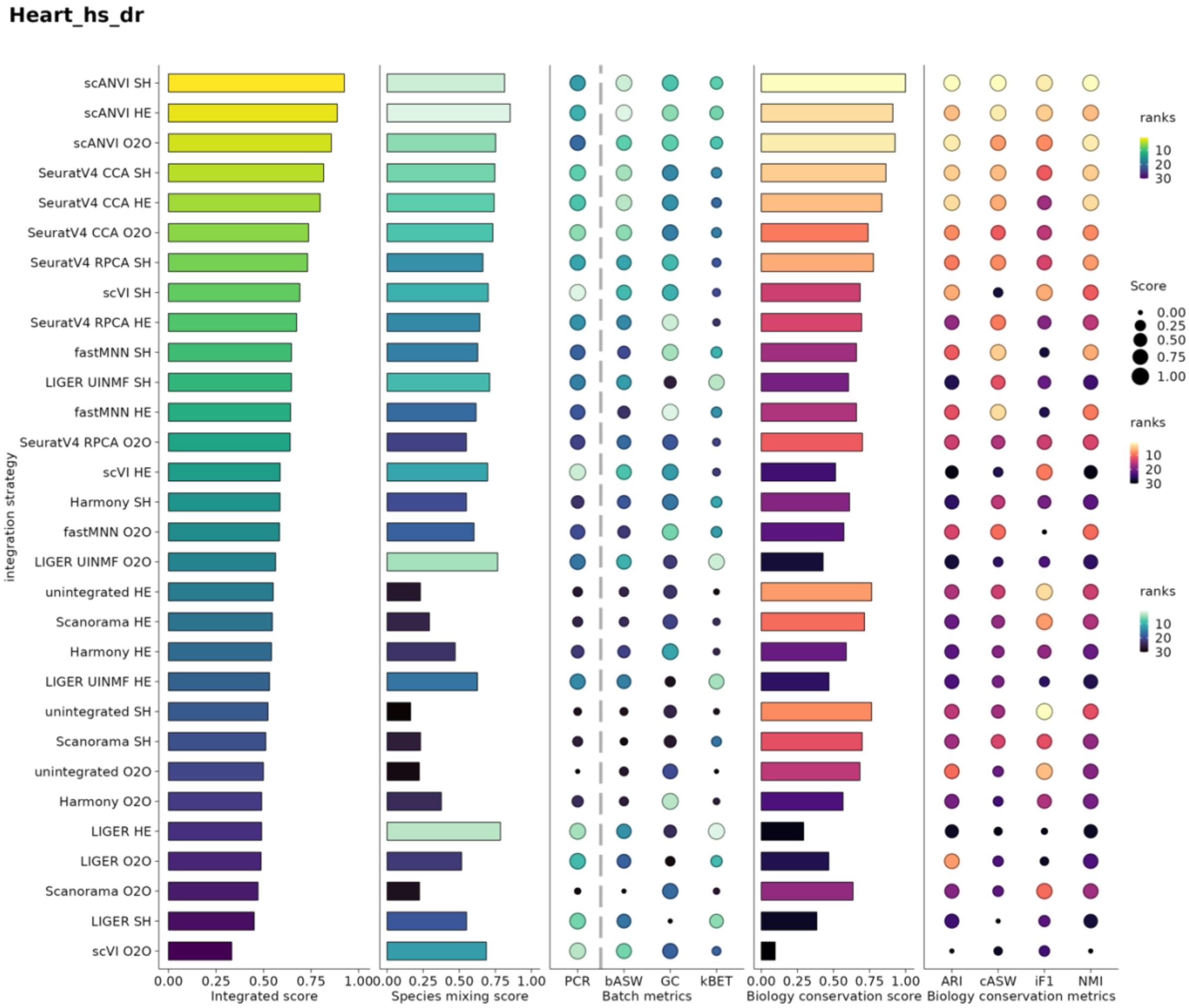

Supplementary Figure 10 Benchmarking scores and metrics of different integration strategies on cross-species analysis in the Heart_hs_dr task. Batch removal metrics and biological conservation metrics are min-max scaled per task. The species mixing score is the average of 4 batch

removal metrics, while the biological conservation score is the average of 5 biology conservation metrics. The integrated score is a weighted average of species mixing score and biology conservation score with 40/60 weighting. Grey dash lines indicate a distinction between metrics that do not rely on cell type annotation (PCR) with metrics relying on cell type annotation. O2O, only uses one-to-one orthologs; HE, one-to-one orthologs plus one-to-many and many-to-many orthologs matched by higher average expression level; SH, one-to-one orthologs plus one-to-many and many-to-many orthologs matched by stronger homology confidence; PCR: principal component regression; bASW: batch average silhouette width, GC: graph connectivity, kBET: k-nearest neighbour batch effect test, ARI: adjusted rand index, cASW: cell type average silhouette width, NMI: normalised mutual information of cell label, iF1: isolated label F1 score.

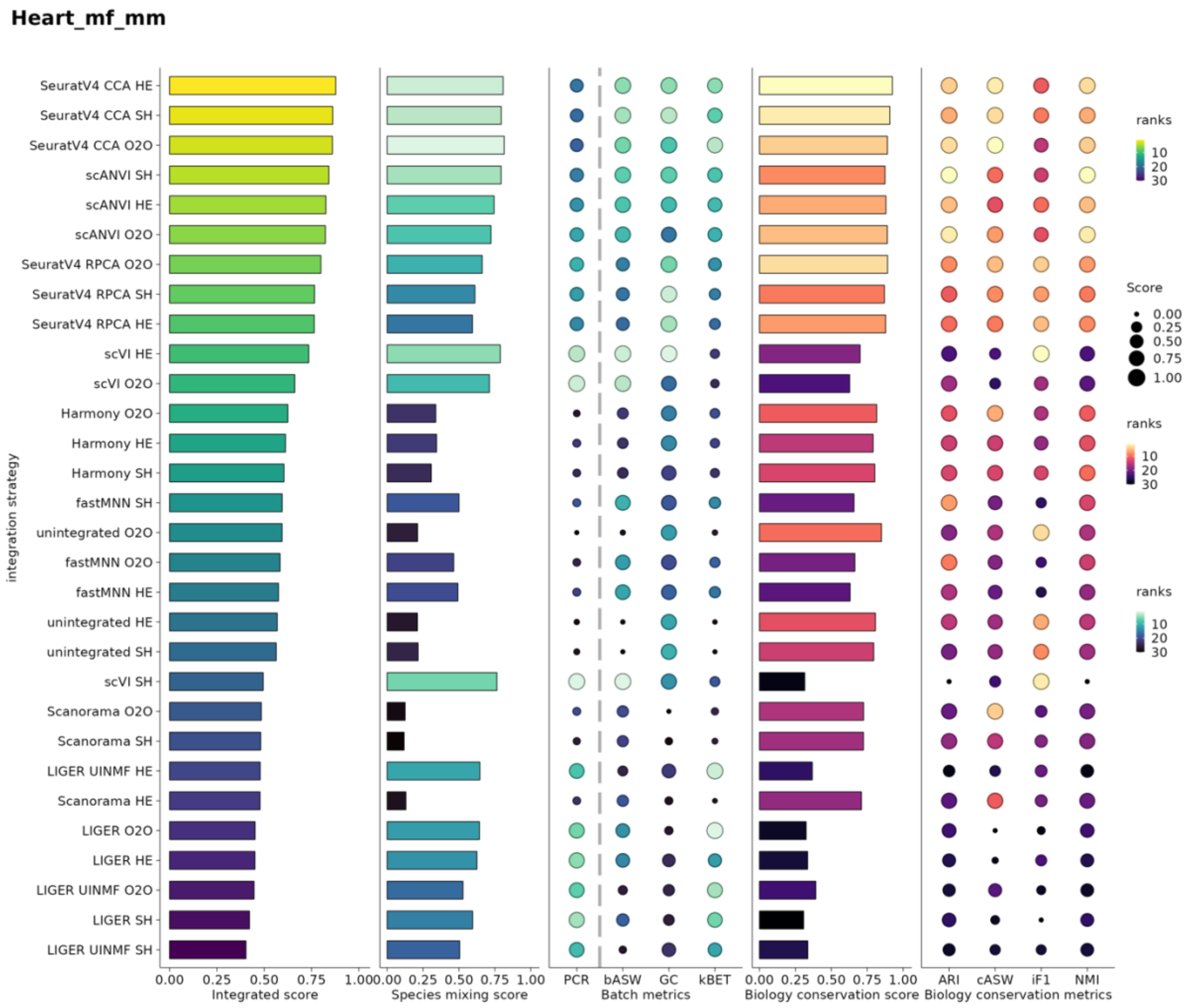

Supplementary Figure 11 Benchmarking scores and metrics of different integration strategies on cross-species analysis in the Heart_mf_mm task. Batch removal metrics and biological conservation metrics are min-max scaled per task. The species mixing score is the average of 4 batch removal metrics, while the biological conservation score is the average of 5 biology conservation metrics. The integrated score is a weighted average of species mixing score and biology conservation score with 40/60 weighting. Grey dash lines indicate a distinction between metrics that do not rely on cell type annotation (PCR) with metrics relying on cell type annotation. O2O, only uses one-to-one orthologs; HE, one-to-one orthologs plus one-to-many and many-to-many orthologs matched by higher average expression level; SH, one-to-one orthologs plus one-to-many and many-to-many orthologs matched by stronger homology confidence; PCR: principal component regression; bASW: batch average silhouette width, GC: graph connectivity, kBET: k-nearest neighbour batch effect test, ARI: adjusted rand index, cASW: cell type average silhouette width, NMI: normalised mutual information of cell label, iF1: isolated label F1 score.

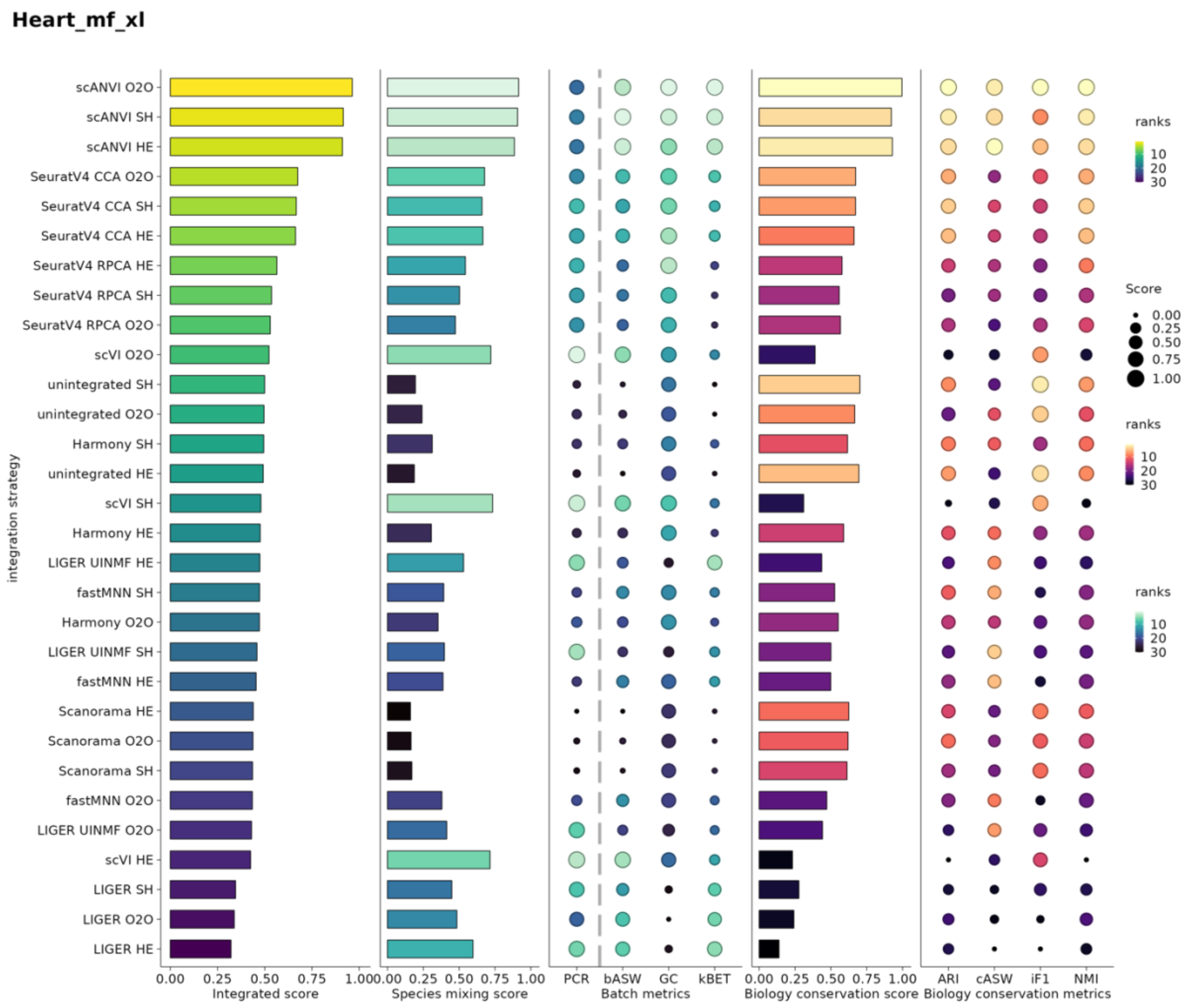

Supplementary Figure 12 Benchmarking scores and metrics of different integration strategies on cross-species analysis in the Heart_mf_xl task. Batch removal metrics and biological conservation metrics are min-max scaled per task. The species mixing score is the average of 4 batch removal metrics, while the biological conservation score is the average of 5 biology conservation metrics. The integrated score is a weighted average of species mixing score and biology conservation score with 40/60 weighting. Grey dash lines indicate a distinction between metrics that do not rely on cell type annotation (PCR) with metrics relying on cell type annotation. O2O, only uses one-to-one orthologs; HE, one-to-one orthologs plus one-to-many and many-to-many orthologs matched by higher average expression level; SH, one-to-one orthologs plus one-to-many and many-to-many orthologs matched by stronger homology confidence; PCR: principal component regression; bASW: batch average silhouette width, GC: graph connectivity, kBET: k-nearest neighbour batch effect test,

ARI: adjusted rand index, cASW: cell type average silhouette width, NMI: normalised mutual information of cell label, iF1: isolated label F1 score.

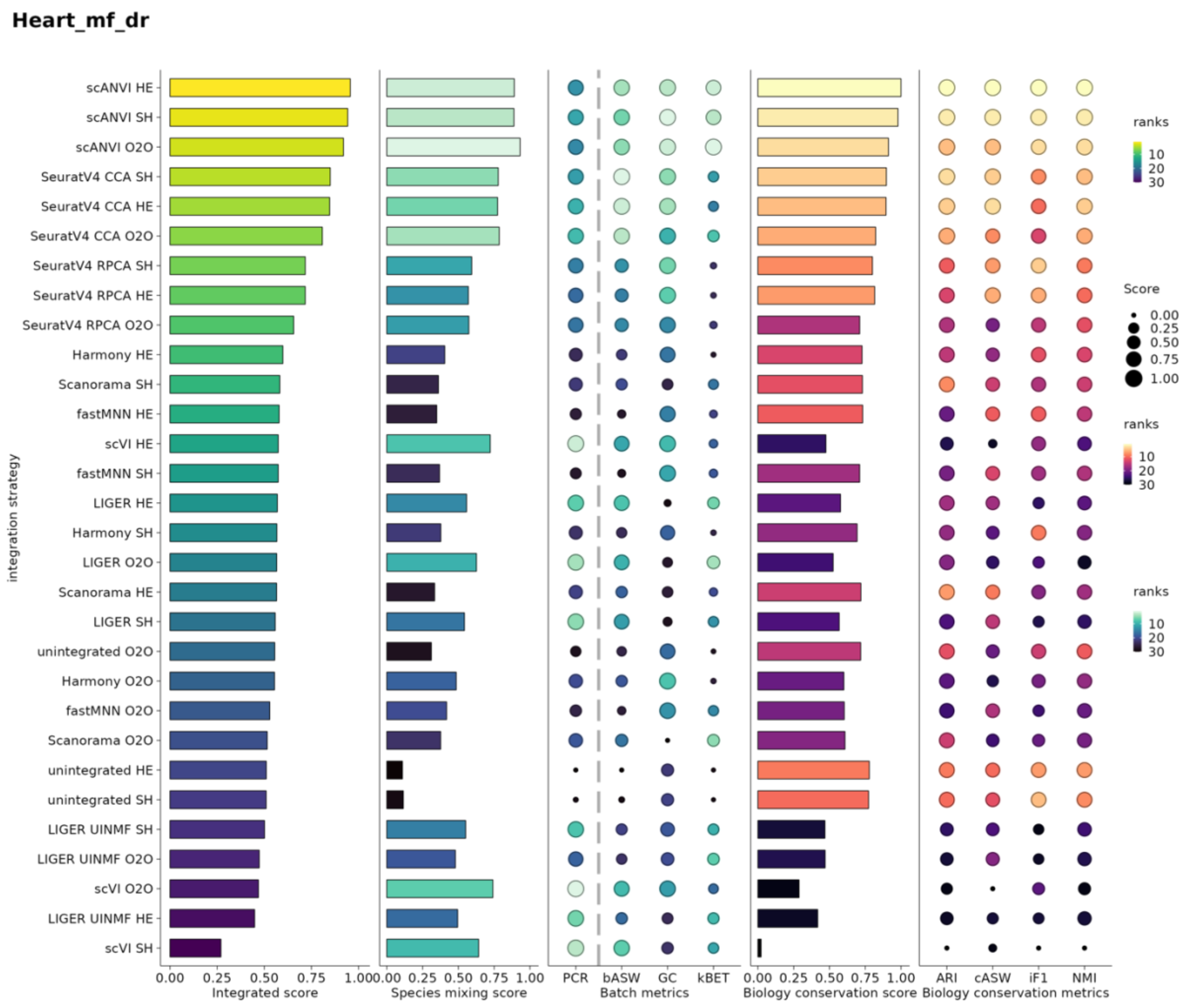

Supplementary Figure 13 Benchmarking scores and metrics of different integration strategies on cross-species analysis in the Heart_mf_dr task. Batch removal metrics and biological conservation metrics are min-max scaled per task. The species mixing score is the average of 4 batch removal metrics, while the biological conservation score is the average of 5 biology conservation metrics. The integrated score is a weighted average of species mixing score and biology conservation score with 40/60 weighting. Grey dash lines indicate a distinction between metrics that do not rely on cell type annotation (PCR) with metrics relying on cell type annotation. O2O, only uses one-to-one orthologs; HE, one-to-one orthologs plus one-to-many and many-to-many orthologs matched by higher average expression level; SH, one-to-one orthologs plus one-to-many and many-to-many

orthologs matched by stronger homology confidence; PCR: principal component regression; bASW: batch average silhouette width, GC: graph connectivity, kBET: k-nearest neighbour batch effect test, ARI: adjusted rand index, cASW: cell type average silhouette width, NMI: normalised mutual information of cell label, iF1: isolated label F1 score.

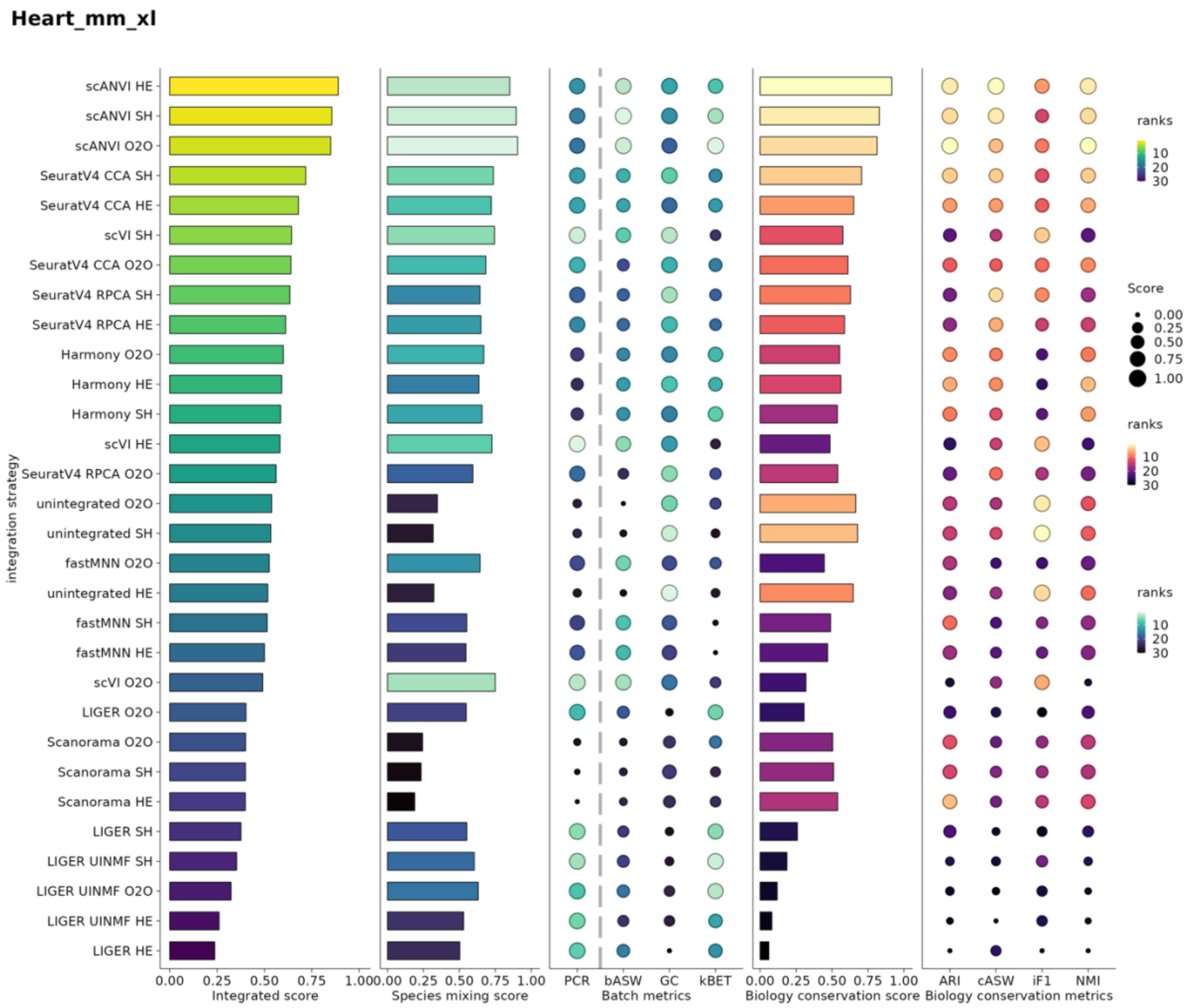

Supplementary Figure 14 Benchmarking scores and metrics of different integration strategies on cross-species analysis in the Heart_mm_xl task. Batch removal metrics and biological conservation metrics are min-max scaled per task. The species mixing score is the average of 4 batch removal metrics, while the biological conservation score is the average of 5 biology conservation metrics. The integrated score is a weighted average of species mixing score and biology conservation score with 40/60 weighting. Grey dash lines indicate a distinction between metrics that do not rely on cell type annotation (PCR) with metrics relying on cell type annotation. O2O, only uses one-to-one

orthologs; HE, one-to-one orthologs plus one-to-many and many-to-many orthologs matched by higher average expression level; SH, one-to-one orthologs plus one-to-many and many-to-many orthologs matched by stronger homology confidence; PCR: principal component regression; bASW: batch average silhouette width, GC: graph connectivity, kBET: k-nearest neighbour batch effect test, ARI: adjusted rand index, cASW: cell type average silhouette width, NMI: normalised mutual information of cell label, iF1: isolated label F1 score.

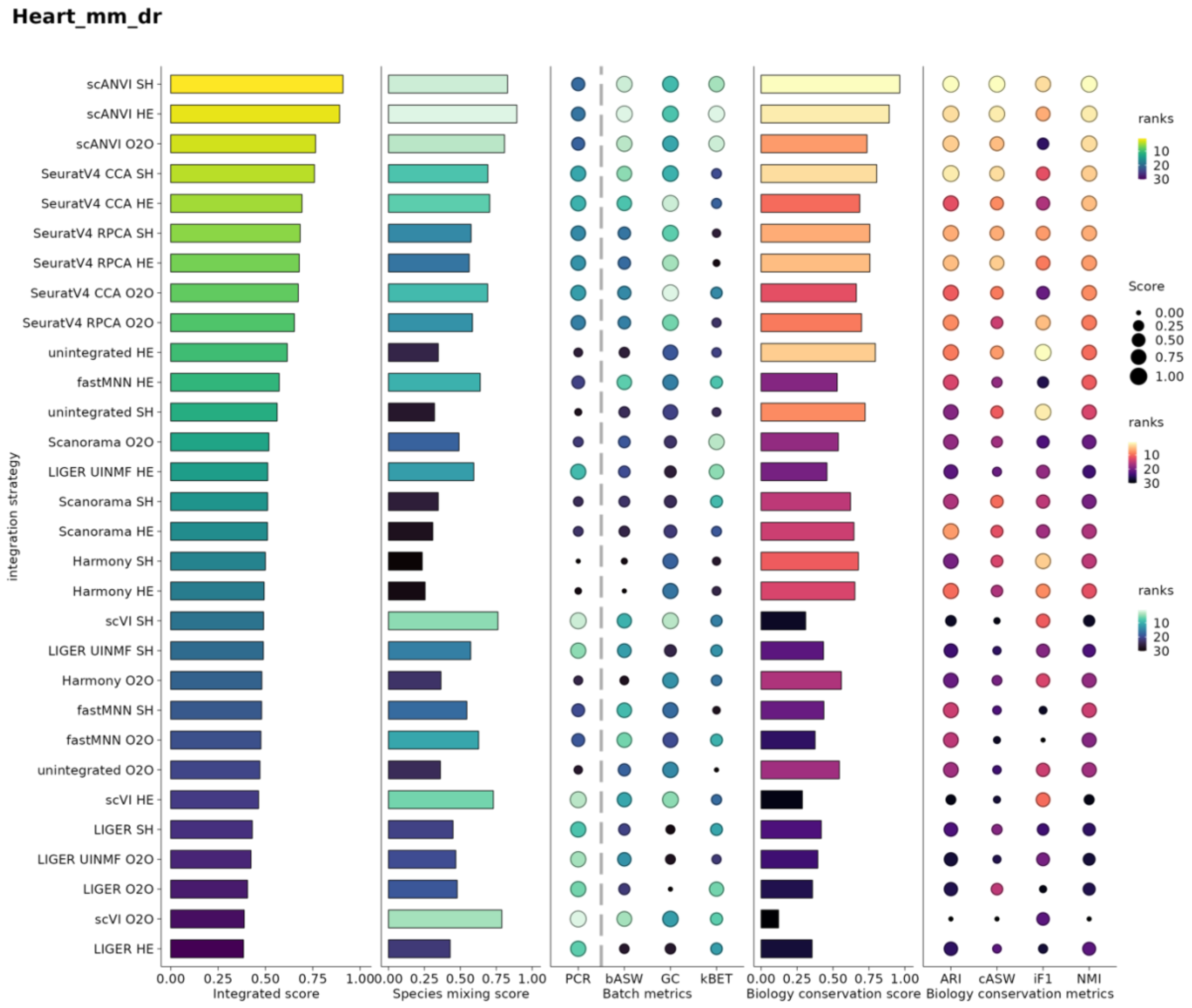

Supplementary Figure 15 Benchmarking scores and metrics of different integration strategies on cross-species analysis in the Heart_mm_dr task. Batch removal metrics and biological conservation metrics are min-max scaled per task. The species mixing score is the average of 4 batch removal metrics, while the biological conservation score is the average of 5 biology conservation metrics. The integrated score is a weighted average of species mixing score and biology conservation

score with 40/60 weighting. Grey dash lines indicate a distinction between metrics that do not rely on cell type annotation (PCR) with metrics relying on cell type annotation. O2O, only uses one-to-one orthologs; HE, one-to-one orthologs plus one-to-many and many-to-many orthologs matched by higher average expression level; SH, one-to-one orthologs plus one-to-many and many-to-many orthologs matched by stronger homology confidence; PCR: principal component regression; bASW: batch average silhouette width, GC: graph connectivity, kBET: k-nearest neighbour batch effect test, ARI: adjusted rand index, cASW: cell type average silhouette width, NMI: normalised mutual information of cell label, iF1: isolated label F1 score.

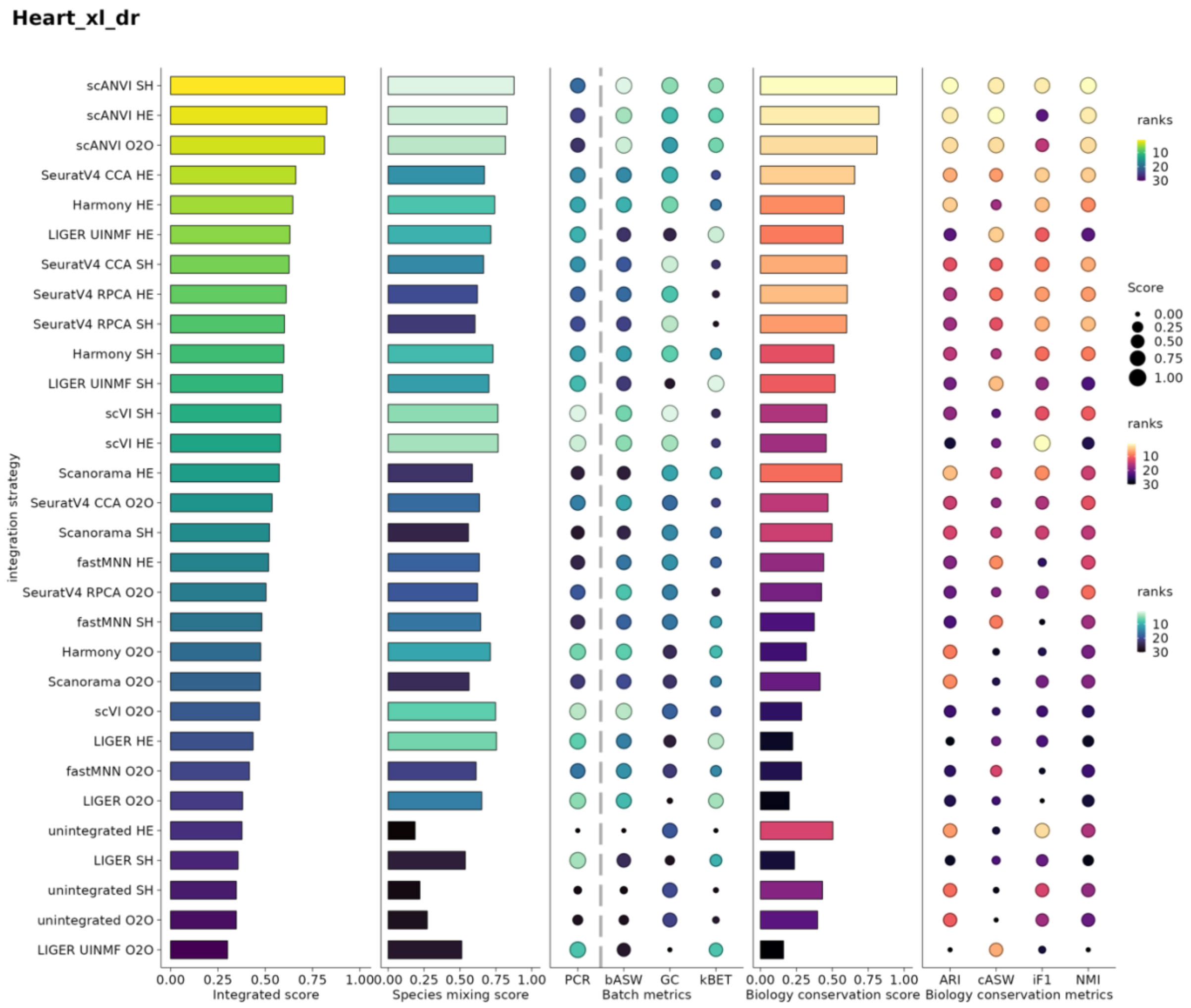

Supplementary Figure 16 Benchmarking scores and metrics of different integration strategies on cross-species analysis in the Heart_xl_dr task. Batch removal metrics and biological conservation metrics are min-max scaled per task. The species mixing score is the average of 4 batch

removal metrics, while the biological conservation score is the average of 5 biology conservation metrics. The integrated score is a weighted average of species mixing score and biology conservation score with 40/60 weighting. Grey dash lines indicate a distinction between metrics that do not rely on cell type annotation (PCR) with metrics relying on cell type annotation. O2O, only uses one-to-one orthologs; HE, one-to-one orthologs plus one-to-many and many-to-many orthologs matched by higher average expression level; SH, one-to-one orthologs plus one-to-many and many-to-many orthologs matched by stronger homology confidence; PCR: principal component regression; bASW: batch average silhouette width, GC: graph connectivity, kBET: k-nearest neighbour batch effect test, ARI: adjusted rand index, cASW: cell type average silhouette width, NMI: normalised mutual information of cell label, iF1: isolated label F1 score.

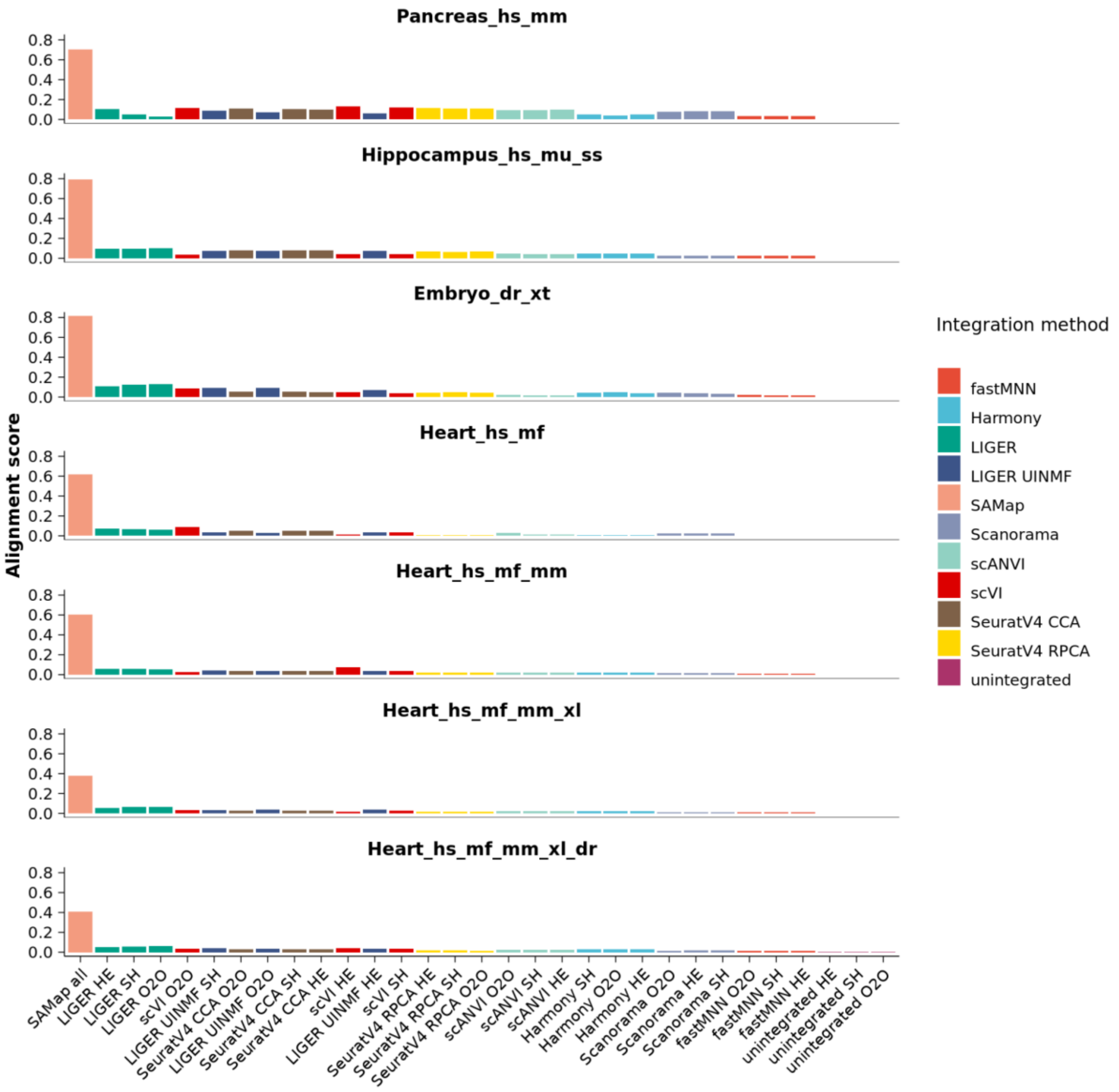

Supplementary Figure 17 Alignment score for all strategies in 7 reference integration tasks. Bar plot showing the alignment score for each strategy in each task. The score is calculated with the average number of cross-species neighbours as a percentage of the maximum number of neighbours (k=20 is used in this study, see Methods for score details). O2O, only uses one-to-one orthologs; HE, one-to-one orthologs plus one-to-many and many-to-many orthologs matched by higher average expression level; SH, one-to-one orthologs plus one-to-many and many-to-many orthologs matched by stronger homology confidence.

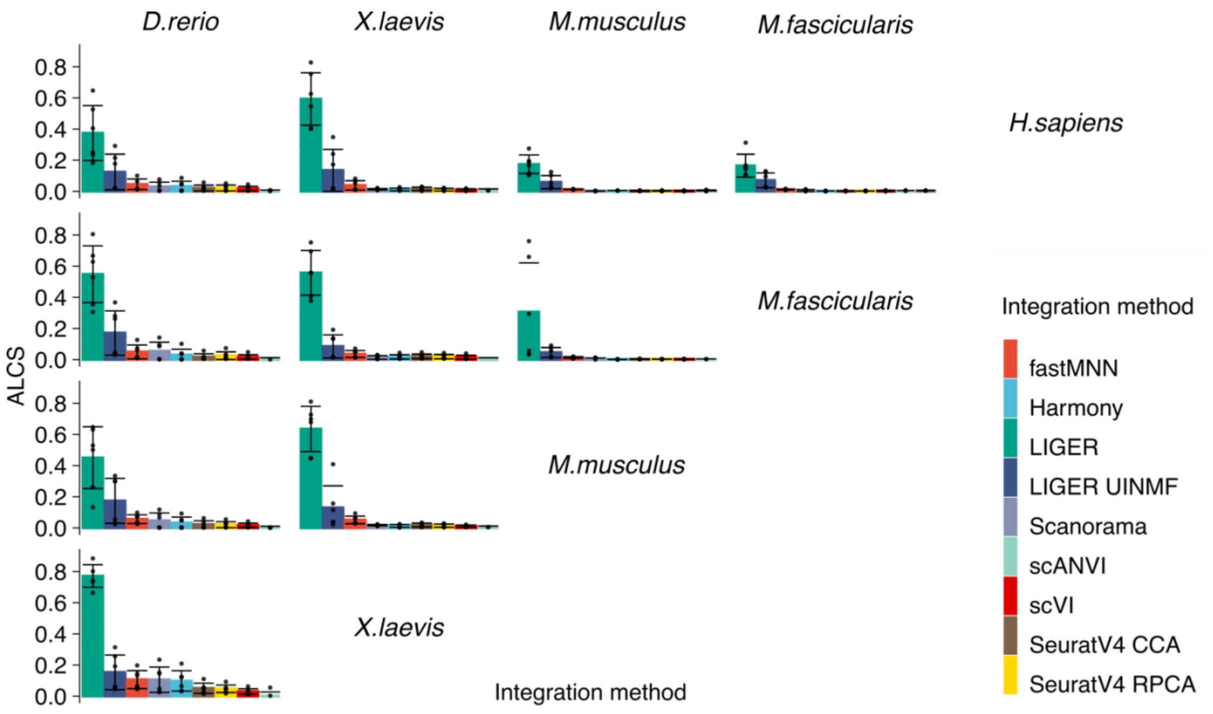

Supplementary Figure 18 ALCS for all integration strategies in pairwise integration of the heart task. Bars represent average ALCS across n=2 involved species and n=3 homology strategies for each integration method per integration task. Error bars indicate standard deviation. Individual data points are shown as black dots. ALCS: accuracy loss of cell type self-projection.

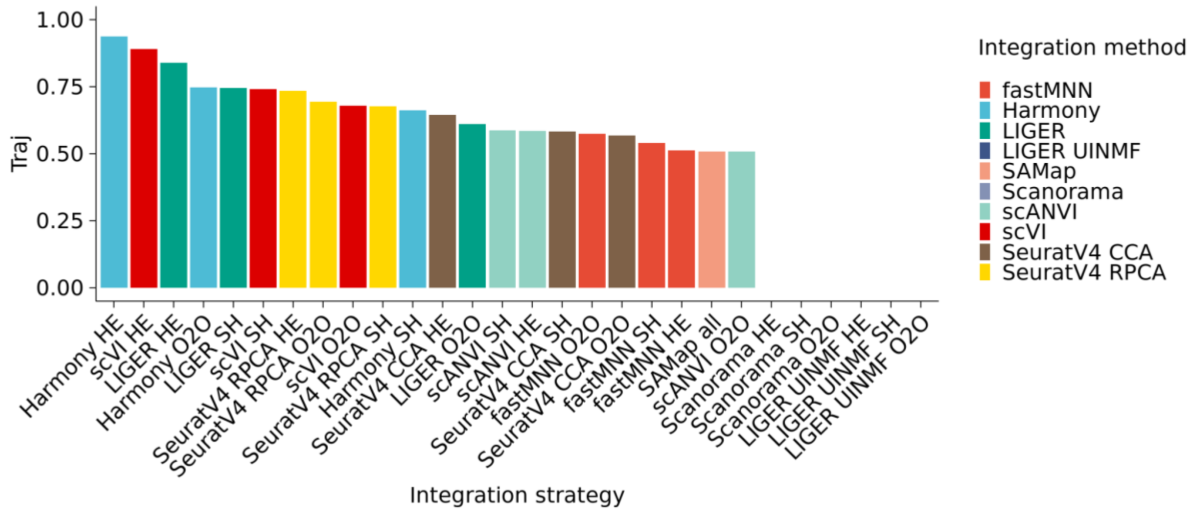

Supplementary Figure 19 Trajectory conservation score in the Embryo_dr_xt task of all strategies. Bars showing the single Traj score of each integration strategy. Calculation of Traj score is shown in Supplementary Methods. Traj, trajectory conservation score; O2O, only use one-to-one orthologs; HE, one-to-one orthologs plus one-to-many and many-to-many orthologs matched by higher average expression level; SH, one-to-one orthologs plus one-to-many and many-to-many orthologs matched by stronger homology confidence.

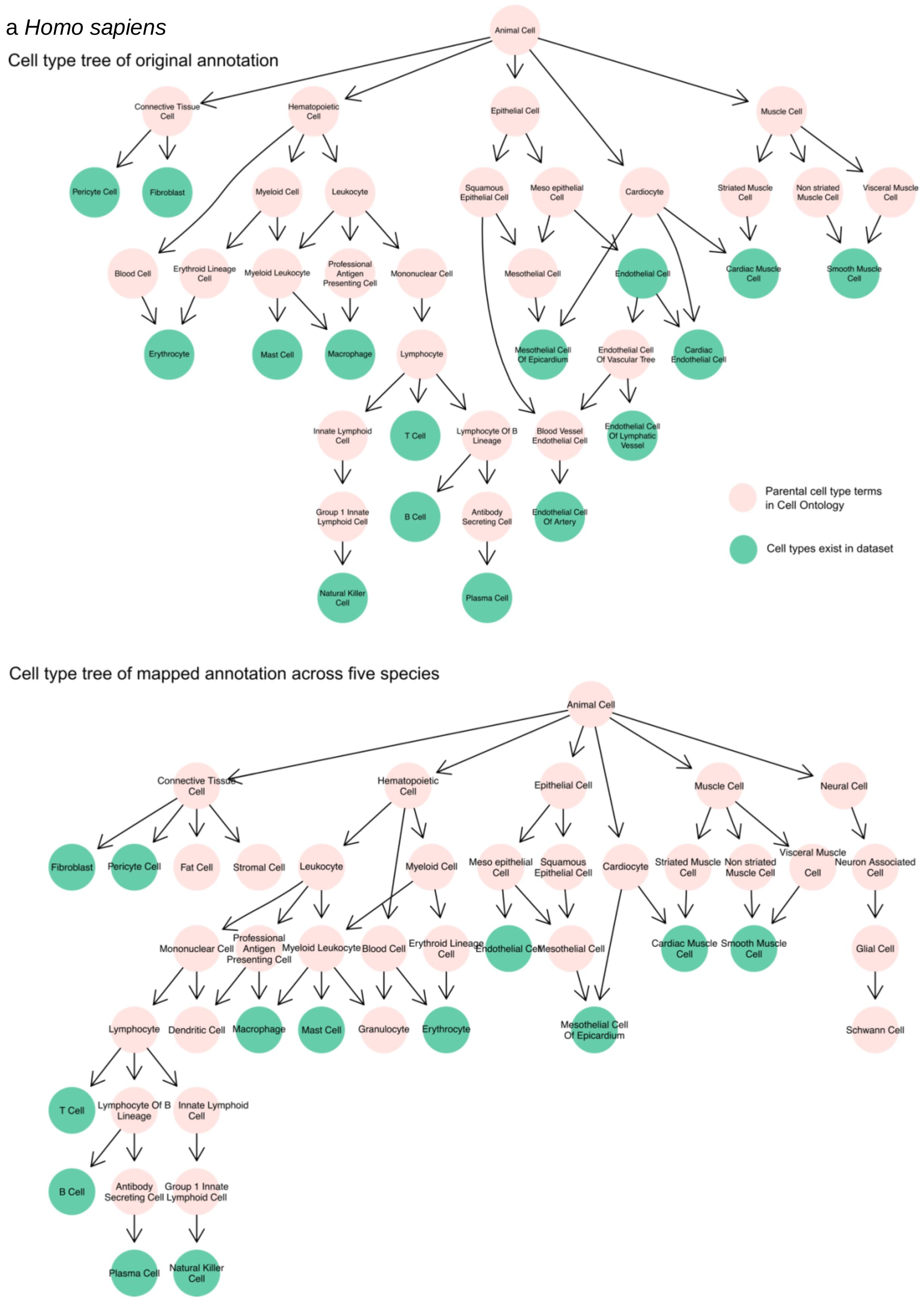
a Homo sapiens
Cell type tree of original annotation
Animal Cell
Connective Tissue Cell
Hematopoietic Cell
Epithelial Cell
Muscle Cell
Pericyte Cell
Fibroblast
Myeloid Cell
Leukocyte
Squamous Epithelial Cell
Meso epithelial Cell
Cardiocyte
Striated Muscle Cell
Non striated Muscle Cell
Visceral Muscle Cell
Blood Cell
Erythroid Lineage Cell
Myeloid Leukocyte
Professional Antigen Presenting Cell
Mononuclear Cell
Mesothelial Cell
Endothelial Cell
Cardiac Muscle Cell
Smooth Muscle Cell
Erythrocyte
Mast Cell
Macrophage
Lymphocyte
Mesothelial Cell Of Epicardium
Endothelial Cell Of Vascular Tree
Cardiac Endothelial Cell
Innate Lymphoid Cell
T Cell
Lymphocyte Of B Lineage
Blood Vessel Endothelial Cell
Endothelial Cell Of Lymphatic Vessel
Group 1 Innate Lymphoid Cell
B Cell
Antibody Secreting Cell
Endothelial Cell Of Artery
Natural Killer Cell
Plasma Cell
Parental cell type terms in Cell Ontology
Cell types exist in dataset
Cell type tree of mapped annotation across five species
Animal Cell
Connective Tissue Cell
Hematopoietic Cell
Epithelial Cell
Muscle Cell
Neural Cell
Fibroblast
Pericyte Cell
Fat Cell
Stromal Cell
Leukocyte
Myeloid Cell
Meso epithelial Cell
Squamous Epithelial Cell
Cardiocyte
Striated Muscle Cell
Non striated Muscle Cell
Visceral Muscle Cell
Neuron Associated Cell
Mononuclear Cell
Professional Antigen Presenting Cell
Myeloid Leukocyte
Blood Cell
Erythroid Lineage Cell
Endothelial Cell
Mesothelial Cell
Cardiac Muscle Cell
Smooth Muscle Cell
Glial Cell
Lymphocyte
Dendritic Cell
Macrophage
Mast Cell
Granulocyte
Erythrocyte
Mesothelial Cell Of Epicardium
Schwann Cell
T Cell
Lymphocyte Of B Lineage
Innate Lymphoid Cell
B Cell
Antibody Secreting Cell
Group 1 Innate Lymphoid Cell
Plasma Cell
Natural Killer Cell

b *Macaca fascicularis*

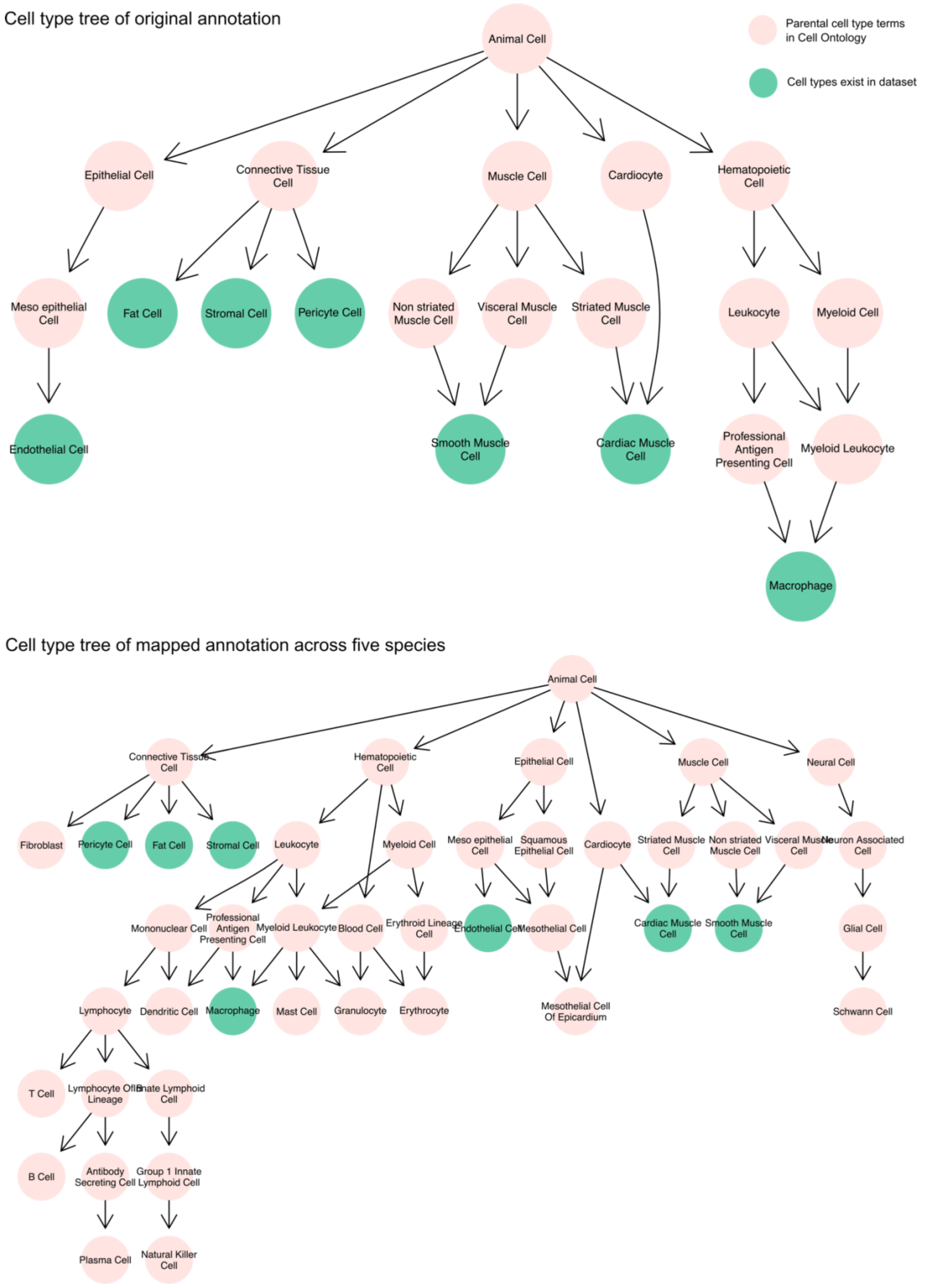

c *Mus musculus*

Cell type tree of original annotation

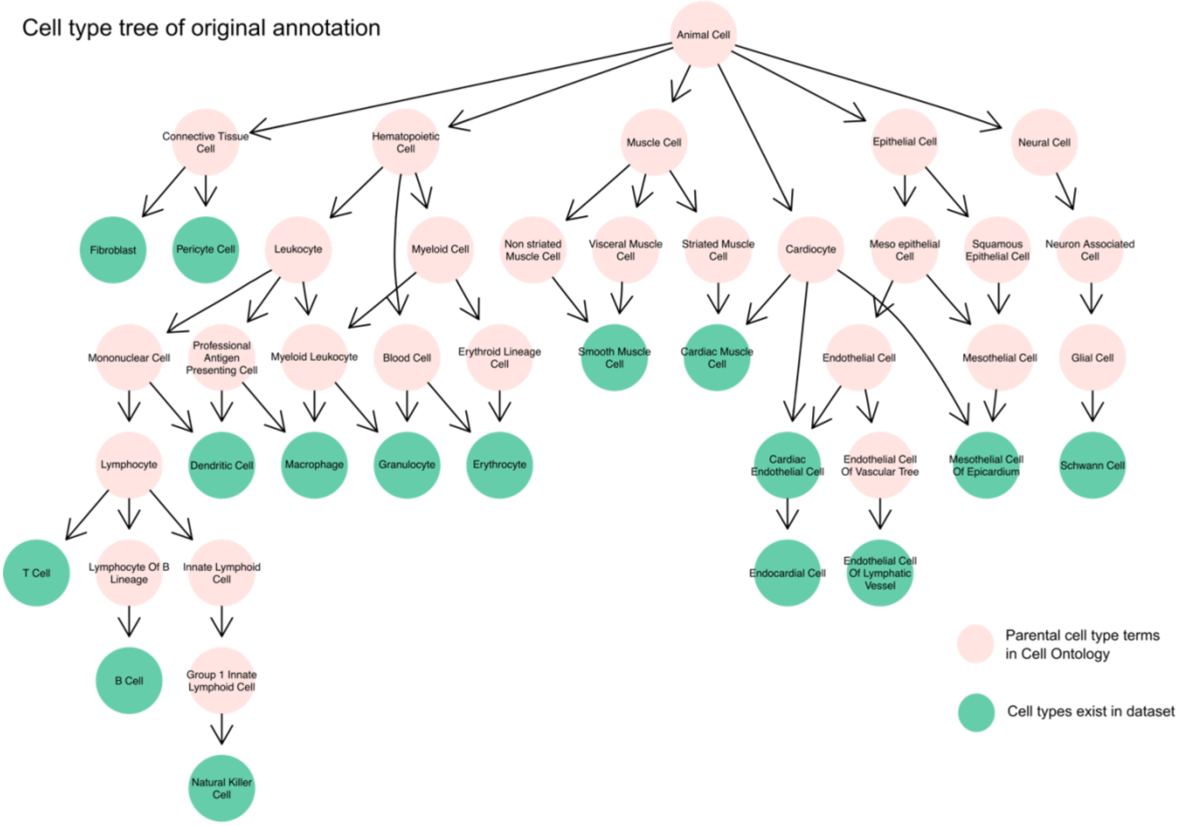

Cell type tree of mapped annotation across five species

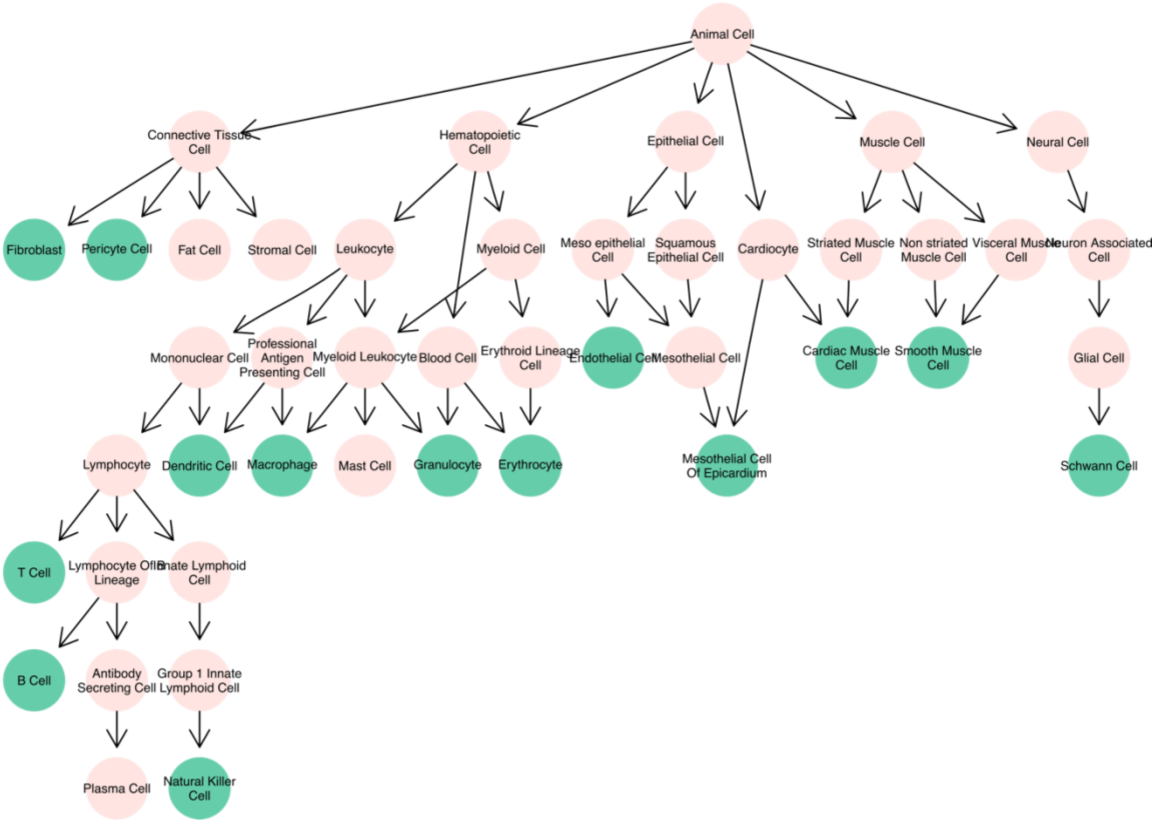

d *Xenopus laevis*

Cell type tree of original annotation

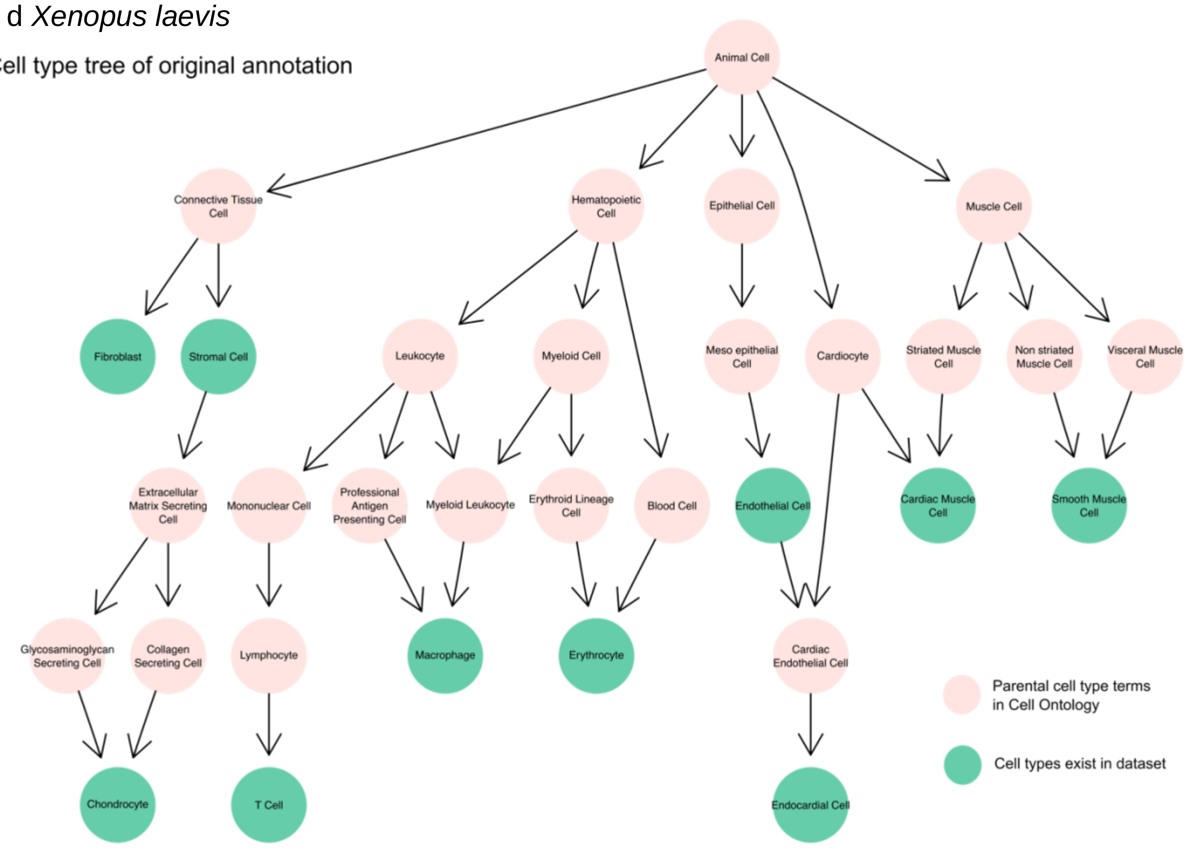

Cell type tree of mapped annotation across five species

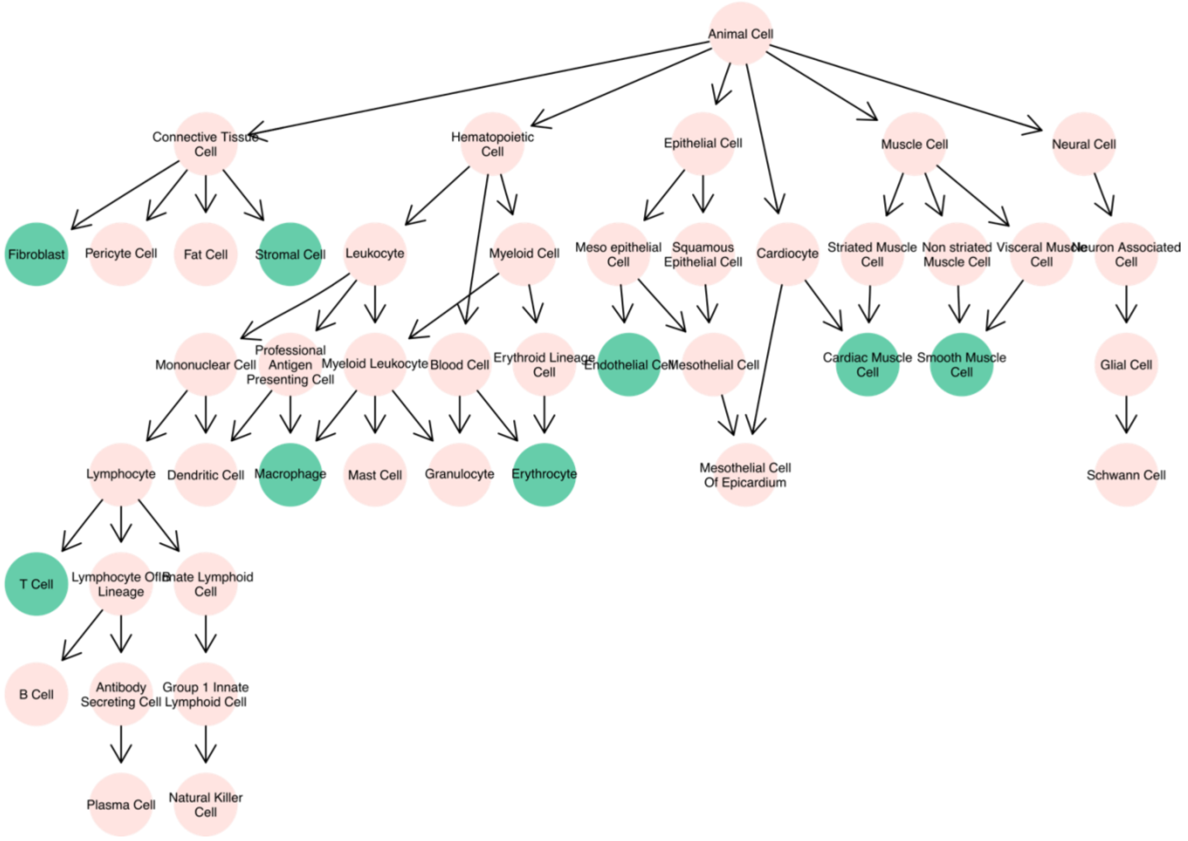

e *Danio rerio*

Cell type tree of original annotation

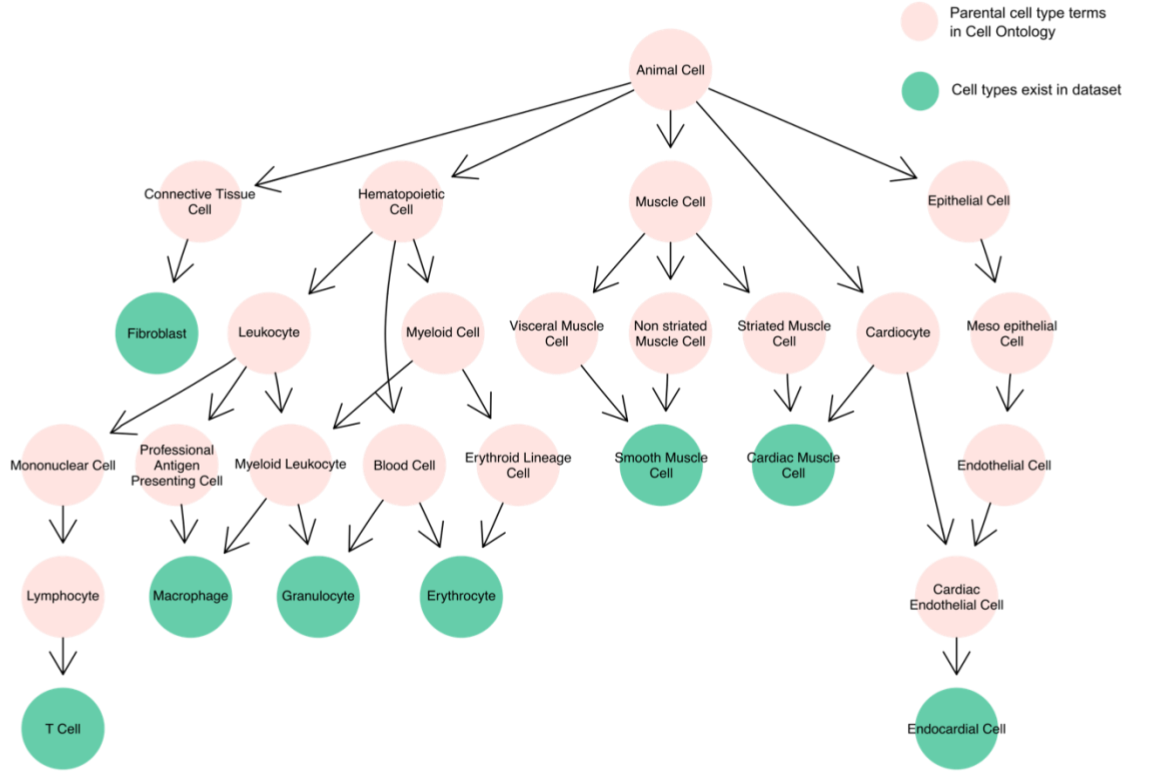

Cell type tree of mapped annotation across five species

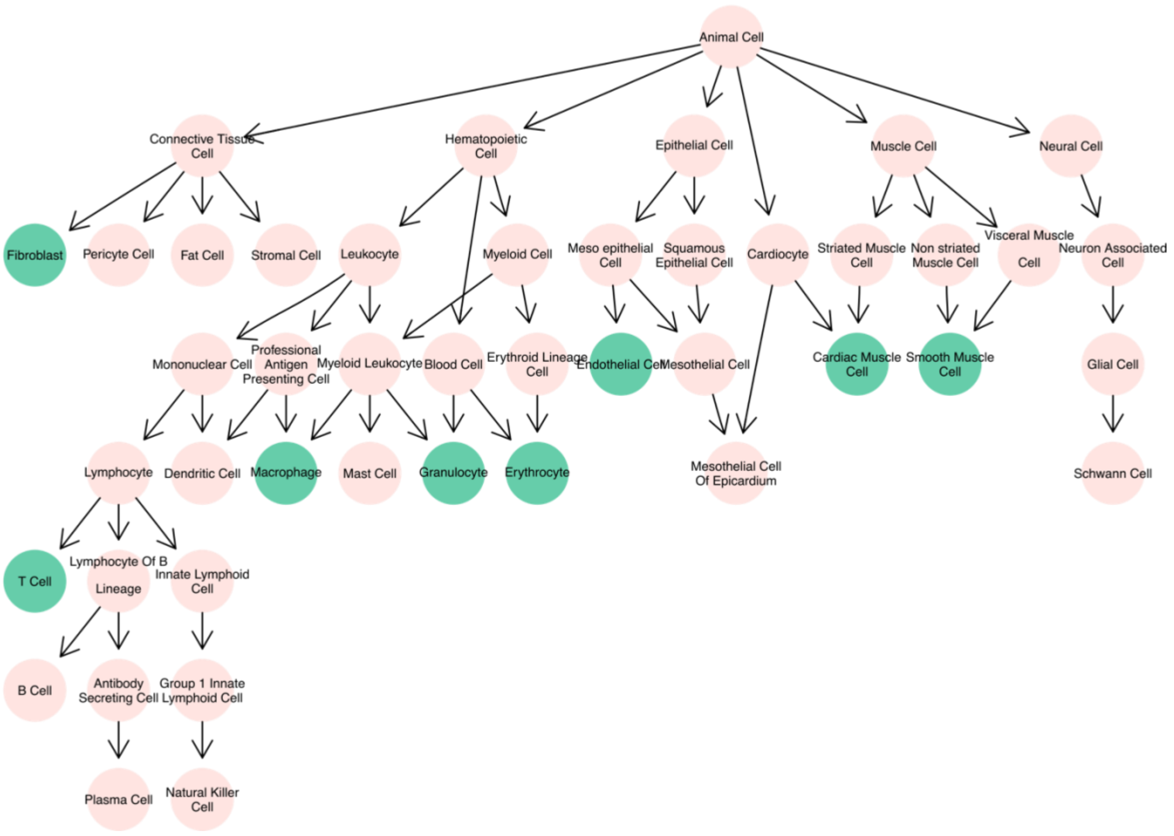

Supplementary Figure 20 Aligned cell type tree in heart task among five species with scOntoMatch. Annotated cell types from the heart tissue of the five species are represented as cell type trees whose hierarchy follows Cell Ontology [1]. Each circle represents a cell type term in the Cell Ontology system and is coloured by whether the term is present in the dataset. Each arrow starts from a parental term and ends with its children term. scOntoMatch is used to align the ontology annotation across the five species, including (a) *Homo sapiens*, (b) *Macaca fascicularis,* (c) *Mus musculus,* (d) *Xenopus laevis* and (e) *Danio rerio*, resulting in comparable annotation granularity. The cell type tree of the original annotation is shown in the top half and the bottom half shows scOntoMatch aligned cell type annotation for each species.

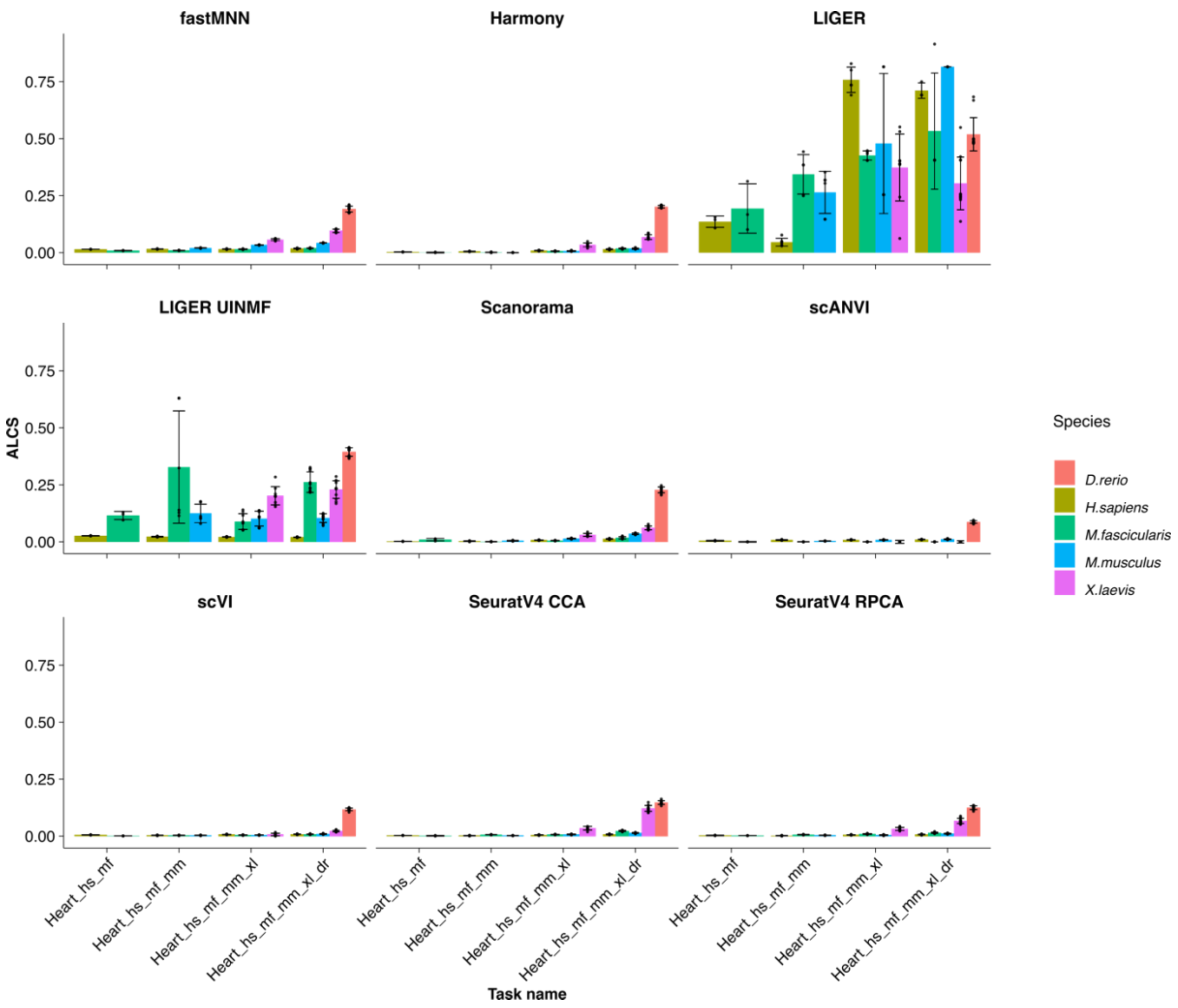

Supplementary Figure 21 Per-species ALCS in all strategies in heart tasks. Bars represent the mean ALCS of all homology methods and error bars indicate standard deviation. Individual data points are shown as black dots. Lower ALCS suggests less loss of cell type distinguishability after integration. ALCS: accuracy loss of cell type self-projection.

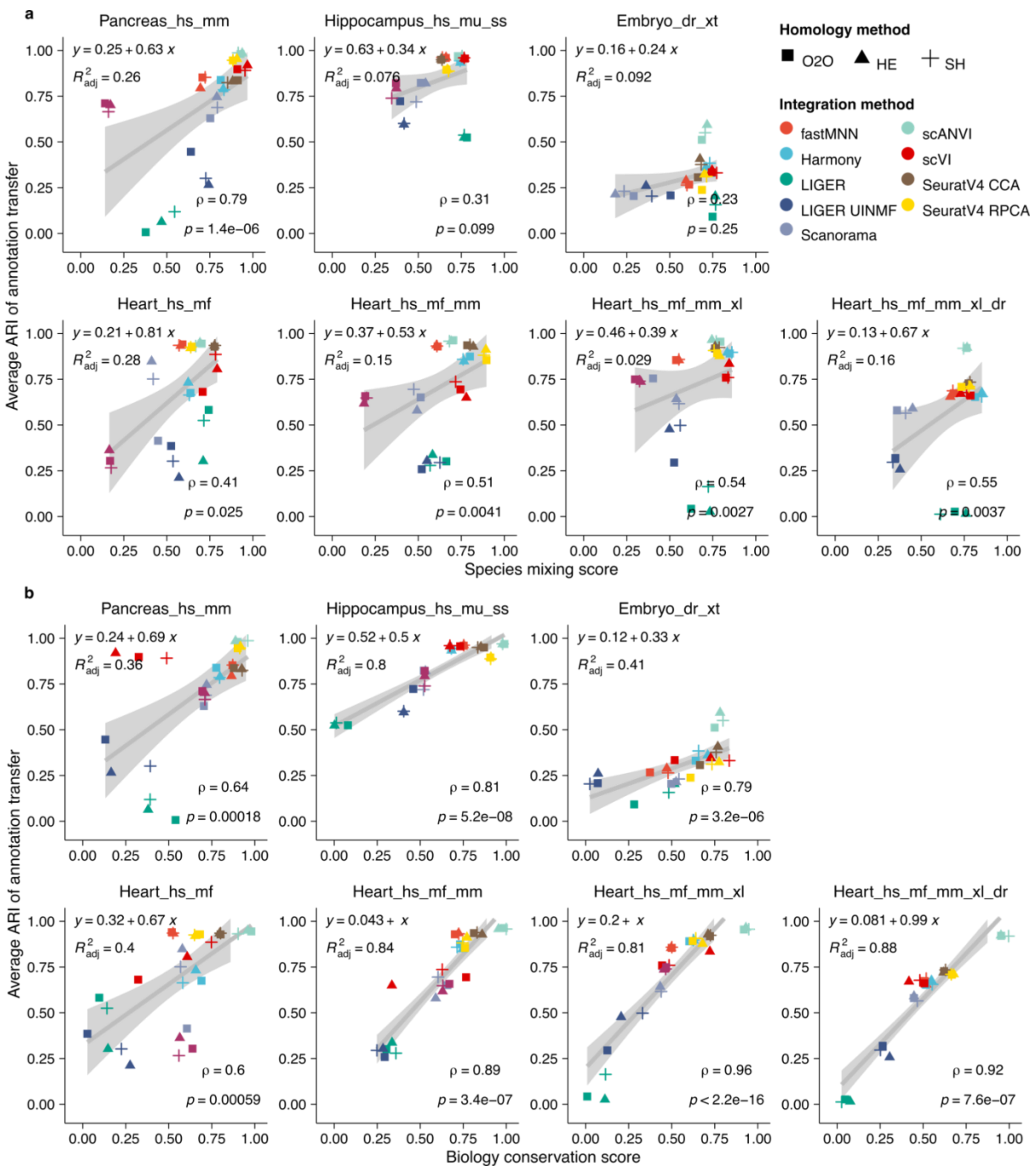

Supplementary Figure 22 Correlation between ARI and species mixing score or biology conservation score for 7 reference tasks in all strategies. The average ARIs between transferred annotation and original annotation in 7 reference tasks are shown for all integration strategies. A high ARI suggests a successful annotation transfer. Spearman's rank correlation coefficients indicate that ARI significantly positively correlates with biology conservation score in all tasks (a, two-sided Student's t-test P value < 0.001) but not always for species mixing score (b). This is further

demonstrated by a linear regression using y=x indicated by the grey line and the band shows 95% confidence interval. Strategies are represented by points whose colour is the integration algorithm and whose shape is the homology method. ARI, adjusted rand index; ρ, Spearman's rank correlation coefficient; p, P-value of Spearman's rank correlation; R^2_{adj}, adjusted goodness-of-fit of the linear model; O2O, only use one-to-one orthologs; HE, one-to-one orthologs plus one-to-many and many-to-many orthologs matched by higher average expression level; SH, one-to-one orthologs plus one-to-many and many-to-many orthologs matched by stronger homology confidence.

a

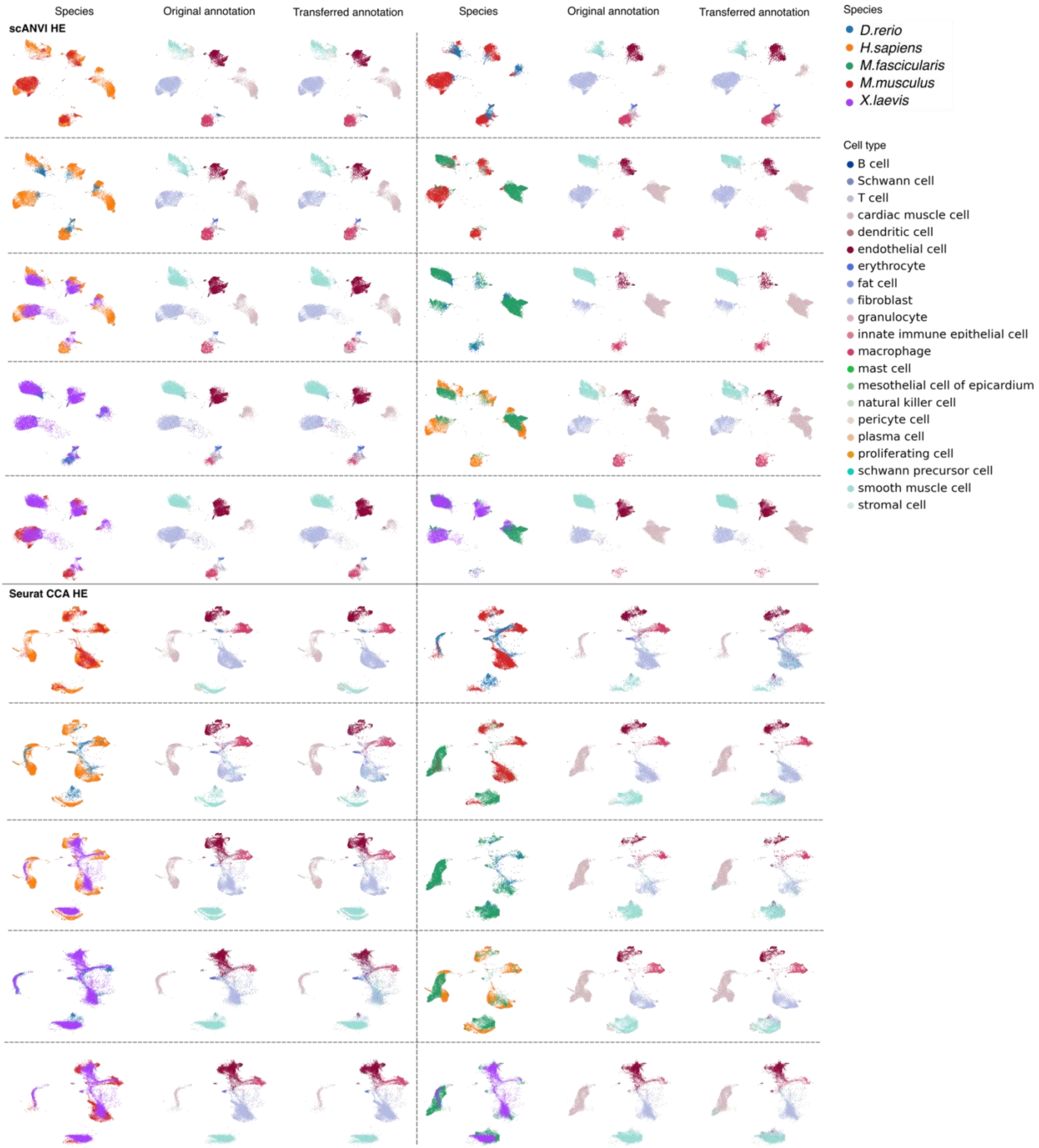
Species
Original annotation
Transferred annotation
scANVI HE
Seurat CCA HE
Species
D.rerio
H.sapiens
M.fascicularis
M.musculus
X.laevis
Cell type
B cell
Schwann cell
T cell
cardiac muscle cell
dendritic cell
endothelial cell
erythrocyte
fat cell
fibroblast
granulocyte
innate immune epithelial cell
macrophage
mast cell
mesothelial cell of epicardium
natural killer cell
pericyte cell
plasma cell
proliferating cell
schwann precursor cell
smooth muscle cell
stromal cell

b

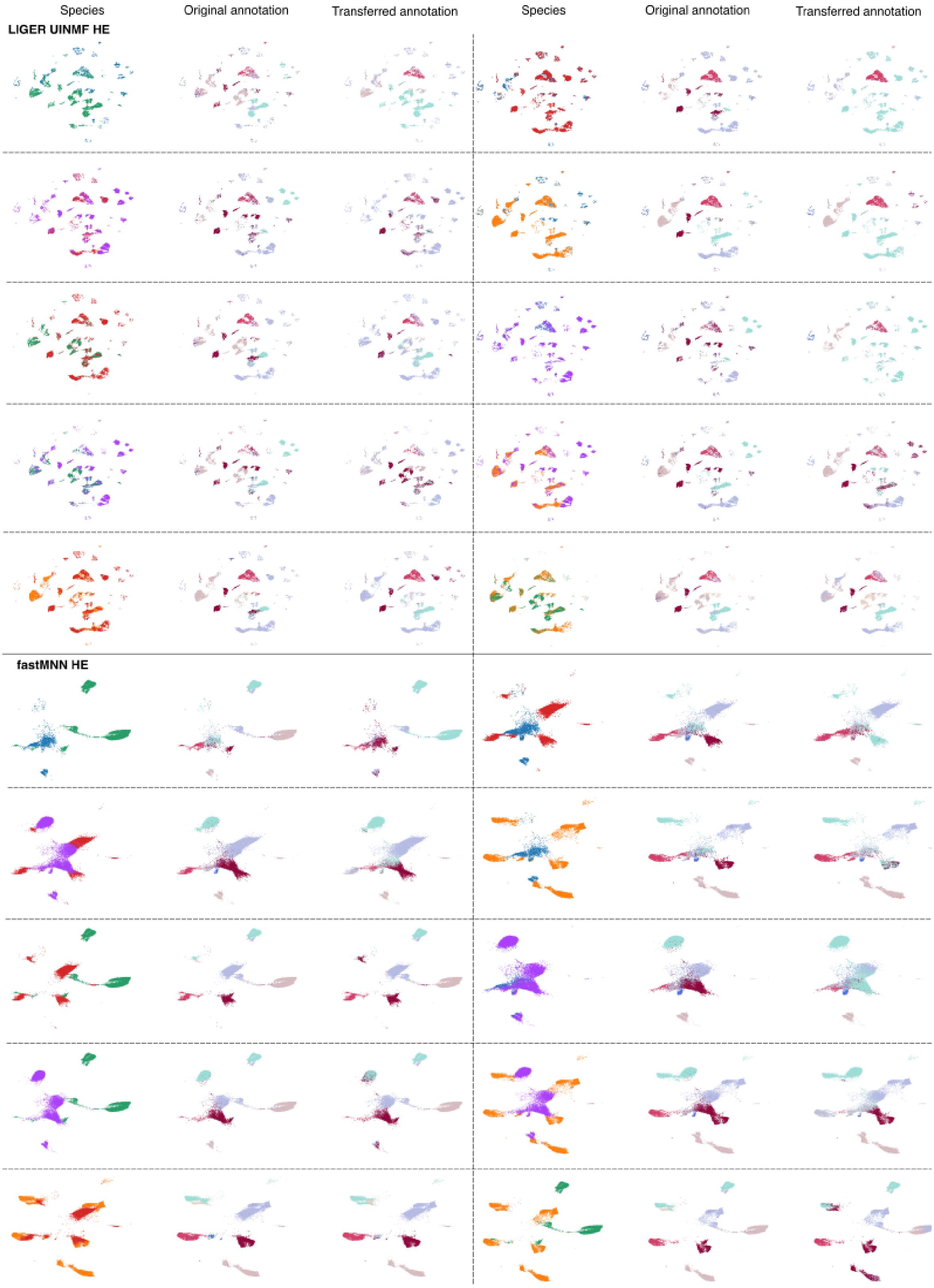
Species
Original annotation
Transferred annotation
Species
Original annotation
Transferred annotation
LIGER UINMF HE
fastMNN HE

Supplementary Figure 23 UMAP visualisation of species, original and transferred annotations in the Heart_hs_mf_mm_xl_dr task. Showing UMAPs from (a) scANVI HE and SeuratV4 CCA HE as examples of a successful annotation transfer. Despite minor confusions between similar cell types, such as epithelial cells and smooth muscle cells, annotation transfer is to a large extent successful even between distant species such as human and zebrafish in these two strategies. On the other hand, results from (b) LIGER UINMF HE and fastMNN HE were unsuccessful as transferred annotation diverged greatly from the original annotation. UMAP, Uniform Manifold Approximation and Projection, HE, one-to-one orthologs plus one-to-many and many-to-many orthologs matched by higher average expression level.

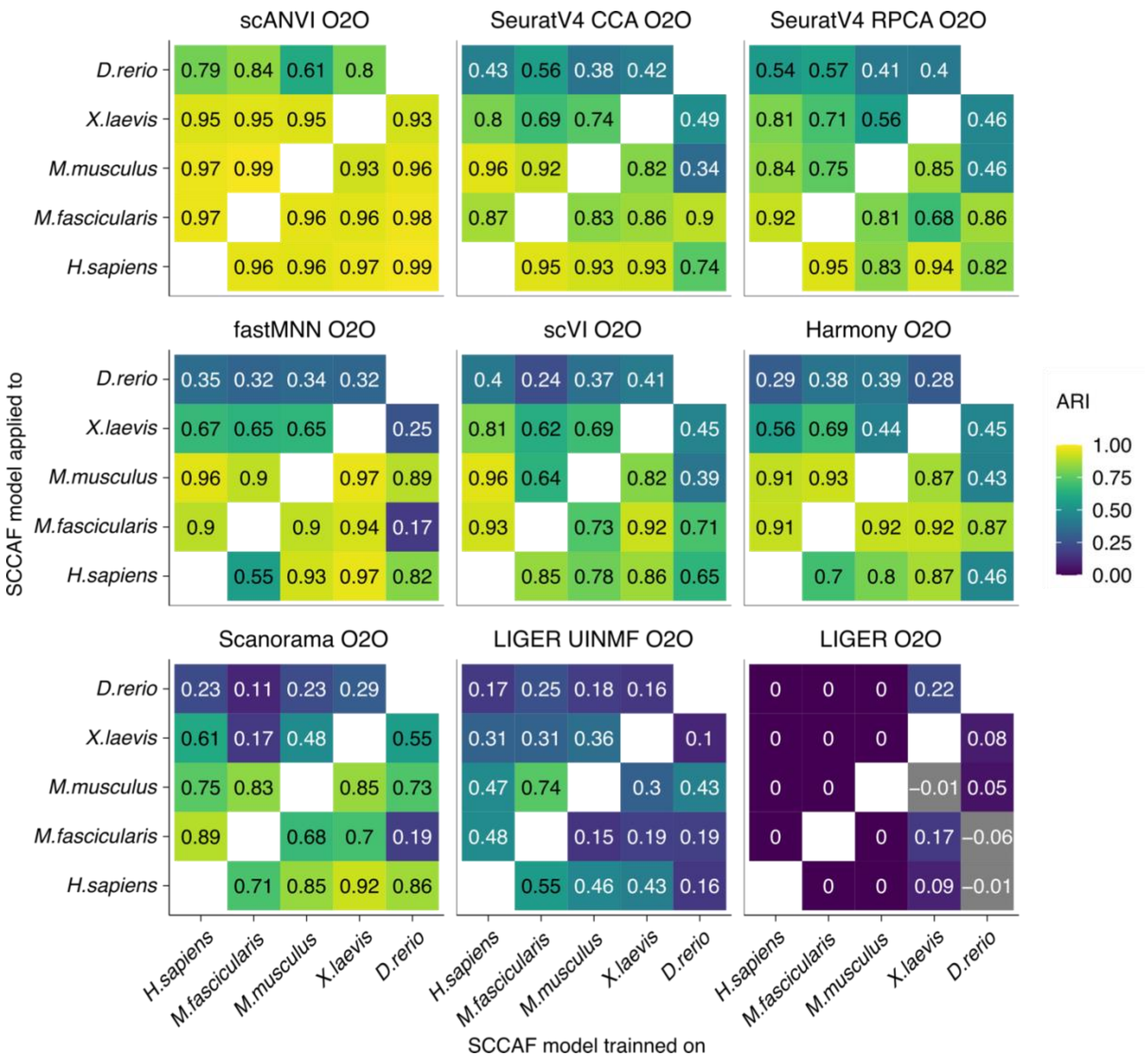

Supplementary Figure 24 ARI for annotation transfer in one-to-one orthologs integrated data in the Heart_hs_mf_mm_xl_dr task between pairs of species. Showing the pairwise cell type annotation transfer ARI between all pairs of species. Generally, species that have diverged for a long time have lower ARI, suggesting a more challenging annotation transfer. O2O, only uses one-to-one orthologs; SCCAF, single cell clustering assessment framework; ARI, adjusted rand index.

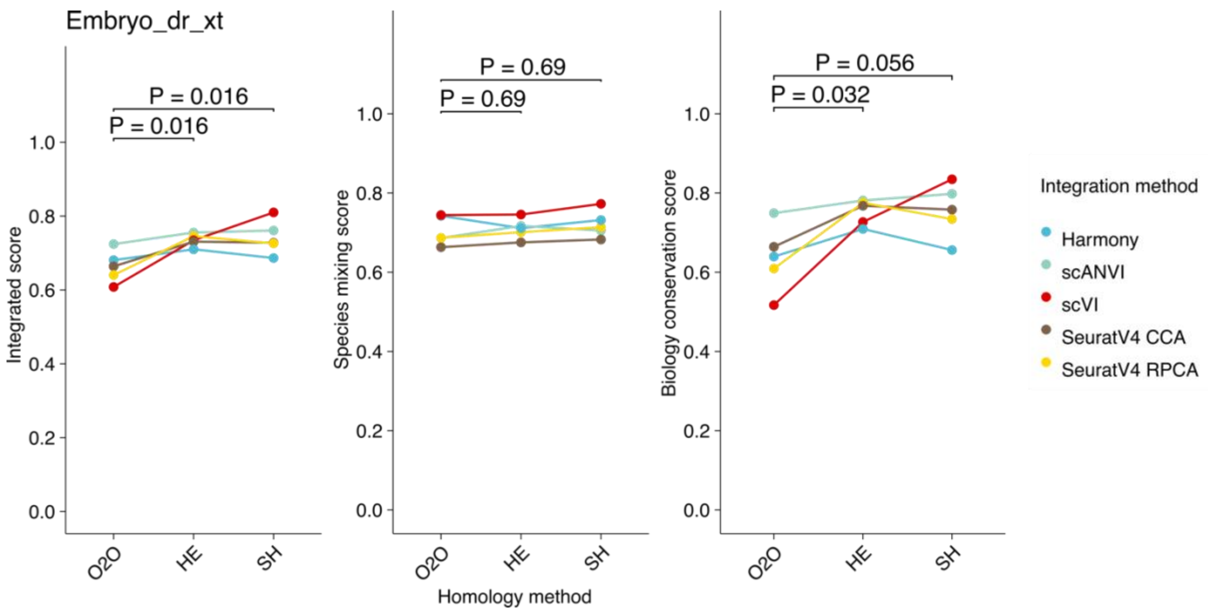

Supplementary Figure 25 Improvement of scores by adding in-paralogs in the Embryo_dr_xt task for successful algorithms. There were 5 algorithms that achieved higher integrated scores than unintegrated data and were considered successful. We compare the benchmarking scores between O2O and HE, or O2O and SH to investigate the improvement by including in-paralogs. Including HE in-paralogs improved biology conservation score. P values are from Wilcoxon signed rank tests (two-sided) adjusted by the Benjamin-Hochberg procedure. Adjusted P value < 0.05 is considered statistically significant. O2O, only use one-to-one orthologs; HE, one-to-one orthologs plus one-to-many and many-to-many orthologs matched by higher average expression level; SH, one-to-one orthologs plus one-to-many and many-to-many orthologs matched by stronger homology confidence.

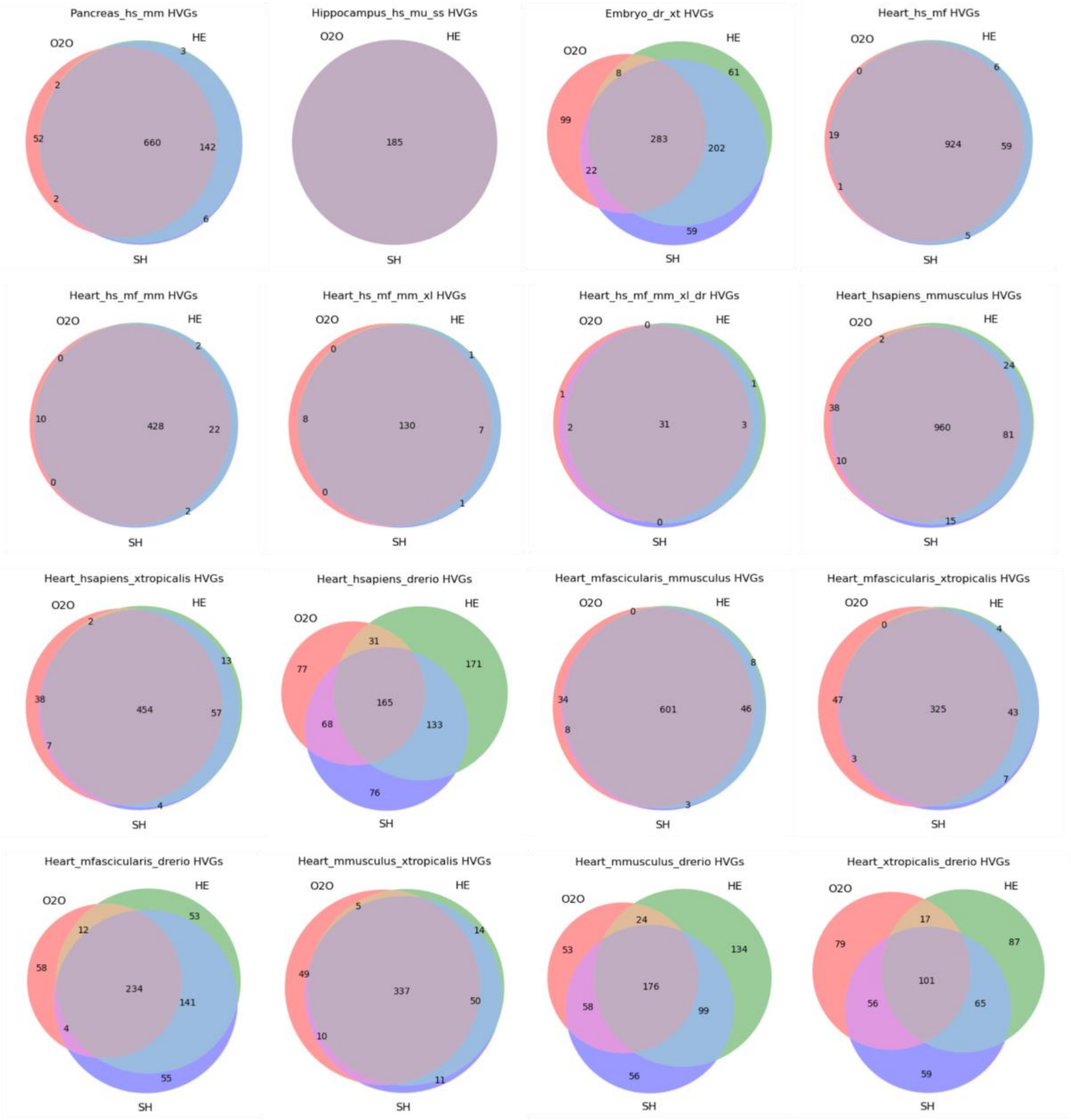

Supplementary Figure 26 Intersection of HVGs between three types of homology concatenated data in 16 tasks. HVGs are selected per batch key (see Methods for the batch key of each task). Venn diagrams show the intersection of HVGs among three types of homology concatenated data. Species that have diverged for a longer time share less HVGs among different homology methods. HVG, highly variable genes; O2O, only use one-to-one orthologs; HE, one-to-one orthologs plus one-to-many and many-to-many orthologs matched by higher average expression level; SH, one-to-one orthologs plus one-to-many and many-to-many orthologs matched by stronger homology confidence.

Pancreas_hs_mm task

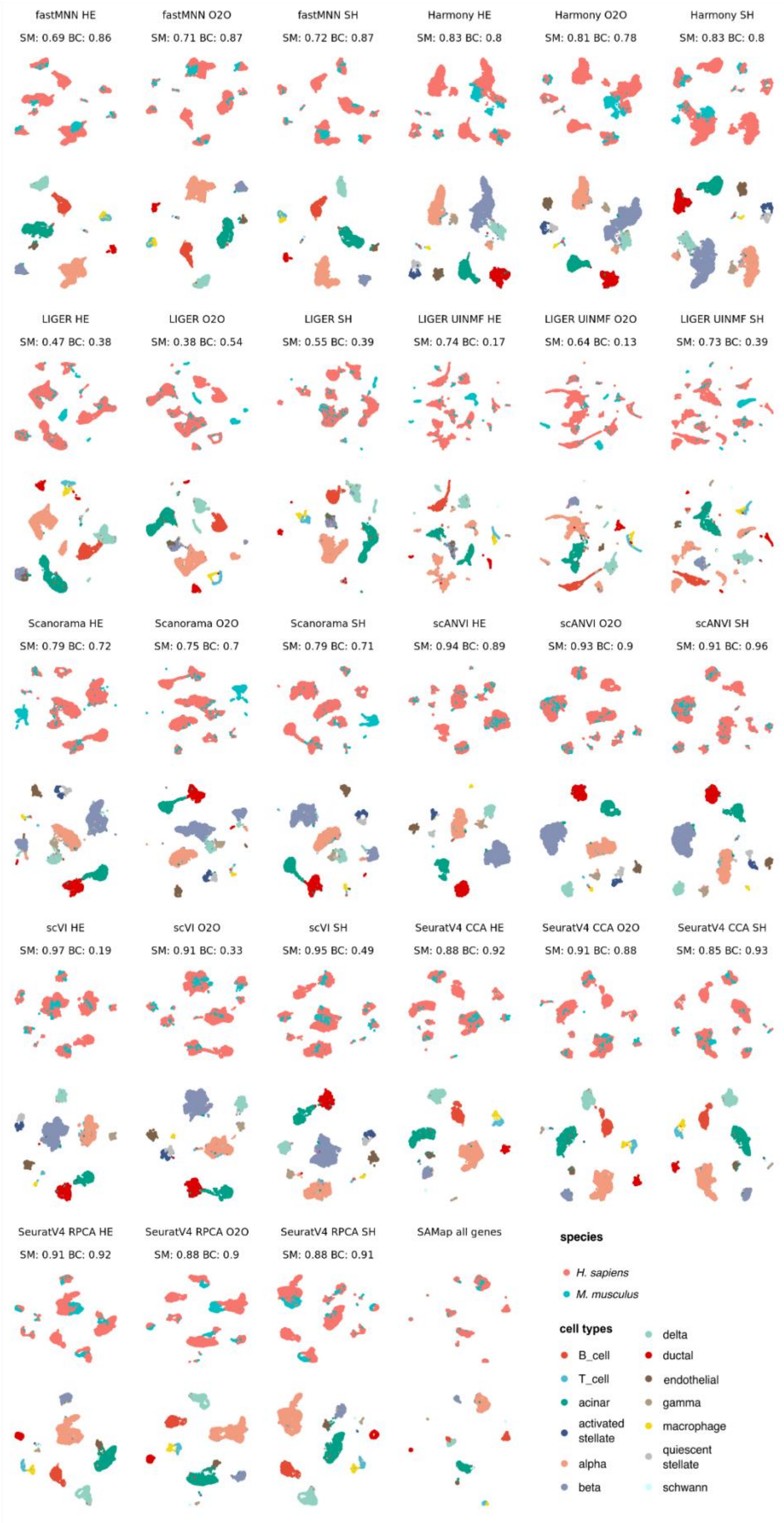

Supplementary Figure 27 UMAP visualisation of integration results from 28 strategies in Pancreas_hs_mm task. Larger images available at the BENGAL reproducibility repository on GitHub [2]. UMAP, Uniform Manifold Approximation and Projection, SM, species mixing score, BC, biology conservation score, O2O, only uses one-to-one orthologs, HE, one-to-one orthologs plus one-to-many and many-to-many orthologs matched by higher average expression level, SH, one-to-one orthologs plus one-to-many and many-to-many orthologs matched by stronger homology confidence.

Hippocampus_hs_mu_ss task

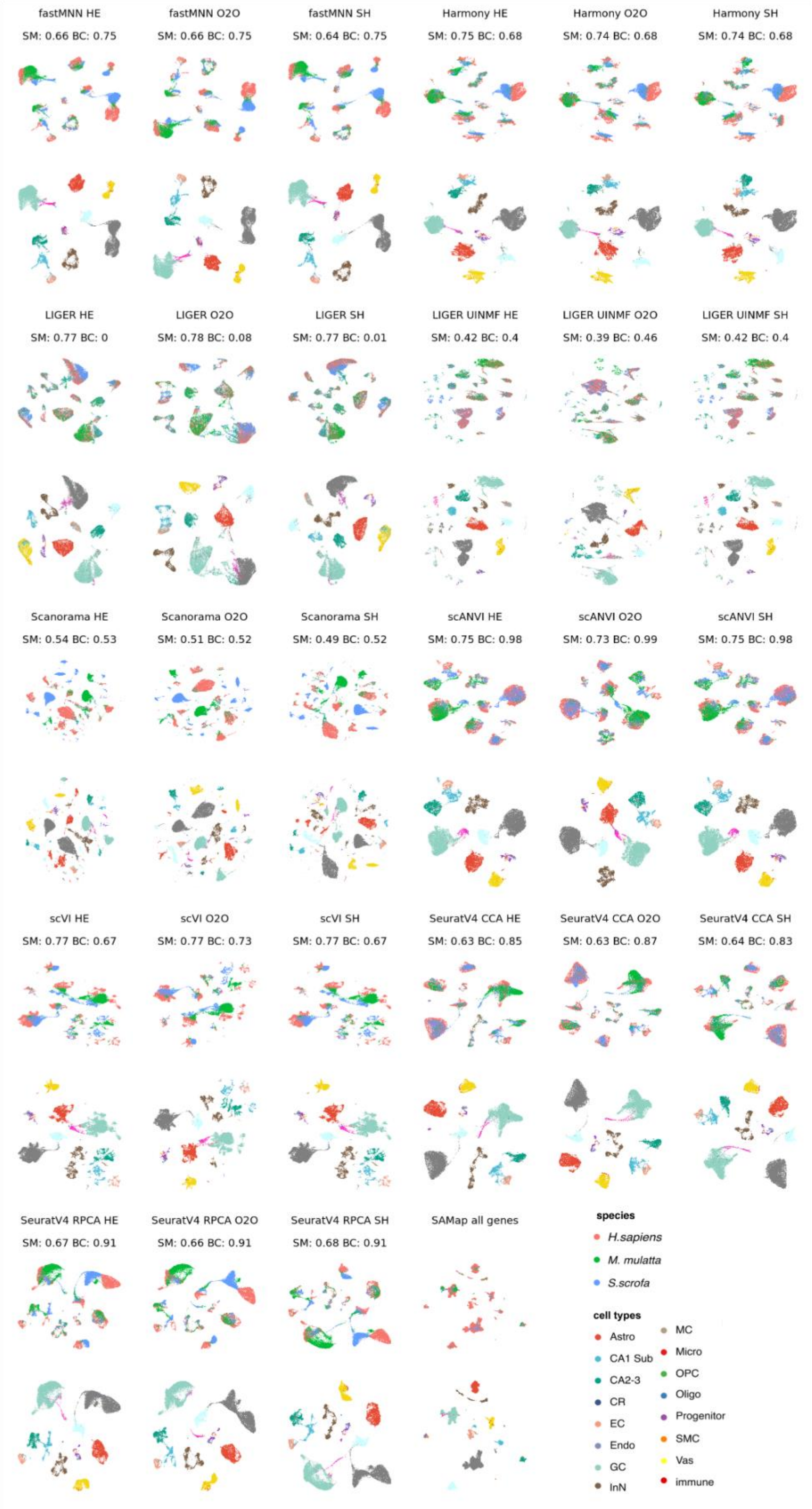

Supplementary Figure 28 UMAP visualisation of integration results from 28 strategies in Hippocampus_hs_mu_ss task. Larger images available at the BENGAL reproducibility repository on GitHub [2]. UMAP, Uniform Manifold Approximation and Projection, SM, species mixing score, BC, biology conservation score, O2O, only uses one-to-one orthologs, HE, one-to-one orthologs plus one-to-many and many-to-many orthologs matched by higher average expression level, SH, one-to-one orthologs plus one-to-many and many-to-many orthologs matched by stronger homology confidence.

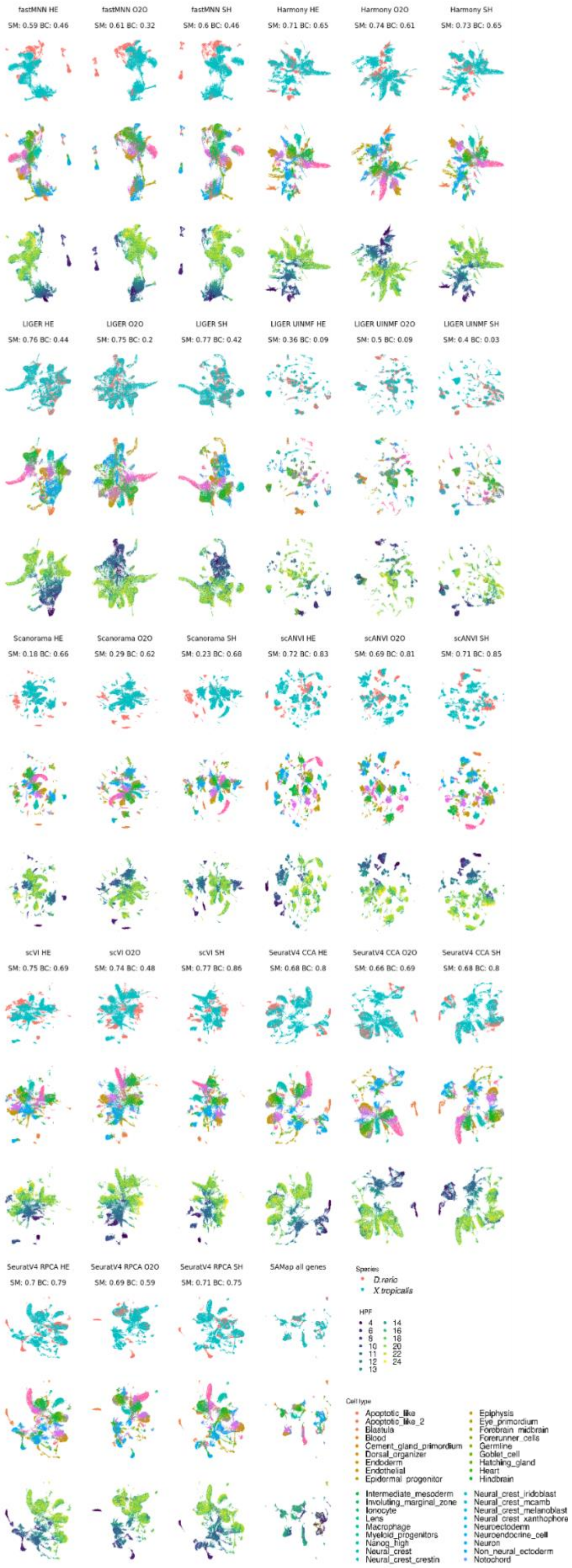
fastMNN HE
SM: 0.59 BC: 0.46
fastMNN O2O
SM: 0.61 BC: 0.32
fastMNN SH
SM: 0.6 BC: 0.46
Harmony HE
SM: 0.71 BC: 0.65
Harmony O2O
SM: 0.74 BC: 0.61
Harmony SH
SM: 0.73 BC: 0.65
LIGER HE
SM: 0.76 BC: 0.44
LIGER O2O
SM: 0.75 BC: 0.2
LIGER SH
SM: 0.77 BC: 0.42
LIGER UINMF HE
SM: 0.36 BC: 0.09
LIGER UINMF O2O
SM: 0.5 BC: 0.09
LIGER UINMF SH
SM: 0.4 BC: 0.03
Scanorama HE
SM: 0.18 BC: 0.66
Scanorama O2O
SM: 0.29 BC: 0.62
Scanorama SH
SM: 0.23 BC: 0.68
scANVI HE
SM: 0.72 BC: 0.83
scANVI O2O
SM: 0.69 BC: 0.81
scANVI SH
SM: 0.71 BC: 0.85
scVI HE
SM: 0.75 BC: 0.69
scVI O2O
SM: 0.74 BC: 0.48
scVI SH
SM: 0.77 BC: 0.86
SeuratV4 CCA HE
SM: 0.68 BC: 0.8
SeuratV4 CCA O2O
SM: 0.66 BC: 0.69
SeuratV4 CCA SH
SM: 0.68 BC: 0.8
SeuratV4 RPCA HE
SM: 0.7 BC: 0.79
SeuratV4 RPCA O2O
SM: 0.69 BC: 0.59
SeuratV4 RPCA SH
SM: 0.71 BC: 0.75
SAMap all genes
Species
D.rerio
X.tropicalis
HPF
4
6
8
10
11
12
13
14
16
18
20
22
24
Cell type
Apoptotic_like
Apoptotic_like_2
Blastula
Blood
Cement_gland_primordium
Dorsal_organizer
Endoderm
Endothelial
Epidermal_progenitor
Epiphysis
Eye_primordium
Forebrain_midbrain
Forerunner_cells
Germline
Goblet_cell
Hatching_gland
Heart
Hindbrain
Intermediate_mesoderm
Involuting_marginal_zone
Ionocyte
Lens
Macrophage
Myeloid_progenitors
Nanog_high
Neural_crest
Neural_crest_crestin
Neural_crest_iridoblast
Neural_crest_mcamb
Neural_crest_melanoblast
Neural_crest_xanthophore
Neuroectoderm
Neuroendocrine_cell
Neuron
Non_neural_ectoderm
Notochord

Supplementary Figure 29 UMAP visualisation of integration results from 28 strategies in Emvryo_dr_xt task. Larger images available at the BENGAL reproducibility repository on GitHub [2]. UMAP, Uniform Manifold Approximation and Projection, SM, species mixing score, BC, biology conservation score, O2O, only uses one-to-one orthologs, HE, one-to-one orthologs plus one-to-many and many-to-many orthologs matched by higher average expression level, SH, one-to-one orthologs plus one-to-many and many-to-many orthologs matched by stronger homology confidence.

Heart_hs_mf

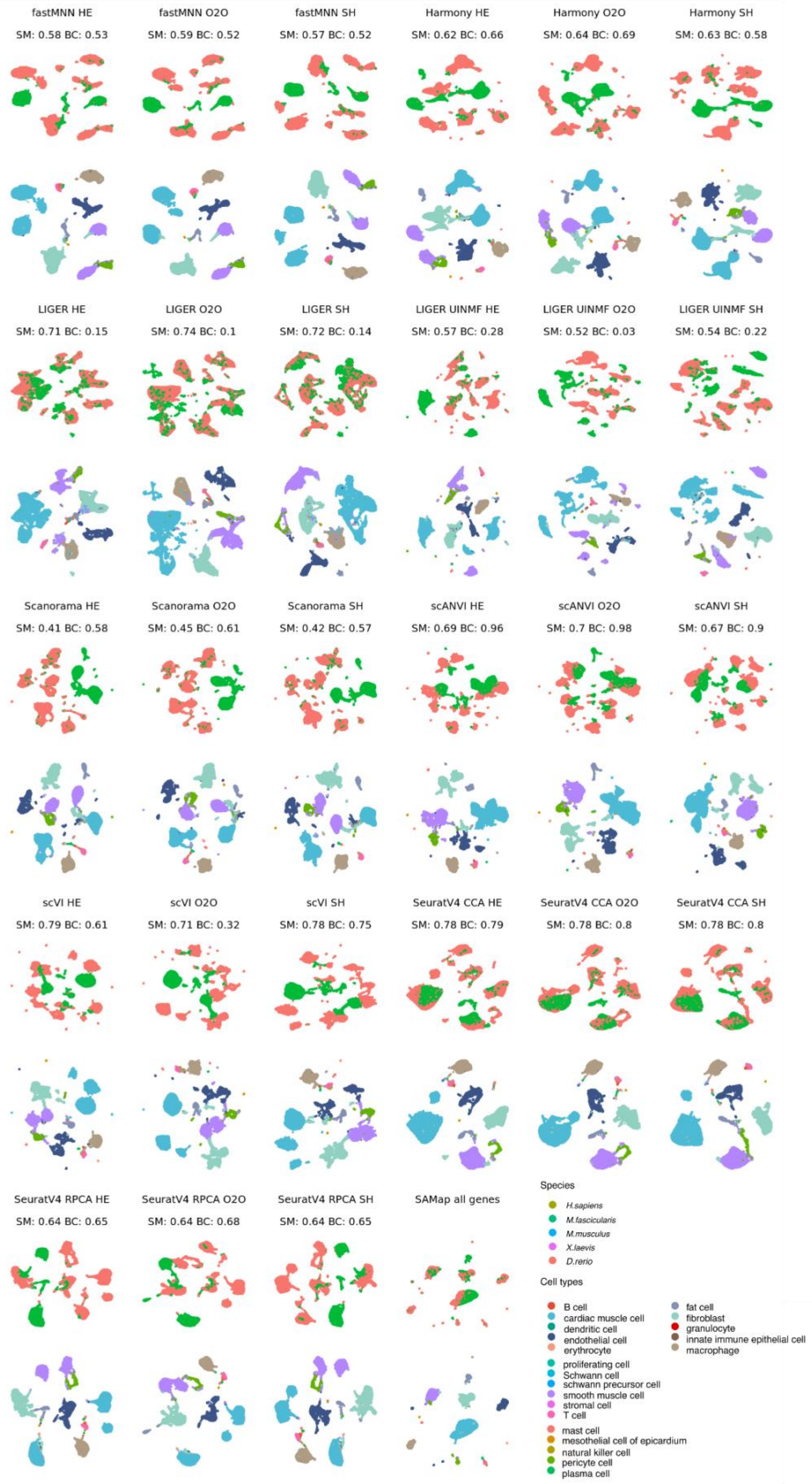
fastMNN HE
SM: 0.58 BC: 0.53
fastMNN O2O
SM: 0.59 BC: 0.52
fastMNN SH
SM: 0.57 BC: 0.52
Harmony HE
SM: 0.62 BC: 0.66
Harmony O2O
SM: 0.64 BC: 0.69
Harmony SH
SM: 0.63 BC: 0.58
LIGER HE
SM: 0.71 BC: 0.15
LIGER O2O
SM: 0.74 BC: 0.1
LIGER SH
SM: 0.72 BC: 0.14
LIGER UINMF HE
SM: 0.57 BC: 0.28
LIGER UINMF O2O
SM: 0.52 BC: 0.03
LIGER UINMF SH
SM: 0.54 BC: 0.22
Scanorama HE
SM: 0.41 BC: 0.58
Scanorama O2O
SM: 0.45 BC: 0.61
Scanorama SH
SM: 0.42 BC: 0.57
scANVI HE
SM: 0.69 BC: 0.96
scANVI O2O
SM: 0.7 BC: 0.98
scANVI SH
SM: 0.67 BC: 0.9
scVI HE
SM: 0.79 BC: 0.61
scVI O2O
SM: 0.71 BC: 0.32
scVI SH
SM: 0.78 BC: 0.75
SeuratV4 CCA HE
SM: 0.78 BC: 0.79
SeuratV4 CCA O2O
SM: 0.78 BC: 0.8
SeuratV4 CCA SH
SM: 0.78 BC: 0.8
SeuratV4 RPCA HE
SM: 0.64 BC: 0.65
SeuratV4 RPCA O2O
SM: 0.64 BC: 0.68
SeuratV4 RPCA SH
SM: 0.64 BC: 0.65
SAMap all genes
Species
H.sapiens
M.fascicularis
M.musculus
X.laevis
D.rerio
Cell types
B cell
cardiac muscle cell
dendritic cell
endothelial cell
erythrocyte
proliferating cell
Schwann cell
schwann precursor cell
smooth muscle cell
stromal cell
T cell
mast cell
mesothelial cell of epicardium
natural killer cell
pericyte cell
plasma cell
fat cell
fibroblast
granulocyte
innate immune epithelial cell
macrophage

Supplementary Figure 30 UMAP visualisation of integration results from 28 strategies in Heart_hs_mf task. Larger images available at the BENGAL reproducibility repository on GitHub [2]. UMAP, Uniform Manifold Approximation and Projection, SM, species mixing score, BC, biology conservation score, O2O, only uses one-to-one orthologs, HE, one-to-one orthologs plus one-to-many and many-to-many orthologs matched by higher average expression level, SH, one-to-one orthologs plus one-to-many and many-to-many orthologs matched by stronger homology confidence.

Heart_hs_mf_mm

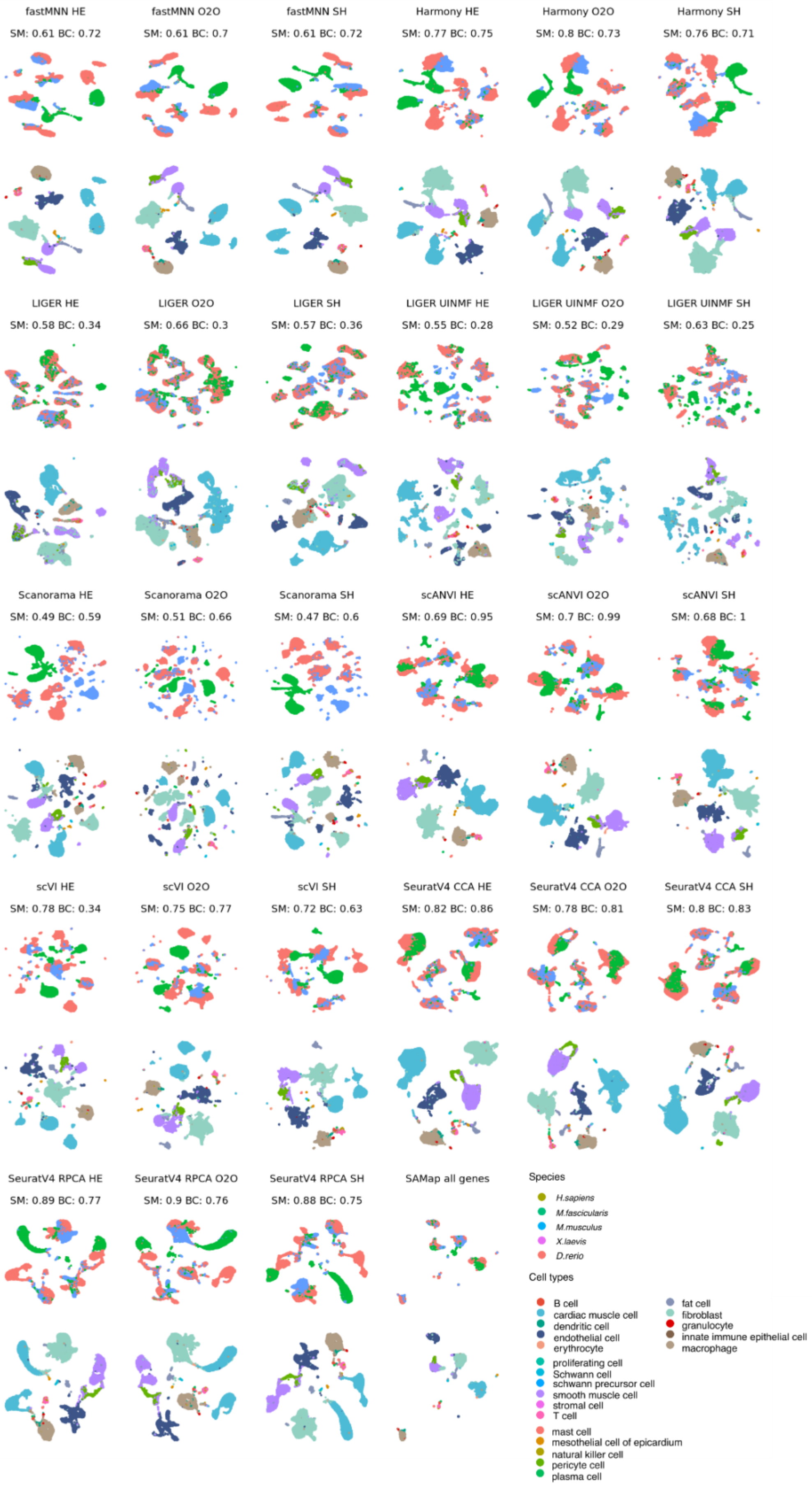

Supplementary Figure 31 UMAP visualisation of integration results from 28 strategies in Heart_hs_mf_mm task. Larger images available at the BENGAL reproducibility repository on GitHub [2]. UMAP, Uniform Manifold Approximation and Projection, SM, species mixing score, BC, biology conservation score, O2O, only uses one-to-one orthologs, HE, one-to-one orthologs plus one-to-many and many-to-many orthologs matched by higher average expression level, SH, one-to-one orthologs plus one-to-many and many-to-many orthologs matched by stronger homology confidence.

Heart_hs_mf_mm_xl

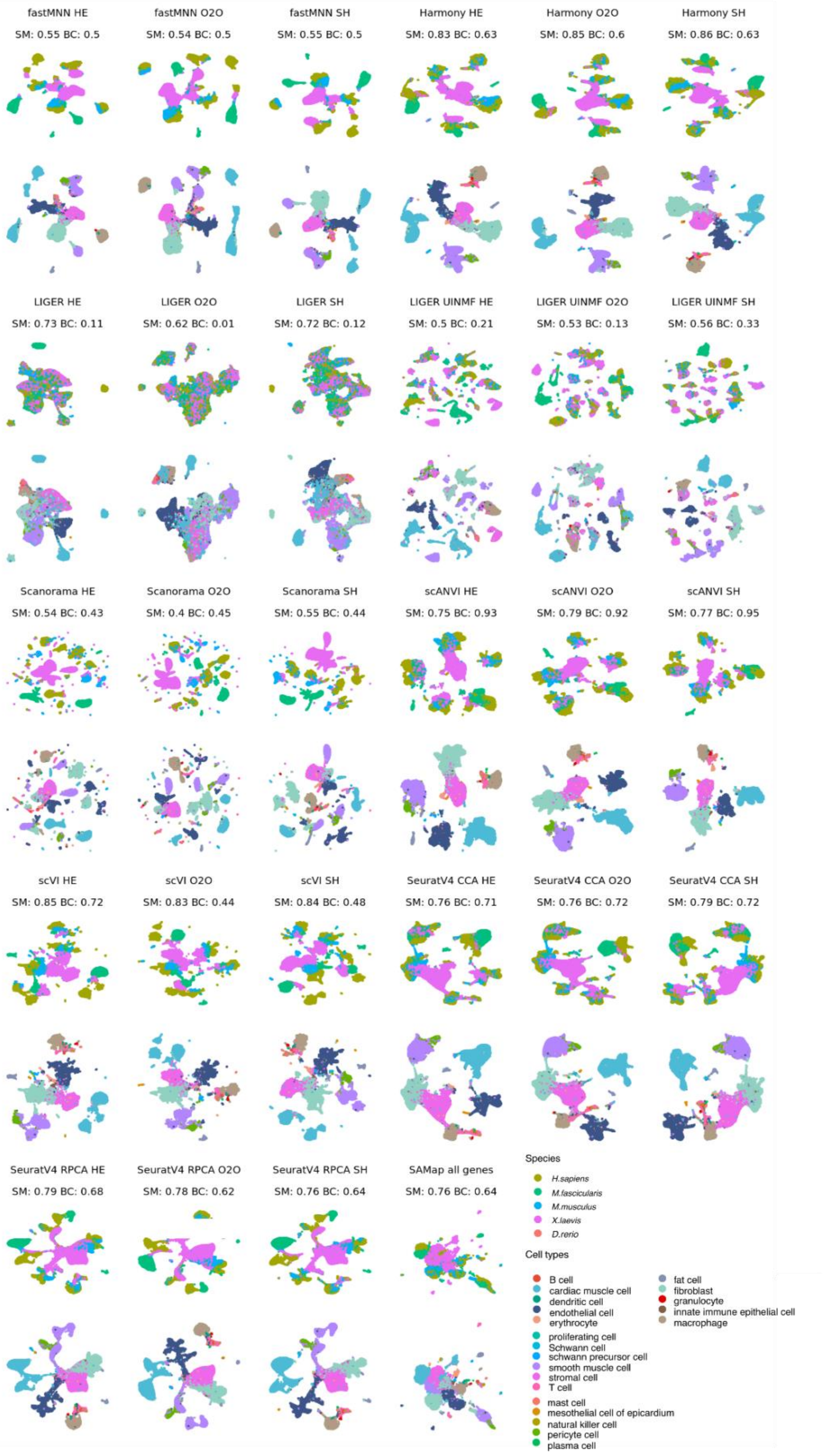

Supplementary Figure 32 UMAP visualisation of integration results from 28 strategies in Heart_hs_mf_mm_xl task. Larger images available at the BENGAL reproducibility repository on GitHub [2]. UMAP, Uniform Manifold Approximation and Projection, SM, species mixing score, BC, biology conservation score, O2O, only uses one-to-one orthologs, HE, one-to-one orthologs plus one-to-many and many-to-many orthologs matched by higher average expression level, SH, one-to-one orthologs plus one-to-many and many-to-many orthologs matched by stronger homology confidence.

Heart_hs_mf_mm_xl_dr

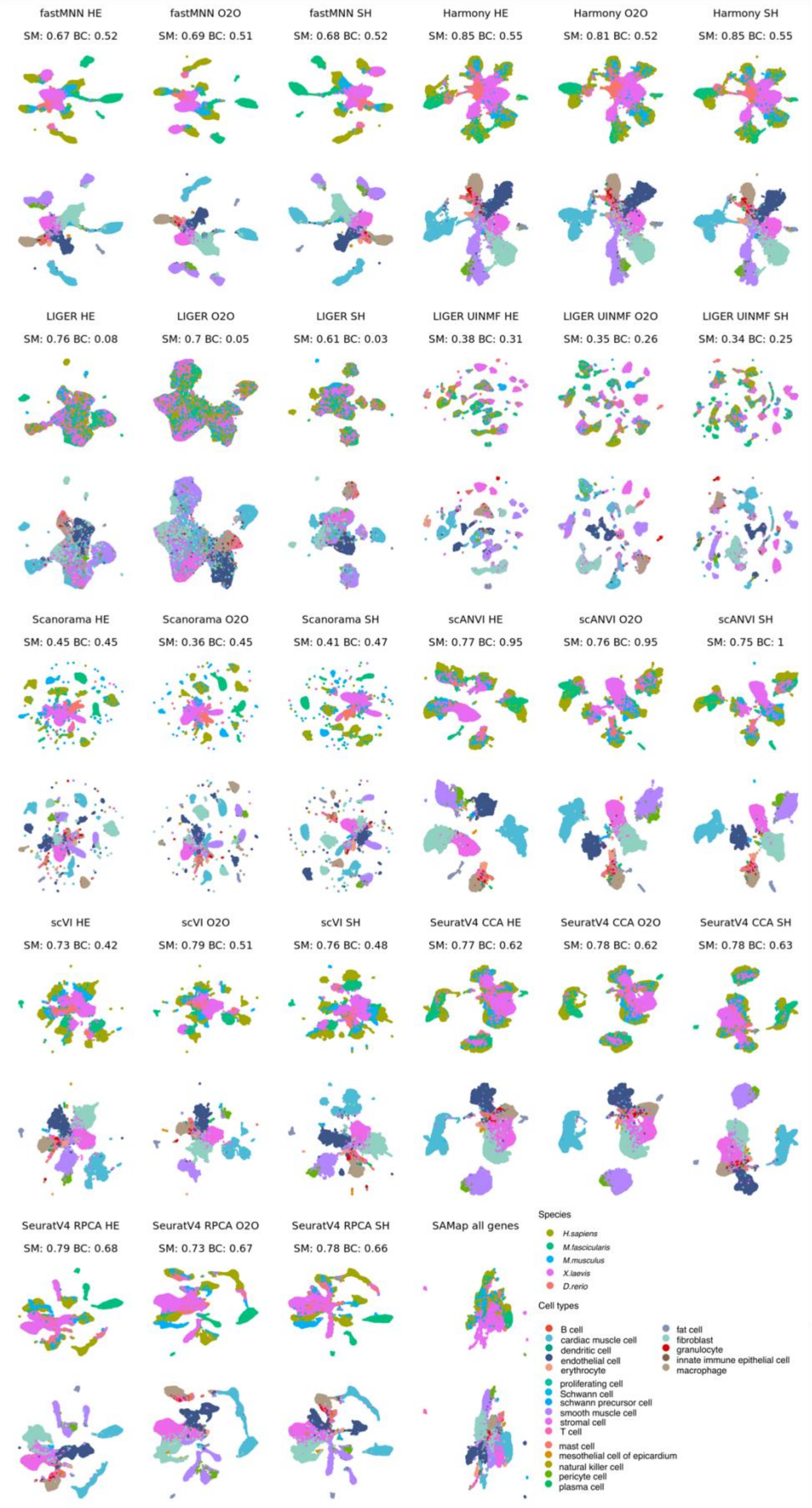

Supplementary Figure 33 UMAP visualisation of integration results from 28 strategies in Heart_hs_mf_mm_xl_dr task. Larger images available at the BENGAL reproducibility repository on GitHub [2]. UMAP, Uniform Manifold Approximation and Projection, SM, species mixing score, BC, biology conservation score, O2O, only uses one-to-one orthologs, HE, one-to-one orthologs plus one-to-many and many-to-many orthologs matched by higher average expression level, SH, one-to-one orthologs plus one-to-many and many-to-many orthologs matched by stronger homology confidence.

Heart_hs_mm

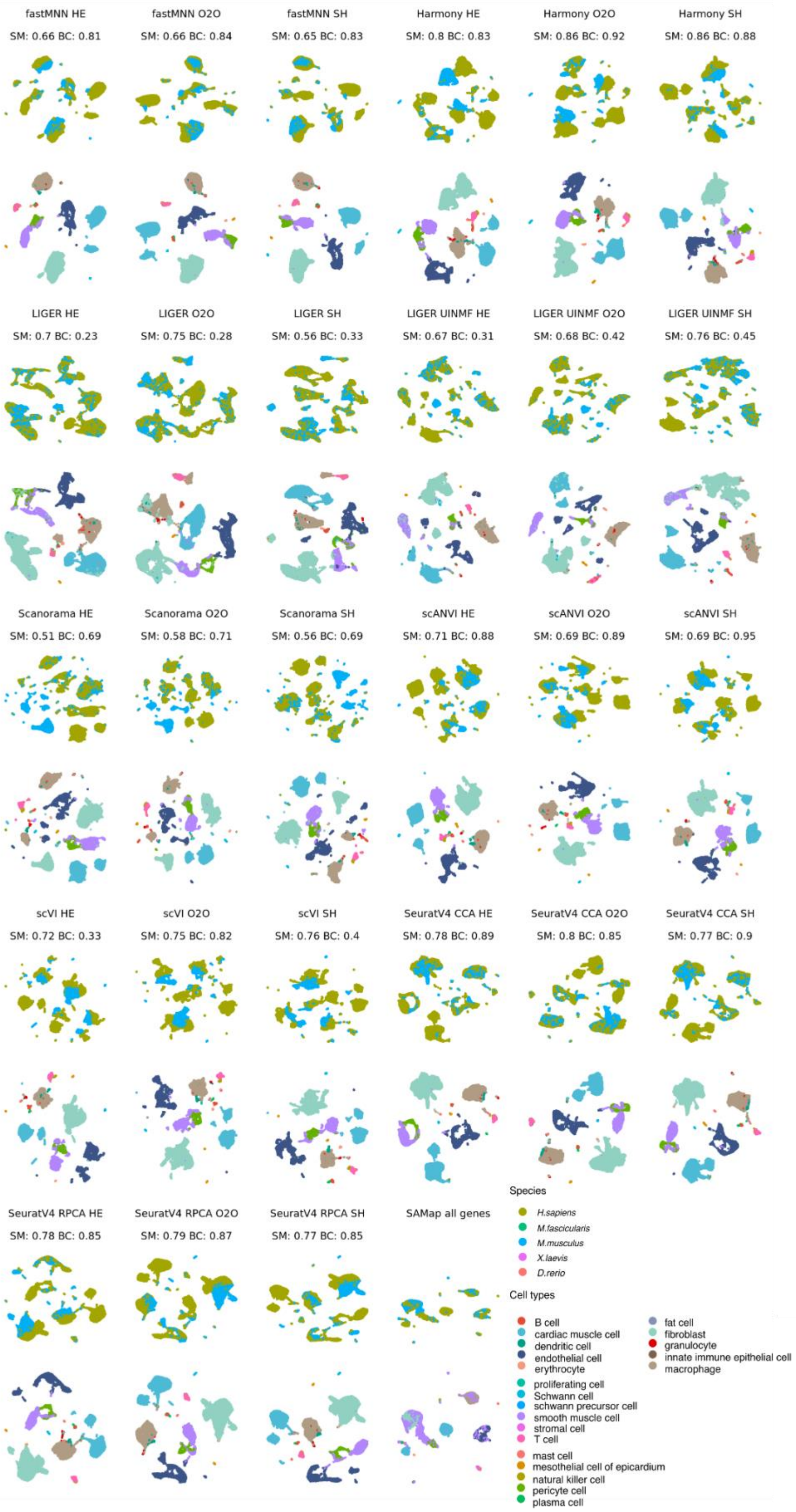
fastMNN HE
SM: 0.66 BC: 0.81
fastMNN O2O
SM: 0.66 BC: 0.84
fastMNN SH
SM: 0.65 BC: 0.83
Harmony HE
SM: 0.8 BC: 0.83
Harmony O2O
SM: 0.86 BC: 0.92
Harmony SH
SM: 0.86 BC: 0.88
LIGER HE
SM: 0.7 BC: 0.23
LIGER O2O
SM: 0.75 BC: 0.28
LIGER SH
SM: 0.56 BC: 0.33
LIGER UINMF HE
SM: 0.67 BC: 0.31
LIGER UINMF O2O
SM: 0.68 BC: 0.42
LIGER UINMF SH
SM: 0.76 BC: 0.45
Scanorama HE
SM: 0.51 BC: 0.69
Scanorama O2O
SM: 0.58 BC: 0.71
Scanorama SH
SM: 0.56 BC: 0.69
scANVI HE
SM: 0.71 BC: 0.88
scANVI O2O
SM: 0.69 BC: 0.89
scANVI SH
SM: 0.69 BC: 0.95
scVI HE
SM: 0.72 BC: 0.33
scVI O2O
SM: 0.75 BC: 0.82
scVI SH
SM: 0.76 BC: 0.4
SeuratV4 CCA HE
SM: 0.78 BC: 0.89
SeuratV4 CCA O2O
SM: 0.8 BC: 0.85
SeuratV4 CCA SH
SM: 0.77 BC: 0.9
SeuratV4 RPCA HE
SM: 0.78 BC: 0.85
SeuratV4 RPCA O2O
SM: 0.79 BC: 0.87
SeuratV4 RPCA SH
SM: 0.77 BC: 0.85
SAMap all genes
Species
H.sapiens
M.fascicularis
M.musculus
X.laevis
D.rerio
Cell types
B cell
cardiac muscle cell
dendritic cell
endothelial cell
erythrocyte
proliferating cell
Schwann cell
schwann precursor cell
smooth muscle cell
stromal cell
T cell
mast cell
mesothelial cell of epicardium
natural killer cell
pericyte cell
plasma cell
fat cell
fibroblast
granulocyte
innate immune epithelial cell
macrophage

Supplementary Figure 34 UMAP visualisation of integration results from 28 strategies in Heart_hs_mm task. Larger images available at the BENGAL reproducibility repository on GitHub [2]. UMAP, Uniform Manifold Approximation and Projection, SM, species mixing score, BC, biology conservation score, O2O, only uses one-to-one orthologs, HE, one-to-one orthologs plus one-to-many and many-to-many orthologs matched by higher average expression level, SH, one-to-one orthologs plus one-to-many and many-to-many orthologs matched by stronger homology confidence.

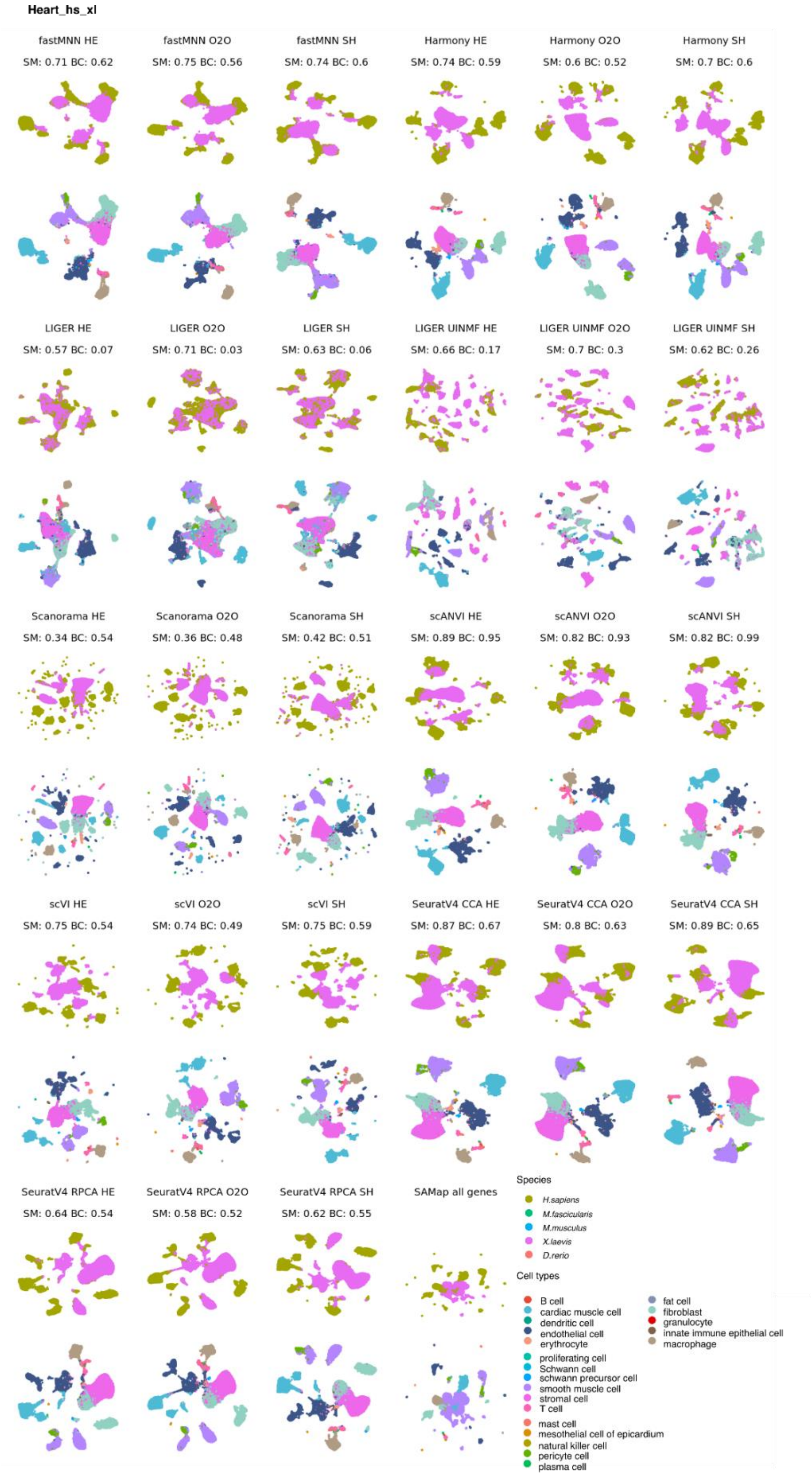
Heart_hs_xl
fastMNN HE
SM: 0.71 BC: 0.62
fastMNN O2O
SM: 0.75 BC: 0.56
fastMNN SH
SM: 0.74 BC: 0.6
Harmony HE
SM: 0.74 BC: 0.59
Harmony O2O
SM: 0.6 BC: 0.52
Harmony SH
SM: 0.7 BC: 0.6
LIGER HE
SM: 0.57 BC: 0.07
LIGER O2O
SM: 0.71 BC: 0.03
LIGER SH
SM: 0.63 BC: 0.06
LIGER UINMF HE
SM: 0.66 BC: 0.17
LIGER UINMF O2O
SM: 0.7 BC: 0.3
LIGER UINMF SH
SM: 0.62 BC: 0.26
Scanorama HE
SM: 0.34 BC: 0.54
Scanorama O2O
SM: 0.36 BC: 0.48
Scanorama SH
SM: 0.42 BC: 0.51
scANVI HE
SM: 0.89 BC: 0.95
scANVI O2O
SM: 0.82 BC: 0.93
scANVI SH
SM: 0.82 BC: 0.99
scVI HE
SM: 0.75 BC: 0.54
scVI O2O
SM: 0.74 BC: 0.49
scVI SH
SM: 0.75 BC: 0.59
SeuratV4 CCA HE
SM: 0.87 BC: 0.67
SeuratV4 CCA O2O
SM: 0.8 BC: 0.63
SeuratV4 CCA SH
SM: 0.89 BC: 0.65
SeuratV4 RPCA HE
SM: 0.64 BC: 0.54
SeuratV4 RPCA O2O
SM: 0.58 BC: 0.52
SeuratV4 RPCA SH
SM: 0.62 BC: 0.55
SAMap all genes
Species
H.sapiens
M.fascicularis
M.musculus
X.laevis
D.rerio
Cell types
B cell
cardiac muscle cell
dendritic cell
endothelial cell
erythrocyte
proliferating cell
Schwann cell
schwann precursor cell
smooth muscle cell
stromal cell
T cell
mast cell
mesothelial cell of epicardium
natural killer cell
pericyte cell
plasma cell
fat cell
fibroblast
granulocyte
innate immune epithelial cell
macrophage

Supplementary Figure 35 UMAP visualisation of integration results from 28 strategies in Heart_hs_xl task. Larger images available at the BENGAL reproducibility repository on GitHub [2]. UMAP, Uniform Manifold Approximation and Projection, SM, species mixing score, BC, biology conservation score, O2O, only uses one-to-one orthologs, HE, one-to-one orthologs plus one-to-many and many-to-many orthologs matched by higher average expression level, SH, one-to-one orthologs plus one-to-many and many-to-many orthologs matched by stronger homology confidence.

Heart_hs_dr

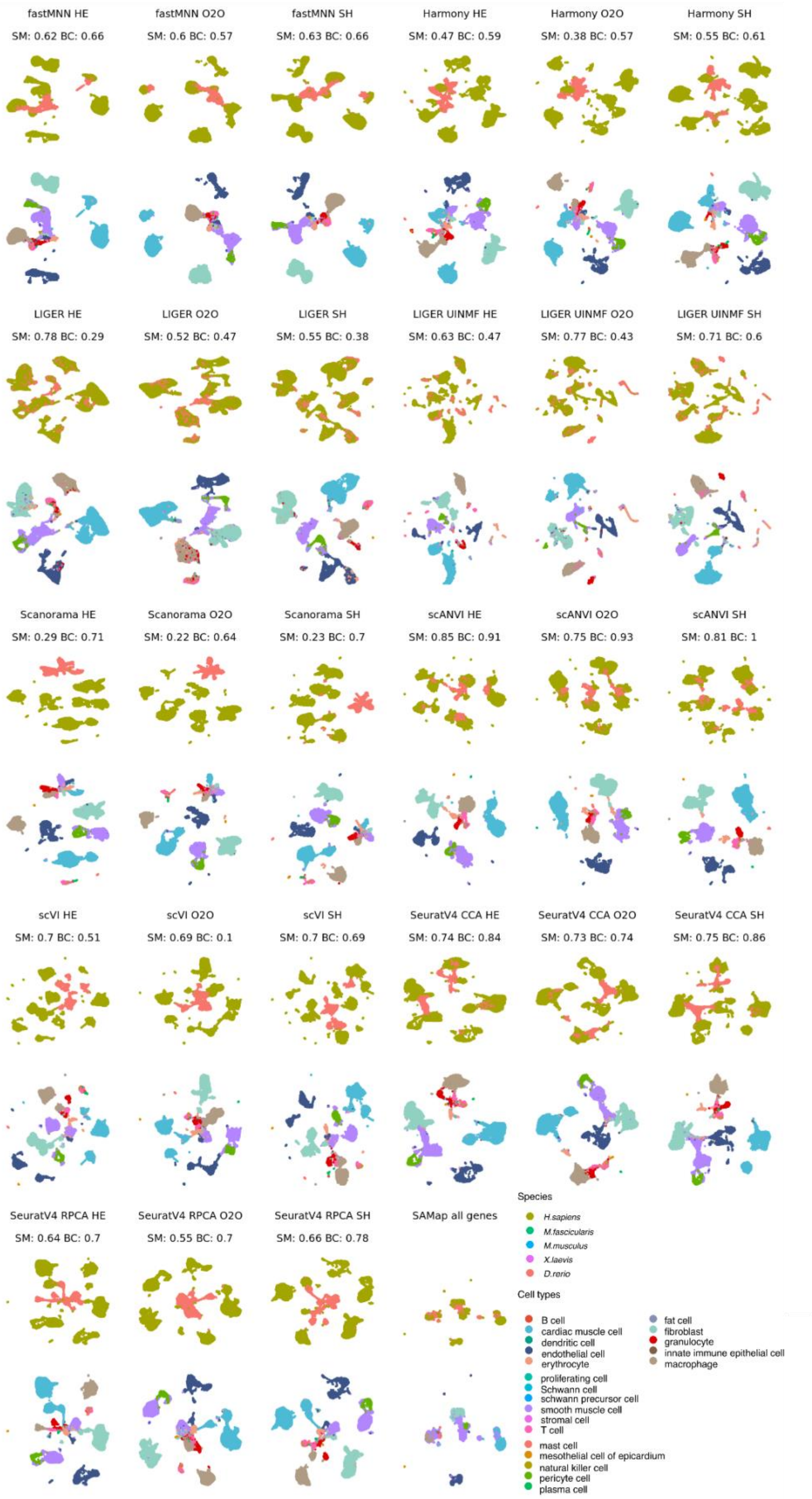
fastMNN HE
SM: 0.62 BC: 0.66
fastMNN O2O
SM: 0.6 BC: 0.57
fastMNN SH
SM: 0.63 BC: 0.66
Harmony HE
SM: 0.47 BC: 0.59
Harmony O2O
SM: 0.38 BC: 0.57
Harmony SH
SM: 0.55 BC: 0.61
LIGER HE
SM: 0.78 BC: 0.29
LIGER O2O
SM: 0.52 BC: 0.47
LIGER SH
SM: 0.55 BC: 0.38
LIGER UINMF HE
SM: 0.63 BC: 0.47
LIGER UINMF O2O
SM: 0.77 BC: 0.43
LIGER UINMF SH
SM: 0.71 BC: 0.6
Scanorama HE
SM: 0.29 BC: 0.71
Scanorama O2O
SM: 0.22 BC: 0.64
Scanorama SH
SM: 0.23 BC: 0.7
scANVI HE
SM: 0.85 BC: 0.91
scANVI O2O
SM: 0.75 BC: 0.93
scANVI SH
SM: 0.81 BC: 1
scVI HE
SM: 0.7 BC: 0.51
scVI O2O
SM: 0.69 BC: 0.1
scVI SH
SM: 0.7 BC: 0.69
SeuratV4 CCA HE
SM: 0.74 BC: 0.84
SeuratV4 CCA O2O
SM: 0.73 BC: 0.74
SeuratV4 CCA SH
SM: 0.75 BC: 0.86
SeuratV4 RPCA HE
SM: 0.64 BC: 0.7
SeuratV4 RPCA O2O
SM: 0.55 BC: 0.7
SeuratV4 RPCA SH
SM: 0.66 BC: 0.78
SAMap all genes
Species
H.sapiens
M.fascicularis
M.musculus
X.laevis
D.rerio
Cell types
B cell
cardiac muscle cell
dendritic cell
endothelial cell
erythrocyte
proliferating cell
Schwann cell
schwann precursor cell
smooth muscle cell
stromal cell
T cell
mast cell
mesothelial cell of epicardium
natural killer cell
pericyte cell
plasma cell
fat cell
fibroblast
granulocyte
innate immune epithelial cell
macrophage

Supplementary Figure 36 UMAP visualisation of integration results from 28 strategies in Heart_hs_dr task. Larger images available at the BENGAL reproducibility repository on GitHub [2]. UMAP, Uniform Manifold Approximation and Projection, SM, species mixing score, BC, biology conservation score, O2O, only uses one-to-one orthologs, HE, one-to-one orthologs plus one-to-many and many-to-many orthologs matched by higher average expression level, SH, one-to-one orthologs plus one-to-many and many-to-many orthologs matched by stronger homology confidence.

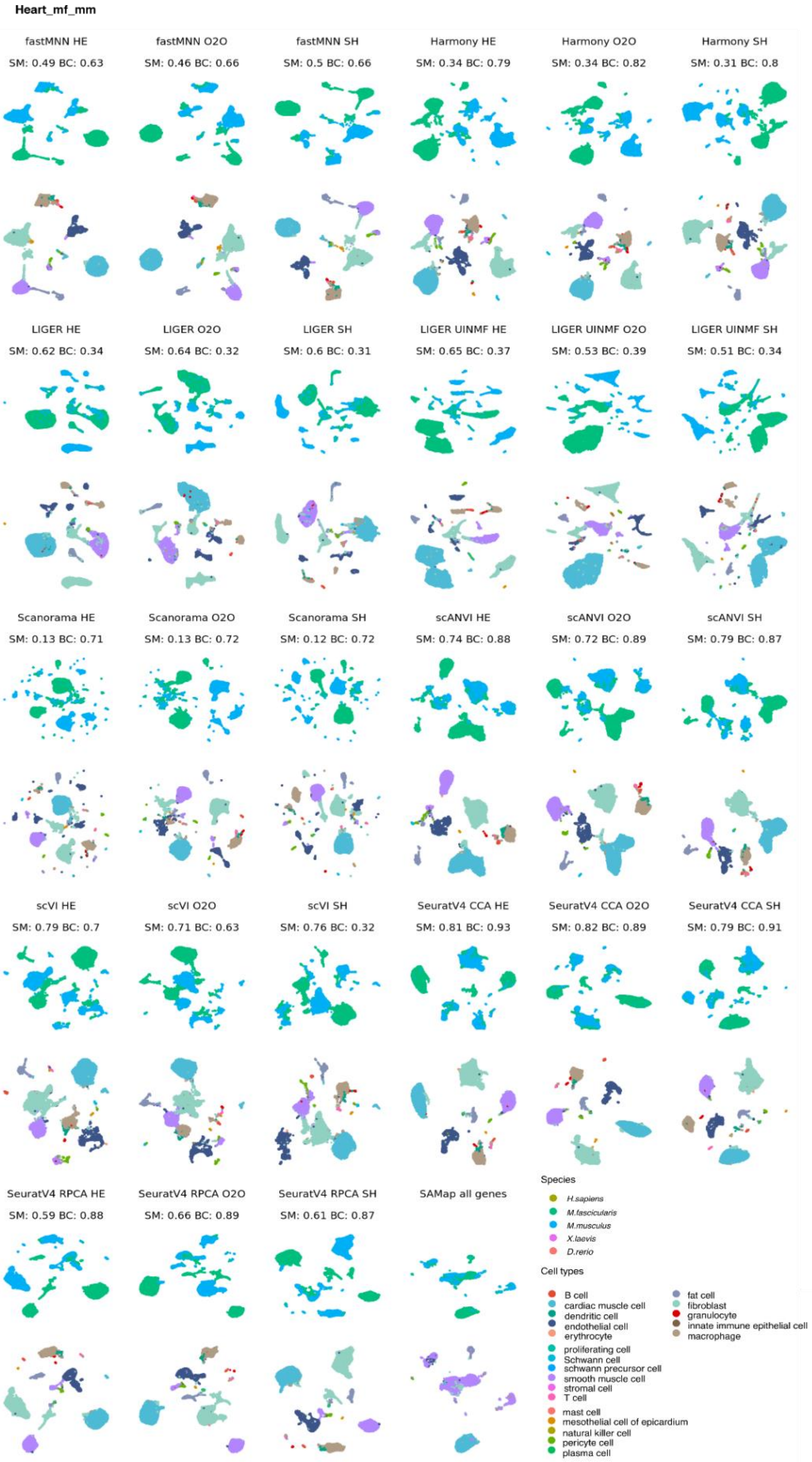
Heart_mf_mm
fastMNN HE
SM: 0.49 BC: 0.63
fastMNN O2O
SM: 0.46 BC: 0.66
fastMNN SH
SM: 0.5 BC: 0.66
Harmony HE
SM: 0.34 BC: 0.79
Harmony O2O
SM: 0.34 BC: 0.82
Harmony SH
SM: 0.31 BC: 0.8
LIGER HE
SM: 0.62 BC: 0.34
LIGER O2O
SM: 0.64 BC: 0.32
LIGER SH
SM: 0.6 BC: 0.31
LIGER UINMF HE
SM: 0.65 BC: 0.37
LIGER UINMF O2O
SM: 0.53 BC: 0.39
LIGER UINMF SH
SM: 0.51 BC: 0.34
Scanorama HE
SM: 0.13 BC: 0.71
Scanorama O2O
SM: 0.13 BC: 0.72
Scanorama SH
SM: 0.12 BC: 0.72
scANVI HE
SM: 0.74 BC: 0.88
scANVI O2O
SM: 0.72 BC: 0.89
scANVI SH
SM: 0.79 BC: 0.87
scVI HE
SM: 0.79 BC: 0.7
scVI O2O
SM: 0.71 BC: 0.63
scVI SH
SM: 0.76 BC: 0.32
SeuratV4 CCA HE
SM: 0.81 BC: 0.93
SeuratV4 CCA O2O
SM: 0.82 BC: 0.89
SeuratV4 CCA SH
SM: 0.79 BC: 0.91
SeuratV4 RPCA HE
SM: 0.59 BC: 0.88
SeuratV4 RPCA O2O
SM: 0.66 BC: 0.89
SeuratV4 RPCA SH
SM: 0.61 BC: 0.87
SAMap all genes
Species
H.sapiens
M.fascicularis
M.musculus
X.laevis
D.rerio
Cell types
B cell
cardiac muscle cell
dendritic cell
endothelial cell
erythrocyte
proliferating cell
Schwann cell
schwann precursor cell
smooth muscle cell
stromal cell
T cell
mast cell
mesothelial cell of epicardium
natural killer cell
pericyte cell
plasma cell
fat cell
fibroblast
granulocyte
innate immune epithelial cell
macrophage

Supplementary Figure 37 UMAP visualisation of integration results from 28 strategies in Heart_mf_mm task. Larger images available at the BENGAL reproducibility repository on GitHub [2]. UMAP, Uniform Manifold Approximation and Projection, SM, species mixing score, BC, biology conservation score, O2O, only uses one-to-one orthologs, HE, one-to-one orthologs plus one-to-many and many-to-many orthologs matched by higher average expression level, SH, one-to-one orthologs plus one-to-many and many-to-many orthologs matched by stronger homology confidence.

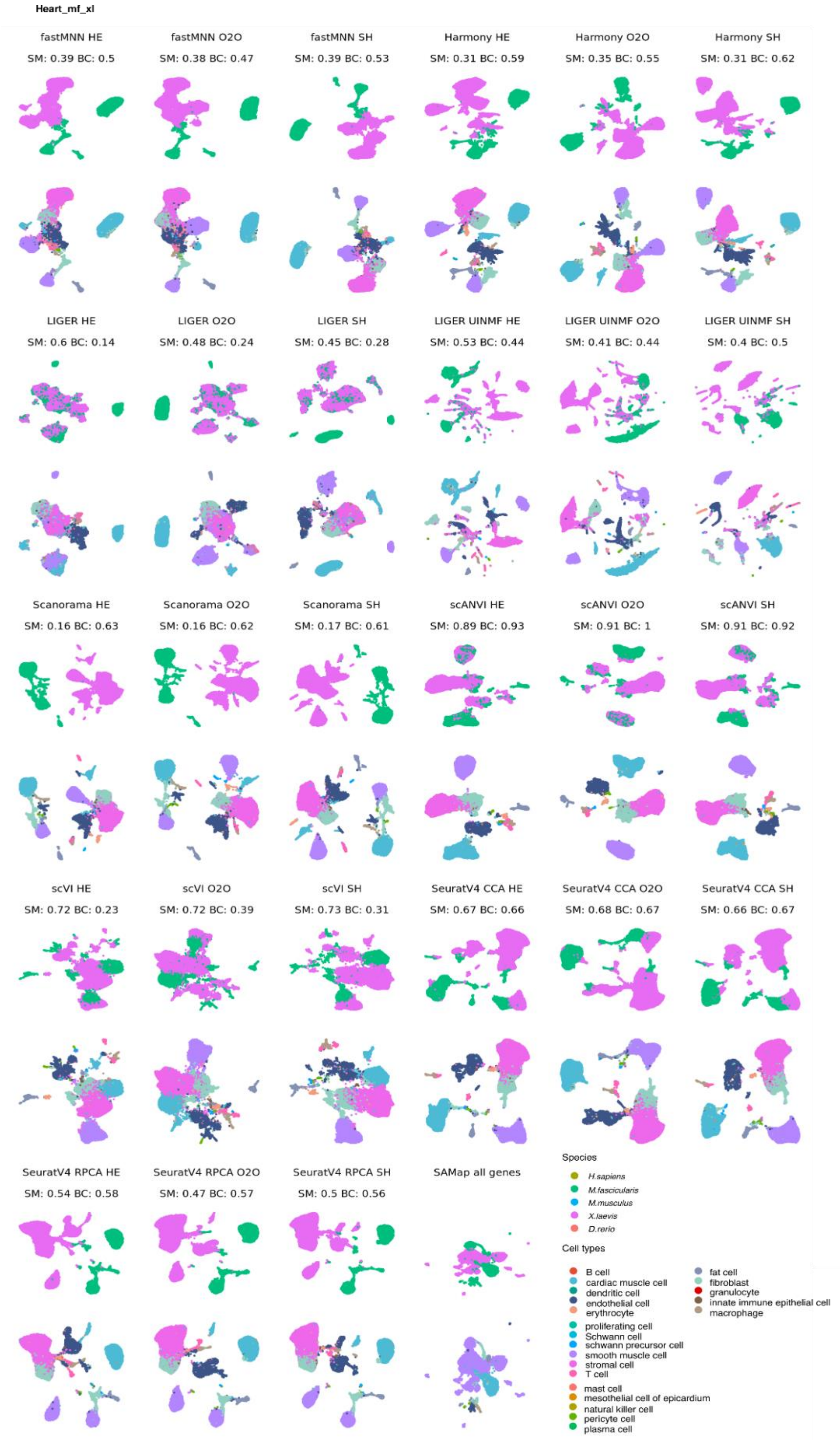
Heart_mf_xl
fastMNN HE
SM: 0.39 BC: 0.5
fastMNN O2O
SM: 0.38 BC: 0.47
fastMNN SH
SM: 0.39 BC: 0.53
Harmony HE
SM: 0.31 BC: 0.59
Harmony O2O
SM: 0.35 BC: 0.55
Harmony SH
SM: 0.31 BC: 0.62
LIGER HE
SM: 0.6 BC: 0.14
LIGER O2O
SM: 0.48 BC: 0.24
LIGER SH
SM: 0.45 BC: 0.28
LIGER UINMF HE
SM: 0.53 BC: 0.44
LIGER UINMF O2O
SM: 0.41 BC: 0.44
LIGER UINMF SH
SM: 0.4 BC: 0.5
Scanorama HE
SM: 0.16 BC: 0.63
Scanorama O2O
SM: 0.16 BC: 0.62
Scanorama SH
SM: 0.17 BC: 0.61
scANVI HE
SM: 0.89 BC: 0.93
scANVI O2O
SM: 0.91 BC: 1
scANVI SH
SM: 0.91 BC: 0.92
scVI HE
SM: 0.72 BC: 0.23
scVI O2O
SM: 0.72 BC: 0.39
scVI SH
SM: 0.73 BC: 0.31
SeuratV4 CCA HE
SM: 0.67 BC: 0.66
SeuratV4 CCA O2O
SM: 0.68 BC: 0.67
SeuratV4 CCA SH
SM: 0.66 BC: 0.67
SeuratV4 RPCA HE
SM: 0.54 BC: 0.58
SeuratV4 RPCA O2O
SM: 0.47 BC: 0.57
SeuratV4 RPCA SH
SM: 0.5 BC: 0.56
SAMap all genes
Species
H.sapiens
M.fascicularis
M.musculus
X.laevis
D.rerio
Cell types
B cell
cardiac muscle cell
dendritic cell
endothelial cell
erythrocyte
proliferating cell
Schwann cell
schwann precursor cell
smooth muscle cell
stromal cell
T cell
mast cell
mesothelial cell of epicardium
natural killer cell
pericyte cell
plasma cell
fat cell
fibroblast
granulocyte
innate immune epithelial cell
macrophage

Supplementary Figure 38 UMAP visualisation of integration results from 28 strategies in Heart_mf_xl task. Larger images available at the BENGAL reproducibility repository on GitHub [2]. UMAP, Uniform Manifold Approximation and Projection, SM, species mixing score, BC, biology conservation score, O2O, only uses one-to-one orthologs, HE, one-to-one orthologs plus one-to-many and many-to-many orthologs matched by higher average expression level, SH, one-to-one orthologs plus one-to-many and many-to-many orthologs matched by stronger homology confidence.

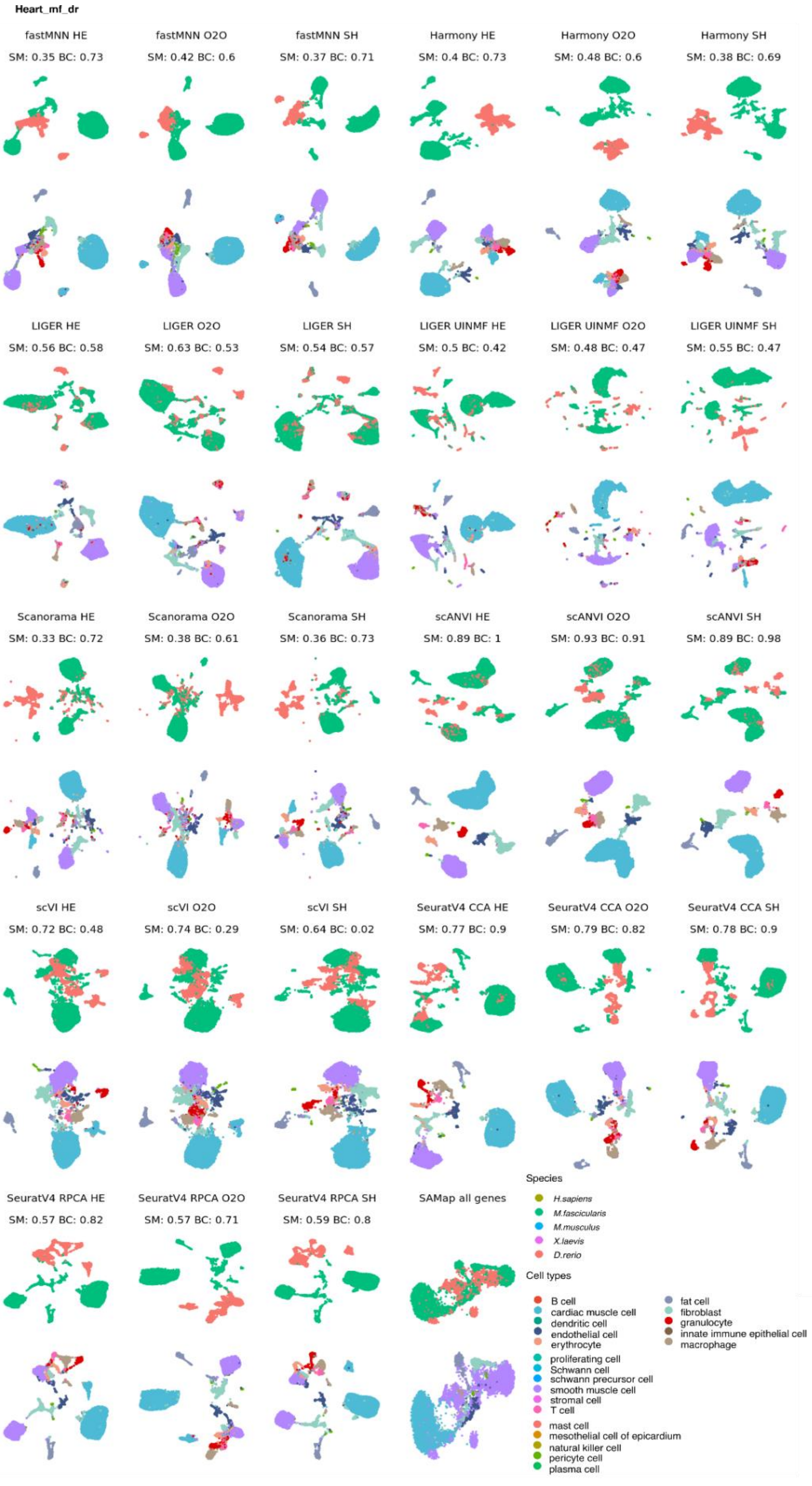
Heart_mf_dr
fastMNN HE
SM: 0.35 BC: 0.73
fastMNN O2O
SM: 0.42 BC: 0.6
fastMNN SH
SM: 0.37 BC: 0.71
Harmony HE
SM: 0.4 BC: 0.73
Harmony O2O
SM: 0.48 BC: 0.6
Harmony SH
SM: 0.38 BC: 0.69
LIGER HE
SM: 0.56 BC: 0.58
LIGER O2O
SM: 0.63 BC: 0.53
LIGER SH
SM: 0.54 BC: 0.57
LIGER UINMF HE
SM: 0.5 BC: 0.42
LIGER UINMF O2O
SM: 0.48 BC: 0.47
LIGER UINMF SH
SM: 0.55 BC: 0.47
Scanorama HE
SM: 0.33 BC: 0.72
Scanorama O2O
SM: 0.38 BC: 0.61
Scanorama SH
SM: 0.36 BC: 0.73
scANVI HE
SM: 0.89 BC: 1
scANVI O2O
SM: 0.93 BC: 0.91
scANVI SH
SM: 0.89 BC: 0.98
scVI HE
SM: 0.72 BC: 0.48
scVI O2O
SM: 0.74 BC: 0.29
scVI SH
SM: 0.64 BC: 0.02
SeuratV4 CCA HE
SM: 0.77 BC: 0.9
SeuratV4 CCA O2O
SM: 0.79 BC: 0.82
SeuratV4 CCA SH
SM: 0.78 BC: 0.9
SeuratV4 RPCA HE
SM: 0.57 BC: 0.82
SeuratV4 RPCA O2O
SM: 0.57 BC: 0.71
SeuratV4 RPCA SH
SM: 0.59 BC: 0.8
SAMap all genes
Species
H.sapiens
M.fascicularis
M.musculus
X.laevis
D.rerio
Cell types
B cell
cardiac muscle cell
dendritic cell
endothelial cell
erythrocyte
proliferating cell
Schwann cell
schwann precursor cell
smooth muscle cell
stromal cell
T cell
mast cell
mesothelial cell of epicardium
natural killer cell
pericyte cell
plasma cell
fat cell
fibroblast
granulocyte
innate immune epithelial cell
macrophage

Supplementary Figure 39 UMAP visualisation of integration results from 28 strategies in Heart_mf_dr task. Larger images available at the BENGAL reproducibility repository on GitHub [2]. UMAP, Uniform Manifold Approximation and Projection, SM, species mixing score, BC, biology conservation score, O2O, only uses one-to-one orthologs, HE, one-to-one orthologs plus one-to-many and many-to-many orthologs matched by higher average expression level, SH, one-to-one orthologs plus one-to-many and many-to-many orthologs matched by stronger homology confidence.

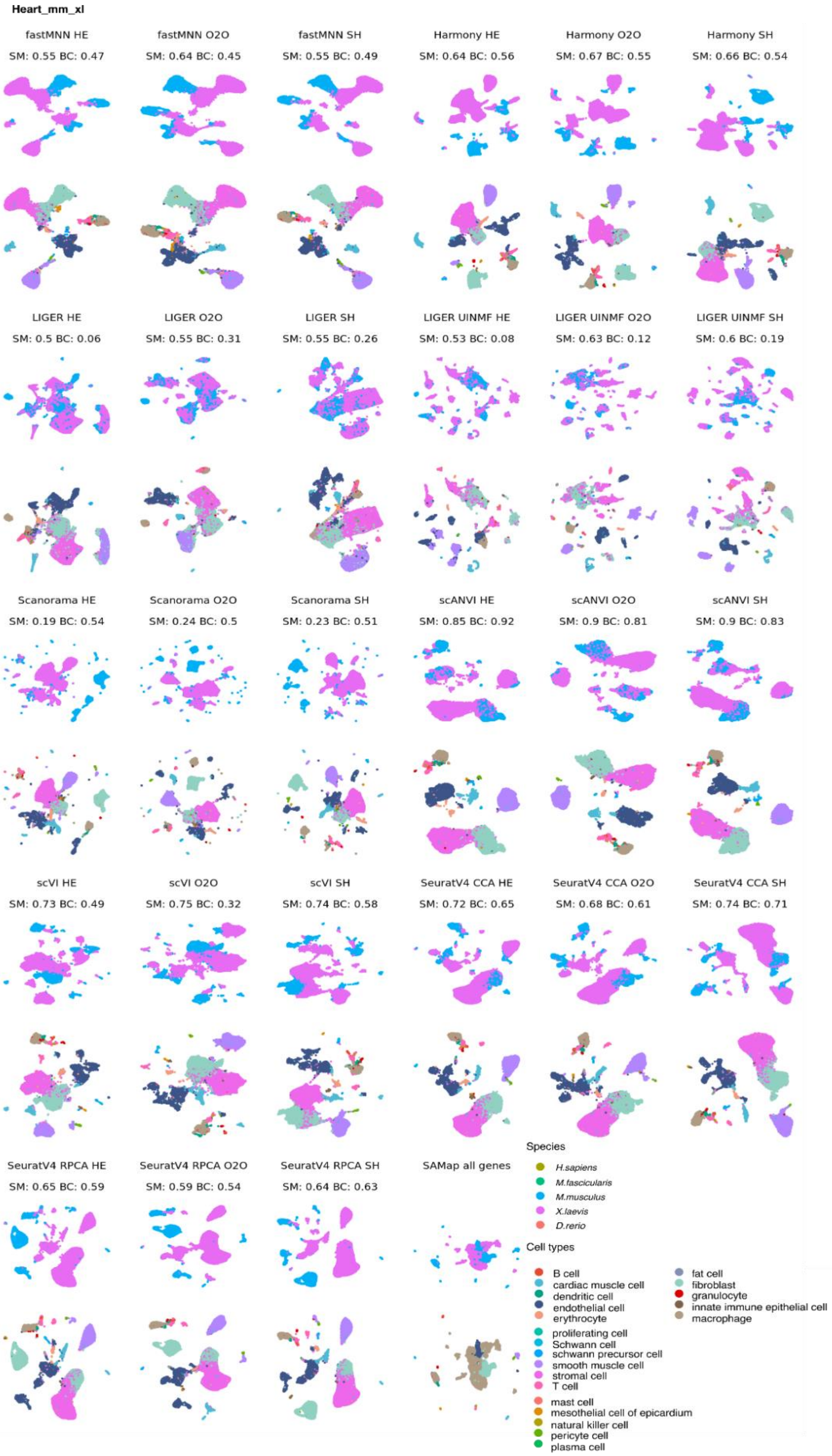
Heart_mm_xl
fastMNN HE
SM: 0.55 BC: 0.47
fastMNN O2O
SM: 0.64 BC: 0.45
fastMNN SH
SM: 0.55 BC: 0.49
Harmony HE
SM: 0.64 BC: 0.56
Harmony O2O
SM: 0.67 BC: 0.55
Harmony SH
SM: 0.66 BC: 0.54
LIGER HE
SM: 0.5 BC: 0.06
LIGER O2O
SM: 0.55 BC: 0.31
LIGER SH
SM: 0.55 BC: 0.26
LIGER UINMF HE
SM: 0.53 BC: 0.08
LIGER UINMF O2O
SM: 0.63 BC: 0.12
LIGER UINMF SH
SM: 0.6 BC: 0.19
Scanorama HE
SM: 0.19 BC: 0.54
Scanorama O2O
SM: 0.24 BC: 0.5
Scanorama SH
SM: 0.23 BC: 0.51
scANVI HE
SM: 0.85 BC: 0.92
scANVI O2O
SM: 0.9 BC: 0.81
scANVI SH
SM: 0.9 BC: 0.83
scVI HE
SM: 0.73 BC: 0.49
scVI O2O
SM: 0.75 BC: 0.32
scVI SH
SM: 0.74 BC: 0.58
SeuratV4 CCA HE
SM: 0.72 BC: 0.65
SeuratV4 CCA O2O
SM: 0.68 BC: 0.61
SeuratV4 CCA SH
SM: 0.74 BC: 0.71
SeuratV4 RPCA HE
SM: 0.65 BC: 0.59
SeuratV4 RPCA O2O
SM: 0.59 BC: 0.54
SeuratV4 RPCA SH
SM: 0.64 BC: 0.63
SAMap all genes
Species
H.sapiens
M.fascicularis
M.musculus
X.laevis
D.rerio
Cell types
B cell
cardiac muscle cell
dendritic cell
endothelial cell
erythrocyte
proliferating cell
Schwann cell
schwann precursor cell
smooth muscle cell
stromal cell
T cell
mast cell
mesothelial cell of epicardium
natural killer cell
pericyte cell
plasma cell
fat cell
fibroblast
granulocyte
innate immune epithelial cell
macrophage

Supplementary Figure 40 UMAP visualisation of integration results from 28 strategies in Heart_mm_xl task. Larger images available at the BENGAL reproducibility repository on GitHub [2]. UMAP, Uniform Manifold Approximation and Projection, SM, species mixing score, BC, biology conservation score, O2O, only uses one-to-one orthologs, HE, one-to-one orthologs plus one-to-many and many-to-many orthologs matched by higher average expression level, SH, one-to-one orthologs plus one-to-many and many-to-many orthologs matched by stronger homology confidence.

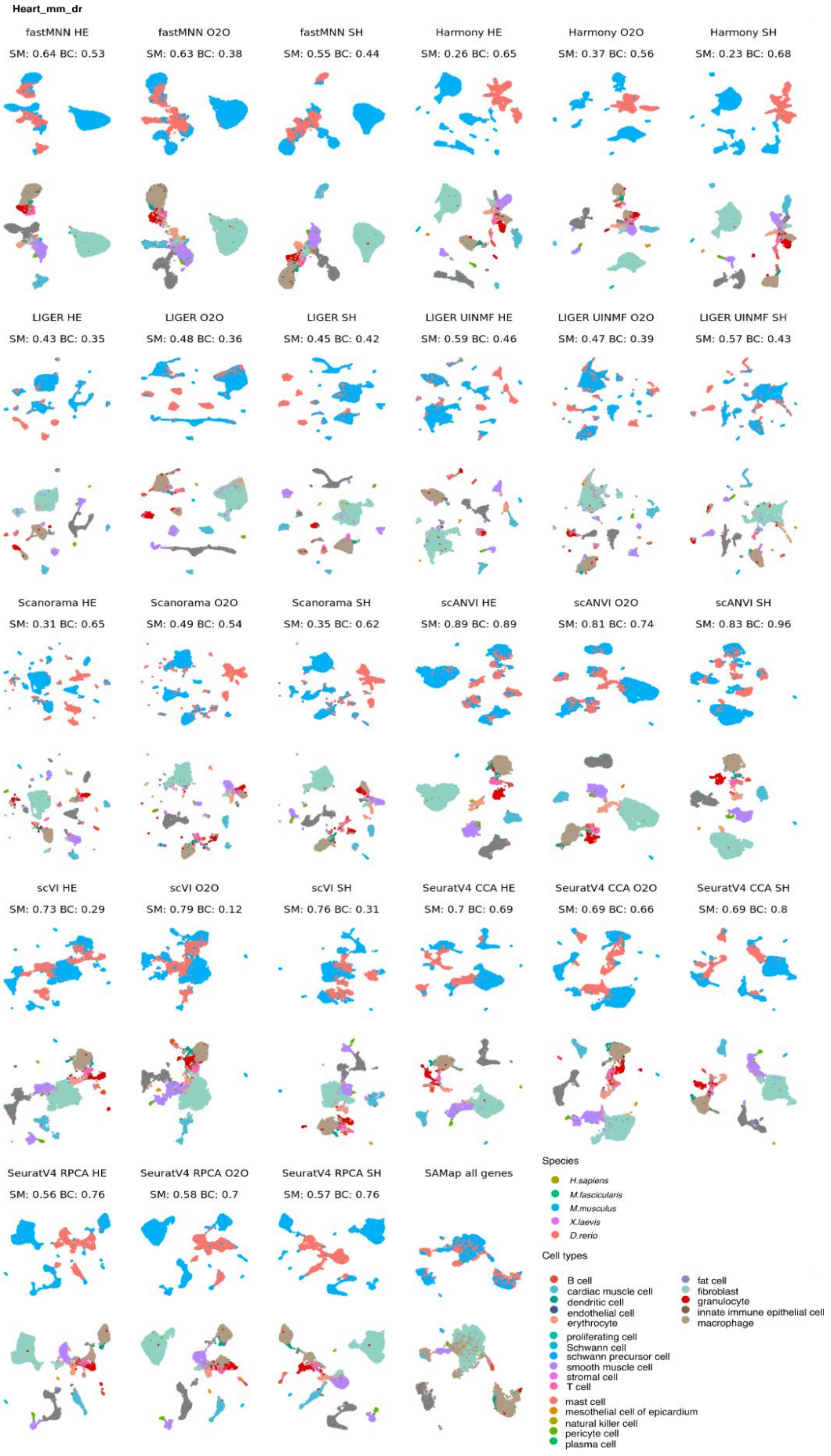
Heart_mm_dr
fastMNN HE
SM: 0.64 BC: 0.53
fastMNN O2O
SM: 0.63 BC: 0.38
fastMNN SH
SM: 0.55 BC: 0.44
Harmony HE
SM: 0.26 BC: 0.65
Harmony O2O
SM: 0.37 BC: 0.56
Harmony SH
SM: 0.23 BC: 0.68
LIGER HE
SM: 0.43 BC: 0.35
LIGER O2O
SM: 0.48 BC: 0.36
LIGER SH
SM: 0.45 BC: 0.42
LIGER UINMF HE
SM: 0.59 BC: 0.46
LIGER UINMF O2O
SM: 0.47 BC: 0.39
LIGER UINMF SH
SM: 0.57 BC: 0.43
Scanorama HE
SM: 0.31 BC: 0.65
Scanorama O2O
SM: 0.49 BC: 0.54
Scanorama SH
SM: 0.35 BC: 0.62
scANVI HE
SM: 0.89 BC: 0.89
scANVI O2O
SM: 0.81 BC: 0.74
scANVI SH
SM: 0.83 BC: 0.96
scVI HE
SM: 0.73 BC: 0.29
scVI O2O
SM: 0.79 BC: 0.12
scVI SH
SM: 0.76 BC: 0.31
SeuratV4 CCA HE
SM: 0.7 BC: 0.69
SeuratV4 CCA O2O
SM: 0.69 BC: 0.66
SeuratV4 CCA SH
SM: 0.69 BC: 0.8
SeuratV4 RPCA HE
SM: 0.56 BC: 0.76
SeuratV4 RPCA O2O
SM: 0.58 BC: 0.7
SeuratV4 RPCA SH
SM: 0.57 BC: 0.76
SAMap all genes
Species
H.sapiens
M.fascicularis
M.musculus
X.laevis
D.rerio
Cell types
B cell
cardiac muscle cell
dendritic cell
endothelial cell
erythrocyte
proliferating cell
Schwann cell
schwann precursor cell
smooth muscle cell
stromal cell
T cell
mast cell
mesothelial cell of epicardium
natural killer cell
pericyte cell
plasma cell
fat cell
fibroblast
granulocyte
innate immune epithelial cell
macrophage

Supplementary Figure 41 UMAP visualisation of integration results from 28 strategies in Heart_mm_dr task. Larger images available at the BENGAL reproducibility repository on GitHub [2]. UMAP, Uniform Manifold Approximation and Projection, SM, species mixing score, BC, biology conservation score, O2O, only uses one-to-one orthologs, HE, one-to-one orthologs plus one-to-many and many-to-many orthologs matched by higher average expression level, SH, one-to-one orthologs plus one-to-many and many-to-many orthologs matched by stronger homology confidence.

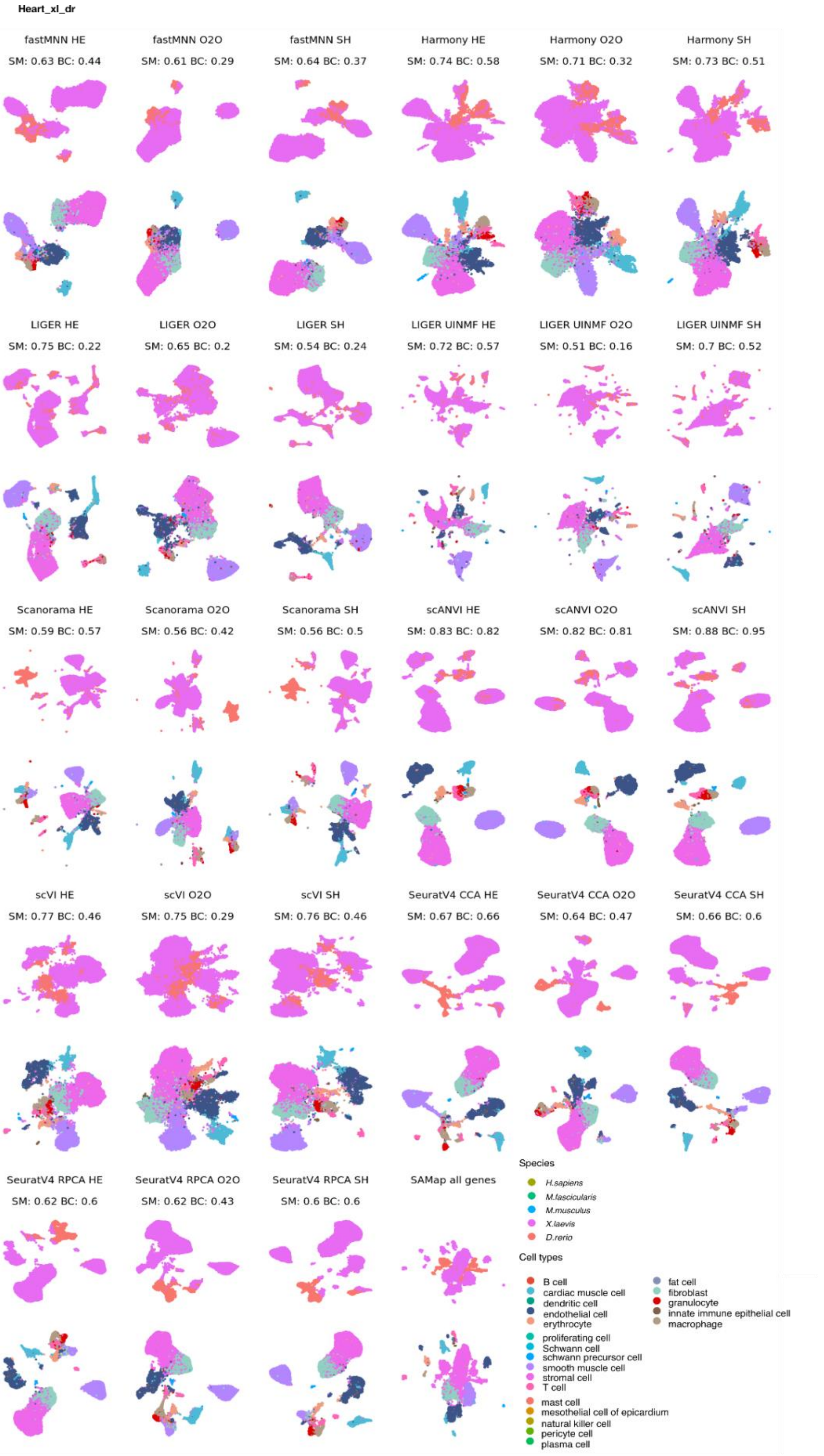
Heart_xl_dr
fastMNN HE
SM: 0.63 BC: 0.44
fastMNN O2O
SM: 0.61 BC: 0.29
fastMNN SH
SM: 0.64 BC: 0.37
Harmony HE
SM: 0.74 BC: 0.58
Harmony O2O
SM: 0.71 BC: 0.32
Harmony SH
SM: 0.73 BC: 0.51
LIGER HE
SM: 0.75 BC: 0.22
LIGER O2O
SM: 0.65 BC: 0.2
LIGER SH
SM: 0.54 BC: 0.24
LIGER UINMF HE
SM: 0.72 BC: 0.57
LIGER UINMF O2O
SM: 0.51 BC: 0.16
LIGER UINMF SH
SM: 0.7 BC: 0.52
Scanorama HE
SM: 0.59 BC: 0.57
Scanorama O2O
SM: 0.56 BC: 0.42
Scanorama SH
SM: 0.56 BC: 0.5
scANVI HE
SM: 0.83 BC: 0.82
scANVI O2O
SM: 0.82 BC: 0.81
scANVI SH
SM: 0.88 BC: 0.95
scVI HE
SM: 0.77 BC: 0.46
scVI O2O
SM: 0.75 BC: 0.29
scVI SH
SM: 0.76 BC: 0.46
SeuratV4 CCA HE
SM: 0.67 BC: 0.66
SeuratV4 CCA O2O
SM: 0.64 BC: 0.47
SeuratV4 CCA SH
SM: 0.66 BC: 0.6
SeuratV4 RPCA HE
SM: 0.62 BC: 0.6
SeuratV4 RPCA O2O
SM: 0.62 BC: 0.43
SeuratV4 RPCA SH
SM: 0.6 BC: 0.6
SAMap all genes
Species
H.sapiens
M.fascicularis
M.musculus
X.laevis
D.rerio
Cell types
B cell
cardiac muscle cell
dendritic cell
endothelial cell
erythrocyte
proliferating cell
Schwann cell
schwann precursor cell
smooth muscle cell
stromal cell
T cell
mast cell
mesothelial cell of epicardium
natural killer cell
pericyte cell
plasma cell
fat cell
fibroblast
granulocyte
innate immune epithelial cell
macrophage

Supplementary Figure 42 UMAP visualisation of integration results from 28 strategies in Heart_xl_dr task. Larger images available at the BENGAL reproducibility repository on GitHub [2]. UMAP, Uniform Manifold Approximation and Projection, SM, species mixing score, BC, biology conservation score, O2O, only uses one-to-one orthologs, HE, one-to-one orthologs plus one-to-many and many-to-many orthologs matched by higher average expression level, SH, one-to-one orthologs plus one-to-many and many-to-many orthologs matched by stronger homology confidence.

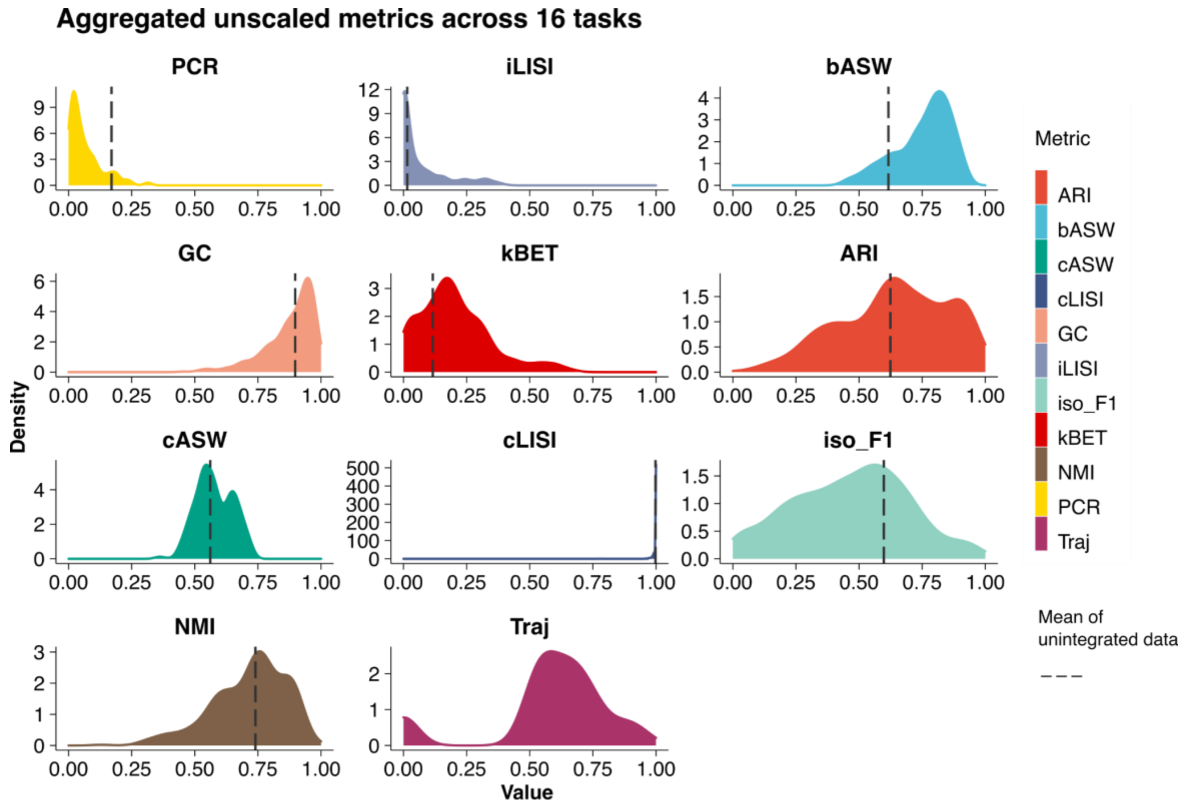

Supplementary Figure 43 Aggregated raw benchmarking metrics across 16 integration tasks. The distribution of raw metric scores across 16 tasks from 27 integration strategies and 3 types of homology concatenated, unintegrated data. iLISI and cLISI were removed in the end due to an uninformative range of variation. Batch corrections metrics include PCR; iLISI; GC; bASW; kBET; biology conservation metrics include ARI; NMI, cLISI; cASW; Traj. PCR, principal component regression; iLISI, batch graph Local Inverse Simpson's Index; bASW, batch average silhouette width;

GC, graph connectivity; kBET, k-nearest neighbour batch effect test; NMI, normalised mutual information; ARI, Adjusted Rand Index; cASW, cell type ASW; iso_F1, isolated label F1 score; Traj, trajectory conservation score.

Supplementary Methods

Principal of batch correction metrics

(1) Principal component regression (PCR)

Assume that there is batch effect in the dataset, then the heterogeneity caused by batch shall be captured by some principal components (PC) in the PC representation of the dataset. Regression of the batch covariant will then significantly correlate with some PCs. The sum of all variance contribution by batch from all PCs (Var (C|B)) gives an approximation of the level of batch effect in the dataset.

$$Var(B) = \sum_{i=1}^{G} Var(PC_i) \times R^2\ (PC_i|B) \tag{1}$$

The original PCR ranges from 0 to 1, with 0 means no batch contribution and 1 means the heterogeneity is entirely caused by batch. We take 1-PCR as the PCR score, so that it is in the same direction with other metrics: 0 means no batch integration and 1 means complete integration across batch.

(2) Batch average silhouette width (bASW)

Silhouette measures the relationship between with-in cluster distance of a cell and the between-cluster distance of that cell to the closest cluster. The batch ASW is computed based on the absolute silhouette of batch labels per cell i of cell type j, then averaged across M cell types.

$$bASW = \frac{1}{|M|}\sum_{j\in M}\frac{1}{|C_j|}\sum_{i\in j}(1 - |s(i)|)$$

(2)

Batch ASW range from 0 to 1, with 0 meaning strongly separated batches and 1 meaning ideal mixing of batch.

(3) Graph connectivity (GC)

The graph connectivity score measures whether the k-nearest neighbour (KNN) representation of the data connects all cells with the same label. GC is measured by the fraction of cells belonging to the largest connected component (LCC) of the subgraph constructed with all cells i with the same label j, averaged across M cell types.

$$GC = \frac{1}{|M|}\sum_{j\in M}\frac{|LCC(G(N_c; E_c))|}{|C_j|}$$

(3)

GC ranges from 0 (exclusive) to 1, with the lowest possible score meaning entire separation of cells with the same label in the graph and 1 meaning complete connection between cells with the same label.

(4) K-nearest neighbour batch effect test (kBET)

kBET examines whether a subset of neighbouring samples has the same distribution of batch labels as the full dataset. kBET uses a χ^2-based test for random neighbourhoods of fixed size to determine whether they are well mixed, followed by averaging of the binary test results to return an overall rejection rate. We calculate the kNN graph on different embeddings or the PCA embedding of pseudo-count matrices. We used k0 = 50 with lower and upper limits of k from 10-100 as in scIB (Luecken et al. 2021).

Since kBET score was assigned 1 to denote poor batch removal in scIB, we take 1-kBET score as the final kBET score to match the direction of all other metrics. kBET of 0 indicates

completely different distribution of local and global batch labels whereas 1 means equal distribution and well-mixed data.

Principal of biology conservation metrics

(1) Cell type ASW (cASW)

The cASW is to scale the ASW of cell types to a score between 0 and 1. 0 means there is higher intra-cluster heterogeneity and 1 means well-separated, clear cell types.

$$cASW = \frac{(ASW_c + 1\,)}{2} \tag{4}$$

(2) Normalised Mutual Information (NMI)

NMI describes the similarity of two clustering systems. Here we compare cell type annotation with optimised Louvain clustering as described in the scIB framework (Luecken et al. 2021). Leiden clustering was performed at a resolution range of 0.1 to 2 in steps of 0.1, and the clustering output with the highest NMI with the label set was used. NMI is scaled between 0 or 1, corresponding to uncorrelated clustering or a perfect match.

(3) Adjusted Rand Index (ARI)

ARI quantifies the overlap of two labelling systems. We calculate ARI between annotation and optimised Leiden clustering, the same as the clustering used in (3) NMI via the scIB framework (Luecken et al. 2021). ARI is scaled between 0 or 1, corresponding to random labelling or a perfect match.

(4) Isolated label F1 score (iso_F1)

Isolated labels are cell types that present in the least number of batches in the integration. The score evaluates how well these labels are separated from others. This is done by optimising

the cluster assignment of isolated labels by Leiden clustering and obtaining the optimum F1 score.

$$F1 = 2 \times \frac{precision \times recall}{precision + recall} \quad (5)$$

Iso_F1 is between 0 and 1 where 1 means that all the isolated label cells and no others are captured in the cluster, suggesting complete isolation.

(5) Trajectory conservation (Traj)

We used the diffusion pseudotime implemented in scanpy.tl.dpt from the SCANPY [3] package with default parameters to calculate pseudotime coordinate of cells. Blastula was used as the root cell for trajectory construction in the embryo_xt_dr task. The dpt score of each cell is given as input to scIB to compare pre and post-integration for each species. The trajectory conservation score is applicable to SAMap because the dpt is calculated from nearest-neighbour graphs.

Supplementary Notes

(1) Batch correction metrics and biology conservation metrics are not applicable for SAMap

SAMap [4] outputs a nearest-neighbour graph, which in theory can be evaluated by some of the above mentioned metrics, as they were adapted by scIB for graph type outputs [5]. However, SAMap was not designed as a batch correction method. In fact, it attempts to keep within species edges in the final graph to make sure the local topology around each cell is preserved. This is important especially when a cell type is connecting to multiple cell types or none in the other species. As SAMap treats intra and inter species edges differently, it is not suitable for batch effect metrics that operate on graph outputs. The scores would appear unreasonably low due to the preservation of within-species neighbours. Hence, we did not calculate the scores for SAMap and include it in the ranking, but instead report its output separately and facilitate comparison by showing the UMAP visualisations.

(2) Comprehending observed non-one-to-one cell type correspondence on integrated data

To perform a rigorous benchmark using consensus information, this study focused on one-to-one cell type mappings in vertebrates. A growing interest in the community is to use data integration to identify non-one-to-one mapped cells, to explore complex evolutionary relationship among cell types from different species. Below, we discuss the possible origins of the observed non-one-to-one mapping in one integration run, with an aim of advocating a more cautious manner towards drawing biological conclusions based on co-clustering.

An observed one-to-two mapping might arise from several technical and biological possibilities that would be difficult to distinguish with current tools and data. We focus on the possible technical reasons, mainly include a computational artefact or an experimental bias.

From the computational point of view, this could arise due to an integration error. Algorithms are prone to overcorrection and can map unrelated cell types. An example is the observed cell type merging by LIGER in main text Figure 3. Another possibility, is an annotation granularity inconsistency. Cell types may be annotated at different granularity between species, resulting in seemingly one-to-many mapping. For example, the endothelial cell of artery and endothelial cell of lymphatic vessels from the human heart data correspond to endothelial cells in all other species data in Supplementary Figure 20, upon which we found necessary to unify the annotation granularity among public datasets.

Experimentally, there could be a bias in cell type capture in one species. Several related populations in the other species thus seem to map to one cell type in the first species. It could also be due to an incomplete understanding of how many cell types exactly are there are in one species. Current species cell atlas projects are still underway and although they will eventually produce enough data points across organs and individuals to help resolve such issues, their datasets are still patchy. Furthermore, populations with a subtle difference in one species might not be separately annotated but can be distinguished in another species.

The possible biological reasons of non-one-to-one mapping on integrated scRNA-seq data includes a transcriptional convergence or an evolutionary homology [7]. In other words, the mapping of cell populations on the integrated data only suggests a transcriptomic similarity but not necessarily evolutionary relatedness. It is important to note that the scRNA-seq profile is only one phenotype of the cells. Consequently, any computational method that only operates only on scRNA-seq data cannot provide sufficient evidence to address evolutionary relatedness. At best, solely using scRNA-seq data we can generate hypotheses, but more evidence is required from multimodal data to examine evolutionary homology from shared transcriptional regulatory machinery[8] or other phenotypes. An example of cell type mapping on scRNA-seq data possibly due to transcriptional convergence is demonstrated by Woych, J. et al. [9] in mammalian neocortex and the dorsal ventricular ridge (DVR) of sauropsids.

Currently, the above-described biological possibilities cannot be disentangled by a standalone, available computational method, even when technical factors are considered.

In this benchmark, we focus on the computational artefacts introduced by scRNA-seq integration strategies. To assess this, we controlled over other possibilities. We unified the cell type annotation to a consistent granularity between datasets, guided by Cell Ontology, to provide a common ground for applying benchmarking metrics. To have confident evolutionary related cell types that should not be cross-matched, we focused on one-to-one homologous cell types in vertebrates supported by literature and have consensus among the community. Our focus on one-to-one homologous cell types aims to identify algorithms that yield more trustworthy cell type mappings without an integration error, in the most basic one-to-one mapping scenario.

Following these lines, if a top-performing method in this benchmark still generates a non-one-to-one mapping for a particular experiment, this could indicate that there is in fact possible biological correspondence. It is then sensible to continue investigating evidence of evolutionary correspondence using experimental data from other modalities.

(3) Other classifiers currently supported by ALCS

The current ALCS supports other machine learning models, such as Random Forest or Support Vector Machine, and all of them operate on feature-by-cell matrices. We used a logistic classifier because it showed comparable performance with non-linear models in the original SCCAF paper and offers interpretability if the model is trained using genes as features. The theoretical basis of ALCS is self-projection in machine learning. The focus lies in using self-projection to evaluate label quality in the dataset and the degree of separation between different labels. Consequently, changes in the relative accuracy of self-projection primarily stem from class assignment discrepancies. Although the specific classifier employed can impact absolute accuracy, its influence is limited when comparing between classifications. In the case of ALCS, it evaluates the quality of the embedding in terms of supporting the distinguishment of classes (cell types).

(4) Applying a kNN classifier for ALCS

Since some integration algorithms generate a corrected nearest-neighbour graph as integration output, we thought to explore the possibility of adapting a k-nearest neighbour (kNN) classifier for calculating ALCS. Although we did create a working prototype, we identified several concerns that suggest a significant amount of more work is required to properly extend to a kNN classifier for ALCS. In the following, we show our analysis and explain the concerns.

(a) Different kNN graph output generated by algorithms

For methods that generate a neighbour graph output, the final result is a connectivity matrix and possibly a distance matrix. For example, SAMap's primary output is an unweighted connectivity matrix while BBKNN [6] generates weighted connectivity matrix and distance matrix. Since there is no embedding for calculating distances, we thought to directly utilise these matrices in a kNN classifier. A weighted connectivity matrix and distance matrix for other algorithms that generate corrected counts or embeddings can be computed via sc.pp.neighbors(use_embedding=embedding_key, n_neighbors=K). We used the same number of neighbours for calculating a final kNN by different strategies (K=15). It is

important to point out that only the kNN output by SAMap is unweighted and makes it an outlier in this case.

(b) Applying a kNN classifier on distance matrices

To apply a kNN classifier on the distance matrix output by integration algorithms, we used the ad.obsp[‘distances’] matrix as a precomputed distance matrix to feed into sklearn.neighbors.KNeighborsClassifier. We continue using the self-projection principle of ALCS, that is to test how confused is the classifier on distinguishing the different cell types. In contrast with the current ALCS that utilises cell features, the evidence for a kNN classifier would be the classes of cell neighbours.

The first step is to split the training data and the test data per cell type. We divided the dataset into two independent sets by splitting the cells of each cell type by fraction x (x=0.5) for training and testing purposes. The kNN classifier was trained using the n_train-by-n_train matrix and subsequently tested on an n_test_by_n_train matrix to compute the test accuracy. The top K neighbours (K=15) served as the hyperparameter.

During this stage, we encountered the first issue related to the necessity of omitting part of the data due to the train-test split. The reason is that we cannot re-calculate the distances. Ordinarily, the distance between the test data and the training data is calculated from scratch, but we have to use the already-calculated distance matrix, resulting in ignoring some data. Supplementary Figure 44 illustrates this issue.

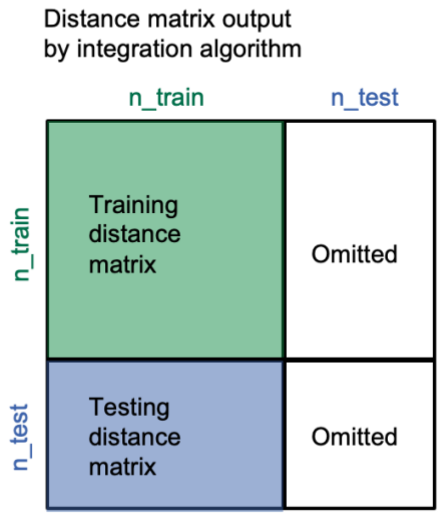

Supplementary Figure 44 Schematic of the train test split during application of a kNN classifier for ALCS. Due to the dimension constraints of the training and testing dataset, part of the data in the distance matrix is omitted. ALCS, accuracy loss of cell type self-projection; n_train, number of training samples; n_test, number of testing samples.

(c) Training and testing phases of the kNN classifier

In the training phase, the kNN classifier simply memorises the class labels of the n_train samples, as it is a non-parametric classifier. During the test phase, the model observes the supplied distances between the test samples and the training samples, selects the top K neighbours for each test sample, and determines the class of the test sample based on its k neighbours.

It is important to note that the prediction process essentially reduces to a weighted majority vote, with smaller distances conferring higher weights. Note that in ad.obsp['distances'], non-neighbours have a distance of 0 due to the design of the anndata object, so we replaced these distances with a large number (1e5) to assign a minimal weight, ensuring that they are not considered among the top k neighbours.

Since SAMap generates an unweighted connectivity matrix, an unweighted majority vote is performed for this algorithm. The classifier counts the top one most abundant classes among the neighbours of each test data and appoints this class as the prediction. The second concern that arises in this phase is that the model is non-parametric. There is no parameter learning from features that trains the model to distinguish different classes. This diverges from the principle of ALCS, with which we want to test how much biological information regarding the distinguishing features between cell types are maintained in the integrated data.

We still calculated the reduction of self-projection test accuracy between integrated data and per-species data as the final ALCS score. Supplementary Figure 45 shows the kNN-based ALCS score in different tasks:

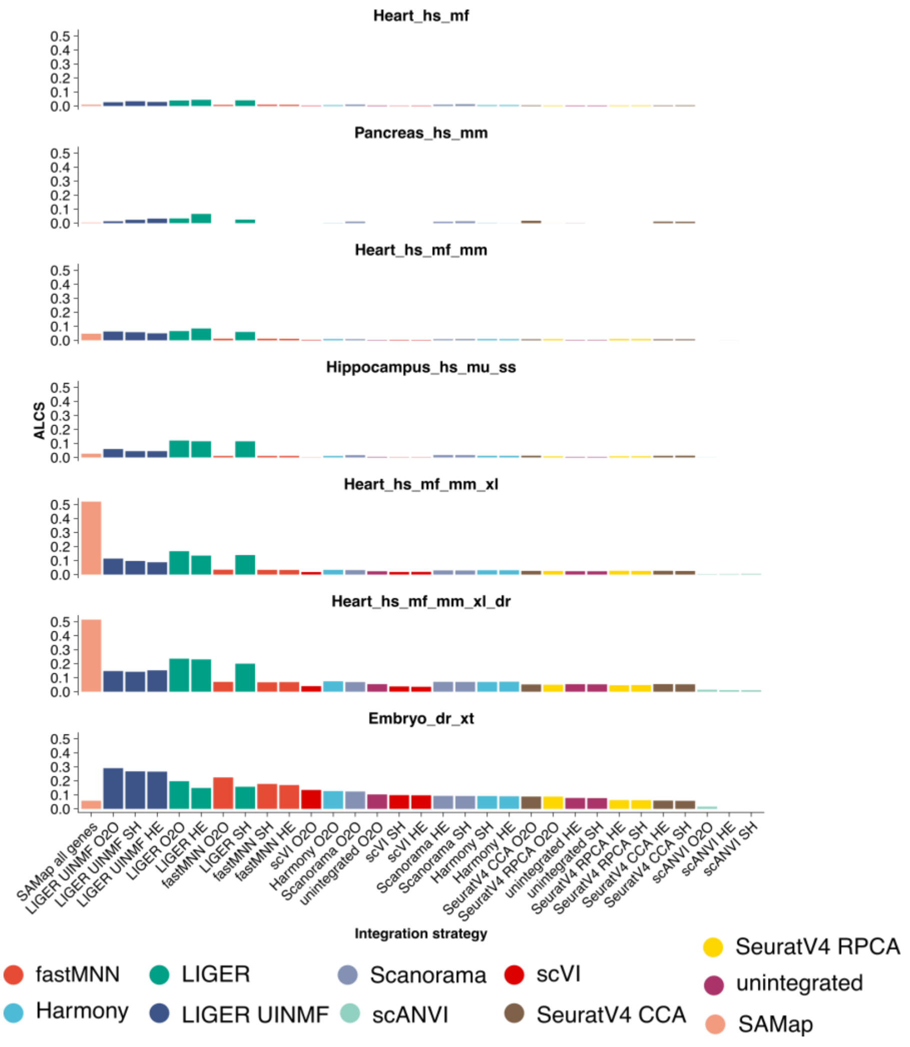

Supplementary Figure 45 The resulting ALCS score calculated using a prototype kNN classifier for all strategies in 7 reference tasks. ALCS, accuracy loss of cell type self-projection; O2O, only use one-to-one orthologs; HE, one-to-one orthologs plus one-to-many and many-to-many orthologs matched by higher average expression level; SH, one-to-one orthologs plus one-to-many and many-to-many orthologs matched by stronger homology confidence.

Results in Supplementary Figure 45 are in line with the previous version of ALCS shown in manuscript Figure 3, as well as the UMAP visualisations. ALCS sees a global increase with increased divergent time between species for all strategies. LIGER UINMF, LIGER and fastMNN generally have higher ALCS than other approaches. It is not a surprise that SAMap have higher ALCS in two of the heart tasks, because the algorithm is less effective on multiple datasets with many unshared cell types, in line with observed on UMAP. However, it is important to keep in mind that SAMap is an outlier in the sense that it generates an unweighted connectivity matrix.

(d) Conclusions on applying a kNN classifier for ALCS

Considering the aforementioned concerns, we feel that much more work is needed to properly adopt a kNN classifier for ALCS. Since the original ALCS with a logistic classifier already generated informative results and enables comprehension, we used it in the final manuscript.

Supplementary References

1. Osumi-Sutherland, D. *et al.* Cell type ontologies of the Human Cell Atlas. *Nat. Cell Biol.* **23**, 1129–1135 (2021).

2. Song, Y. BENGAL_reproducibility. *Reproducibility repository for BENGAL* https://github.com/Functional-Genomics/BENGAL_reproducibility.

3. Wolf, F. A., Angerer, P. & Theis, F. J. SCANPY: large-scale single-cell gene expression data analysis. *Genome Biol.* **19**, (2018).

4. Tarashansky, A. J. *et al.* Mapping single-cell atlases throughout Metazoa unravels cell type evolution. *Elife* **10**, (2021).

5. Luecken, M. D. *et al.* Benchmarking atlas-level data integration in single-cell genomics. *Nat. Methods* (2021) doi:10.1038/s41592-021-01336-8.

6. Polański, K. *et al.* BBKNN: fast batch alignment of single cell transcriptomes. *Bioinformatics* **36**, 964–965 (2020).

7. Arendt, D. *et al.* The origin and evolution of cell types. *Nat. Rev. Genet.* **17**, 744–757 (2016).

8. Arendt, D., Bertucci, P. Y., Achim, K. & Musser, J. M. Evolution of neuronal types and families. *Curr. Opin. Neurobiol.* **56**, 144–152 (2019).

9. Woych, J. *et al.* Cell-type profiling in salamanders identifies innovations in vertebrate forebrain evolution. *Science* **377**, eabp9186 (2022).
