# Supplementary material for: Benchmarking strategies for cross-species integration of single-cell RNA sequencing data
Source: Nat Commun. 2023 Oct 14;14:6495. doi: 10.1038/s41467-023-41855-w (PMC10576752; doi:10.1038/s41467-023-41855-w)
Supplement: Supplementary file 3 — Description of Additional Supplementary Files [file 41467_2023_41855_MOESM3_ESM.pdf]

File Name: Supplementary Data 1

Description: The raw and scaled benchmarking metrics, scores and their rankings from 27 integration strategies across 16 benchmarking tasks. Including 4 batch correction metrics and 6 biological conservation metrics. See details of metrics calculation in Supplementary Methods and scaling and aggregation in Methods. Results from three types of aggregations are provided: min-max scaling and averaging, excluding iLISI and cLISI (final method in this manuscript); aggregate unscaled metric including iLISI and cLISI; and aggregate unscaled metric excluding iLISI and cLISI. For the last two methods, the relative ranking among 27 strategies stayed largely consistent compared with results from the first method (Spearman's rank correlation coefficient $\rho=0.91\pm0.06$ across 16 tasks for aggregation with LISI scores, $\rho=0.93\pm0.05$ for those without LISI scores). This demonstrates the robustness of the scaling process and the stable relative performance among integration strategies.
