# Supplementary material for: Benchmarking strategies for cross-species integration of single-cell RNA sequencing data
Source: Nat Commun. 2023 Oct 14;14:6495. doi: 10.1038/s41467-023-41855-w (PMC10576752; doi:10.1038/s41467-023-41855-w)
Supplement: Supplementary file 5 — Reporting Summary [file 41467_2023_41855_MOESM5_ESM.pdf]

nature portfolio

Corresponding author(s): Yuyao Song, Irene Papatheodorou

Last updated by author(s): 18/08/2023

Reporting Summary

Nature Portfolio wishes to improve the reproducibility of the work that we publish. This form provides structure for consistency and transparency in reporting. For further information on Nature Portfolio policies, see our Editorial Policies and the Editorial Policy Checklist.

Statistics

For all statistical analyses, confirm that the following items are present in the figure legend, table legend, main text, or Methods section.

n/a	Confirmed	
☐	☒	The exact sample size (*n*) for each experimental group/condition, given as a discrete number and unit of measurement
☐	☒	A statement on whether measurements were taken from distinct samples or whether the same sample was measured repeatedly
☐	☒	The statistical test(s) used AND whether they are one- or two-sided<br>*Only common tests should be described solely by name; describe more complex techniques in the Methods section.*
☒	☐	A description of all covariates tested
☐	☒	A description of any assumptions or corrections, such as tests of normality and adjustment for multiple comparisons
☐	☒	A full description of the statistical parameters including central tendency (e.g. means) or other basic estimates (e.g. regression coefficient) AND variation (e.g. standard deviation) or associated estimates of uncertainty (e.g. confidence intervals)
☐	☒	For null hypothesis testing, the test statistic (e.g. *F*, *t*, *r*) with confidence intervals, effect sizes, degrees of freedom and *P* value noted<br>*Give P values as exact values whenever suitable.*
☒	☐	For Bayesian analysis, information on the choice of priors and Markov chain Monte Carlo settings
☒	☐	For hierarchical and complex designs, identification of the appropriate level for tests and full reporting of outcomes
☐	☒	Estimates of effect sizes (e.g. Cohen's *d*, Pearson's *r*), indicating how they were calculated

Our web collection on statistics for biologists contains articles on many of the points above.

Software and code

Policy information about availability of computer code

Data collection	No software was used for data collection in this study.
Data analysis	Software from published literature used in this study include: Python (v3.9.13), Scanpy (v1.9.1), h5py (v3.7.0), anndata (v0.7.5), harmonypy (v0.0.5), scanorama (v1.7.2), scVI and scANVI (v0.15.0 with pytorch v1.12.1 and cudatoolkit v11.6 to support execution with Nvidia GPU), SAMap (v1.0.2), scIB (v1.1.3), SCCAF (v0.0.10). R (v4.0.5), Seurat (V4.1.1), LIGER (v0.5.0), LIGER UINMF (v1.1.0), fastMNN (v1.12.3 from package batchelor). Nextflow (v22.04.3) DSL2 in java (OpenJDK v11.0.9.1-internal).<br>The BENGAL pipeline is available at https://github.com/Functional-Genomics/BENGAL and the version of code used in this study is available via Zenodo with DOI: 10.5281/zenodo.8268784. Code and source data for generating the figures in this study are deposited at https://github.com/Functional-Genomics/BENGAL_reproducibility; The package scOntoMatch is available through CRAN https://cran.r-project.org/web/packages/scOntoMatch/index.html, the version used in this study is 0.1.0 and the development version is available on GitHub https://github.com/Functional-Genomics/scOntoMatch.

For manuscripts utilizing custom algorithms or software that are central to the research but not yet described in published literature, software must be made available to editors and reviewers. We strongly encourage code deposition in a community repository (e.g. GitHub). See the Nature Portfolio guidelines for submitting code & software for further information.

Data

Policy information about availability of data

All manuscripts must include a data availability statement. This statement should provide the following information, where applicable:

- Accession codes, unique identifiers, or web links for publicly available datasets
- A description of any restrictions on data availability
- For clinical datasets or third party data, please ensure that the statement adheres to our policy

All datasets analysed in this study are publicly available. Raw count matrices and published annotations can be download from the following sources: inDrop data from human and mouse pancreas is available via the GEO database under accession code GSE84133 [https://www.ncbi.nlm.nih.gov/geo/query/acc.cgi?acc=GSE84133]; snRNA-seq data from human, macaque and pig hippocampal and entorhinal regions is available via the GEO database under accession code GSE186538 [https://www.ncbi.nlm.nih.gov/geo/query/acc.cgi?acc=GSE186538]; scRNA-seq data of heart and aorta tissue from human is available via figshare [https://figshare.com/projects/Tabula_Sapiens/100973], snRNA-seq data of the heart of long-tail macaque is accessible via the NHPCA database [https://db.cngb.org/nhpca/download], scRNA-seq data of mouse heart is available via the EBI ArrayExpress database under accession code E-MTAB-8810 [https://www.ebi.ac.uk/biostudies/arrayexpress/studies/E-MTAB-8810] (only no compound treatment mouse data was used) , microwell-seq data of Xenopus laevis heart is available via figshare [https://figshare.com/articles/dataset/Cell_Atlas_of_the_Xenopus_Laevis_at_Single-Cell_Resolution/19152839] and microwell-seq data of zebrafish heart is available via the ZCL database [https://bis.zju.edu.cn/ZCL/]; inDrops data of zebrafish embryo is available via the GEO database under accession code GSE112294 [https://www.ncbi.nlm.nih.gov/geo/query/acc.cgi?acc=GSE112294] and inDrops data of xenopus embryo is available via the GEO database under accession code GSE113074 [https://www.ncbi.nlm.nih.gov/geo/query/acc.cgi?acc=GSE113074]. Raw metrics, scaled metrics, scores and rankings for all tasks generated from this benchmark are provided in Supplementary Data 1.
Transcriptomes used in the BLAST step in SAMap are downloaded from ENSEMBL (v106, https://www.ensembl.org/index.html), except that for the Xenopus in embryo task is downloaded from Xenbase (https://www.xenbase.org/entry/static-xenbase/ftpDatafiles.jsp). Versions are in line with the version at original publication of each dataset: pancreas task: mouse (GRCm38) and human (GRCh38); hippocampus task: human (GRCh38), macaque (Mmul_10), pig (Sscrofa11); heart task: human (GRCh38), monkey (Macaca_fascicularis_6.0) and mouse (GRCm39); embryo task: Xenopus (Xtropicalisv9.0), zebrafish (GRCz10).

Human research participants

Policy information about studies involving human research participants and Sex and Gender in Research.

Reporting on sex and gender	There was no human research participants involved in this study.
Population characteristics	There was no human research participants involved in this study.
Recruitment	There was no human research participants involved in this study.
Ethics oversight	There was no human research participants involved in this study.

Note that full information on the approval of the study protocol must also be provided in the manuscript.

Field-specific reporting

Please select the one below that is the best fit for your research. If you are not sure, read the appropriate sections before making your selection.

☒ Life sciences ☐ Behavioural & social sciences ☐ Ecological, evolutionary & environmental sciences

For a reference copy of the document with all sections, see nature.com/documents/nr-reporting-summary-flat.pdf

Life sciences study design

All studies must disclose on these points even when the disclosure is negative.

Sample size	We analyzed 4 types of homology methods which covers all the possible appropriate ways to map genes across species via homology using ENSEMBL. The 10 integration algorithms studied in this benchmark encompass all currently available algorithms that have demonstrated top performance in previous benchmarks on same-species data. We compared 3 nearest neighbour-based algorithms with 6 other algorithms to address the impact of theoretical basis on integration performance in cross-species scenarios in Figure 6c.
Data exclusions	We excluded cells that didn't pass quality control in the original study and only analyze cells with published cell type annotation.
Replication	There were biological replicates in the Hippocampus_hs_mu_ss dataset from three species, and we performed cross-species integration at the replicate level for all strategies. The exact number of cells from each replicate is available in Table 1. Other datasets do not have replicates.
Randomization	Randomization is not relevant to this study. All algorithms were run on the same input datasets with the same pipeline and default parameters. A random seed was set so all algorithms generate reproducible results if run repeatedly.
Blinding	Blinding is not relevant to the study, there was no group allocation the investigator could be blind to.

Reporting for specific materials, systems and methods

We require information from authors about some types of materials, experimental systems and methods used in many studies. Here, indicate whether each material, system or method listed is relevant to your study. If you are not sure if a list item applies to your research, read the appropriate section before selecting a response.

Materials & experimental systems

n/a	Involved in the study
☒	☐ Antibodies
☒	☐ Eukaryotic cell lines
☒	☐ Palaeontology and archaeology
☒	☐ Animals and other organisms
☒	☐ Clinical data
☒	☐ Dual use research of concern

Methods

n/a	Involved in the study
☒	☐ ChIP-seq
☒	☐ Flow cytometry
☒	☐ MRI-based neuroimaging
